# Structural basis of DegP protease temperature-dependent activation

**DOI:** 10.1126/sciadv.abj1816

**Published:** 2021-12-08

**Authors:** Darius Šulskis, Johannes Thoma, Björn M. Burmann

**Affiliations:** 1 Department of Chemistry and Molecular Biology, University of Gothenburg, 405 30 Göteborg, Sweden.; 2 Wallenberg Centre for Molecular and Translational Medicine, University of Gothenburg, 405 30 Göteborg, Sweden.

## Abstract

Protein quality control is an essential cellular function mainly executed by a vast array of different proteases and molecular chaperones. One of the bacterial high temperature requirement A (HtrA) protein family members, the homo-oligomeric DegP protease, plays a crucial role in the *Escherichia coli* protein quality control machinery by removing unfolded proteins or preventing their aggregation and chaperoning them to their final folded state within the periplasm. DegP contains two regulatory PDZ domains, which play key roles in substrate recognition and in the transformation of DegP between inactive hexameric and proteolytic active cage-like structures. Here, we analyze the interaction and dynamics of the DegP PDZ domains underlying this transformation by high-resolution NMR spectroscopy complemented with biochemical cleavage assays. We identify an interdomain molecular lock, which controls the interactions between the two PDZ domains, regulated by fine-tuned temperature-dependent protein dynamics, and which is potentially conserved in proteins harboring tandem PDZ domains.

## Teaser

An interdomain lock modulates the functionality of the bacterial periplasmic protease DegP via distinct oligomeric states.

## INTRODUCTION

An effective protein quality control system consisting of molecular chaperones and proteases is essential for each living organism (*1*, *2*). Within the periplasmic space of Gram-negative bacteria, this crucial task is carried out mainly by the following three proteins: seventeen kilodalton protein (Skp), Survival protein A (SurA), and DegP (*3*). These proteins play a pivotal role in the quality control of outer membrane proteins and protect bacteria from the accumulation and aggregation of misfolded proteins within the periplasm. Skp and SurA are responsible for controlling protein folding through their function as holdase chaperones (*4*, *5*). In contrast, DegP has been shown to have a tightly regulated dual activity under different stress conditions in vivo (*3*): Primarily, it not only functions as a serine protease to rescue cells during heat stress, but it also has chaperoning activity at reduced temperatures (*6*). DegP was shown to be essential for *Escherichia coli* survival as a *degP* gene knockout is lethal at elevated temperatures (42°C) (*7*). However, it was possible to show that DegP protease activity is not a mandatory feature because overexpression of protease-deficient forms of DegP is already sufficient for *E. coli* to recover (*8*) under stress conditions, suppressing the lethal phenotypes (*9*).

**Fig. 1.**
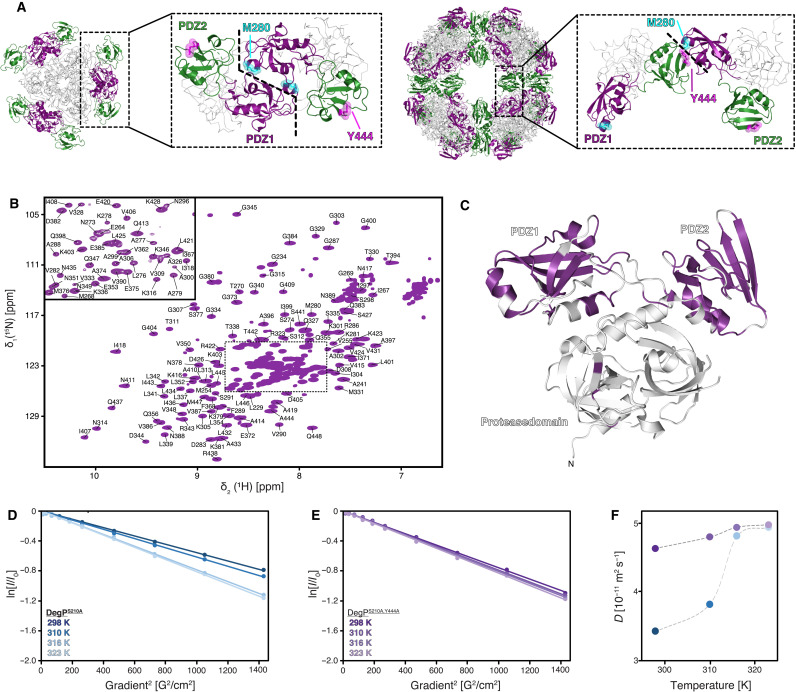
Full-length DegP^S210A^ in solution. (**A**) Comparison of the PDZ domain orientations between different monomers in the crystal structures of the hexamer (PDB ID: 1KY9) and the 24-mer cage assembly (PDB ID: 3OU0). In the inactive hexameric state, this interaction is proposed to be mediated mainly via a direct interaction between two PDZ1s, and the PDZ2s are found to be nonconstraint at the hexamer edges; however, within the proteolytically active 24-mer, the interaction between PDZ1 and PDZ2 of different molecules is achieved by a Y444 and M280 interaction, indicating stabilization by a sulfur-π-aromatic or a methyl-π-aromatic motif. (**B**) 2D [^15^N,^1^H]-NMR spectrum of [U-^2^H,^15^N,^13^C]-DegP^S210A,Y444A^. The sequence-specific resonance assignment obtained from 3D TROSY-type triple-resonance experiments is indicated. (**C**) Cartoon representation of a DegP^S210A^ monomer from the crystal structure of the hexameric assembly of DegP^S210A^ (PDB ID: 1KY9) with the sequence-specific resonance assignment indicated in purple. (**D** and **E**) Measurement of the molecular diffusion constant with a ^13^C-methyl–filtered diffusion experiment for DegP^S210A^ (D) and DegP^S210A,Y444A^ (E). The logarithm of the signal intensity is plotted against the squared gradient strength of the applied pulsed field gradients. The two [U-^2^H, Ile-δ_1_-^13^CH_3_]–DegP samples were measured at different temperatures as indicated. Solid lines are a linear fit to the data. (**F**) Obtained molecular diffusion constants were plotted against the temperature. The broken lines serve as a guide to the eyes only.

On a structural level, DegP consists of a protease domain connected to two adjunct PDZ domains named PDZ1 and PDZ2 after the first proteins PSD-95, Dlg1, and ZO-1, in which this type of domains was observed (*10*). In its protease-inactive state, DegP forms a homohexameric complex (*11*). Upon activation, DegP can rearrange into dodecameric or 24-mer cage-like forms, which are suggested to encapsulate substrate proteins before proteolytic cleavage (Fig. 1, A and B) (*12*–*14*). The common building block of the different cage-like structures is a trimer; therefore, DegP is assumed to undergo large structural transitions from its hexameric resting state via a trimeric intermediate state toward the higher oligomeric states (*15*, *16*). This structural transition itself is supposed to be mediated by modulations of intermolecular PDZ1-PDZ2 interactions (*17*). Moreover, several loops—termed LA, LD, L1, L2, and L3—play an important role in the regulation of the proteolytic function of DegP, having either activating or inhibitory roles (*12*, *18*). Among these, the so-called Loop A (LA) loop stands out as it is believed to stabilize the inactive conformation of DegP by shielding the active site within the protease domain in cellular ground states, e.g., in the absence of external stress stimuli. In addition, it crucially participates in the modulation of DegP function as mutations in this region directly affect the activation and functionality of the enzyme (*18*, *19*). Upon activation, the LA loop is supposed to facilitate local rearrangements, leading to the correct orientation of the catalytic triad ensuring effective proteolysis (*18*, *19*).

Nevertheless, the exact underlying mechanism and eventual structural adaptions remain only partially understood as previous structural studies could only reveal the respective end points of the different oligomeric assemblies. The current model suggests that peptides containing a hydrophobic C-terminal tail initially bind to the PDZ1 domain, thereby triggering subsequent structural rearrangements, which, in turn, activate DegP (*20*). Contrary, the PDZ2 domain inhibits protease activity as long as DegP disassembles into trimers and subsequently transforms into the cage-like structures (*17*).

To address the structural adaptions underlying temperature activation of DegP, we investigated full-length DegP and its isolated PDZ1-PDZ2 domains at the atomic level using high-resolution nuclear magnetic resonance (NMR) spectroscopy. On the basis of local chemical shift perturbations (CSPs), we identified an intermolecular methionine-tyrosine interaction controlling the transition from hexameric to trimeric DegP. Detailed analysis of the inherent protein dynamics revealed a finely tuned temperature-dependent allosteric network within PDZ1 modulated by the relief of the interaction with PDZ2. Biochemical characterization of the effect of this interdomain lock on the proteolytic function identified this temperature sensor as the first step in the activation cascade of DegP but not sufficient for complete protease activation. The crucial tyrosine methyl–bearing residues are conserved within high temperature requirement A (HtrA) proteins, in perfect agreement with the mechanism of inhibitory transient oligomerization recently identified for human HtrA2 (*21*), indicating a widespread fundamental activation mechanism modulated via an intermolecular lock between the PDZ domains.

## RESULTS

### Solution NMR of full-length DegP

Now, only static high-resolution structures of DegP by x-ray crystallography (*13*) and medium-resolution structures by cryo–electron microscopy (cryo-EM) (*22*) are available, providing an incomplete picture of the functional cycle of DegP. While these structures provide important insight into the organization of the different oligomeric states of DegP, the underlying structural transitions could not be deduced from them. Therefore, we investigated DegP by high-resolution NMR spectroscopy to obtain detailed insight into its structural and dynamical properties, which give rise to the different oligomeric states and coordinate the transition from the inactive to the active states of DegP. Because of its large size (~150 kDa as a trimeric building block) and the existence of different oligomeric forms (Fig. 1, A and B), DegP presents a challenging system for solution NMR studies. Here, we present the first [^15^N,^1^H]-NMR spectra of uniformly labeled [U-^2^H,^15^N]-DegP^S210A^, which has the active site serine mutated to prevent self-proteolysis (sequence numbering follows the mature proteins 1 to 448 excluding the N-terminal 26 residues long periplasmic targeting sequence) (fig. S1, C to E). The spectrum obtained at 25°C contained only a very limited number of resonances located exclusively in the central region of the spectrum, indicative of disordered and unstructured regions such as loops, in line with the large size of the protein (fig. S1C). In contrast, increasing the temperature to 37°C and subsequently to 50°C resulted in well-dispersed spectra containing approximately 120 resonances (fig. S1, D and E). The dispersion of these peaks [10 to 6 parts per million (ppm) in the ^1^H-dimension] showed that a folded domain of the full-length DegP was observable under these conditions, indicating its rather flexible attachment to the rest of the protein. In the next step, we used the known trimerization mutant DegP^S210A,Y444A^ (*23*), which resulted in further spectral improvement (Fig. 1C and fig. S1F) and allowed us to sequence-specifically assign large parts of the two C-terminal PDZ domains (150 of 188 PDZ1-PDZ2 resonances) using standard transverse relaxation-optimized spectroscopy (TROSY)–type three-dimensional (3D) experiments. The obtained secondary chemical shifts indicated that the structure in solution is, to a large extent, in agreement with the secondary structure elements observed previously within the different crystal structures (fig. S1, E to G). The observation of a large set of resonances of the DegP PDZ domains indicates that these domains are not in direct contact with other parts of the protein and tumble independently in solution. Furthermore, the increased spectral quality at elevated temperatures suggested that full-length DegP might transition between different oligomeric states, pointing to an inherent temperature switch for DegP activation.

To determine the oligomeric state of full-length DegP^S210A^ in solution, we initially tried to use the TROSY for rotational correlation times (TRACT) experiment (*24*) to probe the DegP size at the elevated temperatures. However, because of the lack of signals at lower temperatures, this approach could not be used at temperatures below 37°C (fig. S1H). Moreover, the inherent overestimation of flexible regions in the TRACT experiment prevented in-depth analysis (*24*). To circumvent these issues, we specifically labeled the methyl groups of the methyl-bearing isoleucine residues, which yielded high-quality spectra already at 25°C (fig. S1I) and measured pulsed field gradient diffusion experiments on the methyl groups (*25*). The obtained translational diffusion coefficients showed a clear temperature dependence (Fig. 1D). With increasing temperature, the viscosity- and temperature-corrected values increased from 3.4 × 10^−7^ cm^2^ s^−1^ at 25°C to 4.82 × 10^−7^ cm^2^ s^−1^ and 4.95 × 10^−7^ cm^2^ s^−1^ at 43° and 50°C, respectively (Fig. 1, D to F). Comparing these values to known literature values indicates that DegP is in a hexameric state [molecular weight (MW) = 300 kDa] at 25°C, whereas, at 43°C and higher, the protein is mainly in a trimeric state (MW = 150 kDa) (fig. S1J). At 37°C, we obtained diffusion coefficients that were slightly elevated compared to 25°C (Fig. 1, D to F), indicating the partial presence of a trimeric species as an initial step of the structural transition, which is estimated to be about 20 to 25% based on the increase in the diffusion coefficient. Our observation of a temperature-dependent disassembly of the DegP hexamer is in complete agreement with earlier size exclusion studies indicating the formation of trimeric DegP^S210A^ (~80% populated) at heat-shock temperatures of 42°C (*17*). To further verify this observation, we probed the effects of the point mutation DegP^S210A,Y444A^. In contrast to DegP^S210A^ this variant eluted from a size exclusion column as a mixture of trimer and hexamer already at 8°C (fig. S1D). To address the temperature-dependent modulation of the oligomeric states for this variant, we also used NMR diffusion measurements. With increasing temperature, the values only showed a slight variation, increasing from 4.64×10^−7^ cm^2^ s^−1^ at 25°C to 4.81×10^−7^ cm^2^ s^−1^ and 4.99×10^−7^ cm^2^ s^−1^ at 37° and 50°C, respectively (Fig. 1, E and F, and table S1). In comparison to DegP^S210A^, these values indicate that already at 25°C, the majority of this variant is in the trimeric state. The observation of trimerization upon mutating Y444 to alanine and the possible temperature-dependent release of the PDZ domains in wild-type DegP cannot be directly explained by the known hexameric DegP structure (Fig. 1A) (*13*). Within the larger oligomeric states, a domain lock is observed on the interface formed by Y444 and M280 (Fig. 1B) (*22*). To address whether this Y444-M280 interaction occurred as well in solution within the hexamer to trimer transition, we also tested the effect of mutating the corresponding methionine. Size exclusion chromatography revealed the existence of distinct trimeric and hexameric species for the DegP^S210A,M280A^ variant, in line with the hypothesis of a Y444-M280 interaction, modulating the transition of the oligomeric states (Fig. 1D). To gain additional insight into the nature of the interaction between these two crucial residues, we additionally tested the more conservative mutation variant DegP^S210A,M280I^. In contrast, this variant showed no distinct states in the size exclusion chromatography but rather a single elution peak in between the trimeric and the hexameric state, indicating a fast trimer-hexamer equilibrium suggesting altered kinetics compared to DegP^S210A,M280A^ (fig. S1F). In agreement with this modulation of kinetics, we obtained also a slightly altered hexamer-trimer transition when studying this DegP^S210A,M280I^ variant by NMR diffusion (fig. S1, K and L). In summary, our NMR diffusion measurements confirm a temperature-dependent dissociation of the DegP^S210A^-hexamer into its trimeric building blocks.

### Isolated PDZ1-PDZ2 bidomains in solution

On the basis of the initial observation of the highly flexible PDZ domains within the full-length DegP and their possible contribution to the inherent temperature switch, we subsequently focused on the isolated PDZ1-PDZ2 domains, to obtain detailed insight into the residues involved in this interaction. High-quality resonance assignment data of the PDZ1-PDZ2 domain construct allowed an almost complete sequence-specific assignment of the observed resonances in the [^15^N,^1^H]-NMR spectrum (fig. S2), yielding 87% backbone and 95% aliphatic side-chain assignment. The missing amino acids were part of the interdomain linker (356 to 366) or were located in flexible loops within PDZ1. This observed exchange broadening can be explained by enhanced amide-proton exchange rates at 50°C within these unstructured regions (*26*). Using the combined ^13^Cα and ^13^Cβ chemical shifts of the protein backbone, we identified the secondary structure elements of the PDZ1-PDZ2 domain construct in solution. The observed structural elements, 5 α helices and 10 β strands, are in agreement with the apparent secondary structure elements of the available crystal structures (fig. S2, C and D). However, we observed some differences within PDZ2, where the short helical turn α_4_ was slightly shifted and the strand β_8_ also lacked the characteristic secondary chemical shifts. Nevertheless, the presence of the short β strands, β_6_ and β_7_, in the linker region between the two PDZ domains, suggests that the domains are not completely decoupled, even at the elevated temperature of 50°C.

As we had observed large temperature-dependent effects within the spectra of full-length DegP, we also assigned the PDZ1-PDZ2 construct at 25°C and investigated its secondary structure propensities (fig. S3). The ^13^C secondary chemical shifts were almost identical to the ones obtained at 50°C, indicating that no temperature-dependent structural adaptions occurred within the PDZ domains. Nevertheless, a distinct lack of resonances was observed for residues within the amino and carboxy terminus of the PDZ1-PDZ2 domain construct in the 25°C dataset: the resonances for residues M268, G269, L272, L276, A277, M280, V282, R286, G287, S291, G307, G370, N435, I436, D440, and I443-M447 were broadened beyond detection in a [^15^N,^1^H]-NMR spectrum (fig. S3), in line with the presence of a dynamic intermolecular interaction between different PDZ1-PDZ2 molecules on the NMR intermediate time scale, i.e., with kinetic rates ranging from 1000 to 10 s^−1^ (*27*).

### Probing the interactions between PDZ1 and PDZ2

**Fig. 2.**
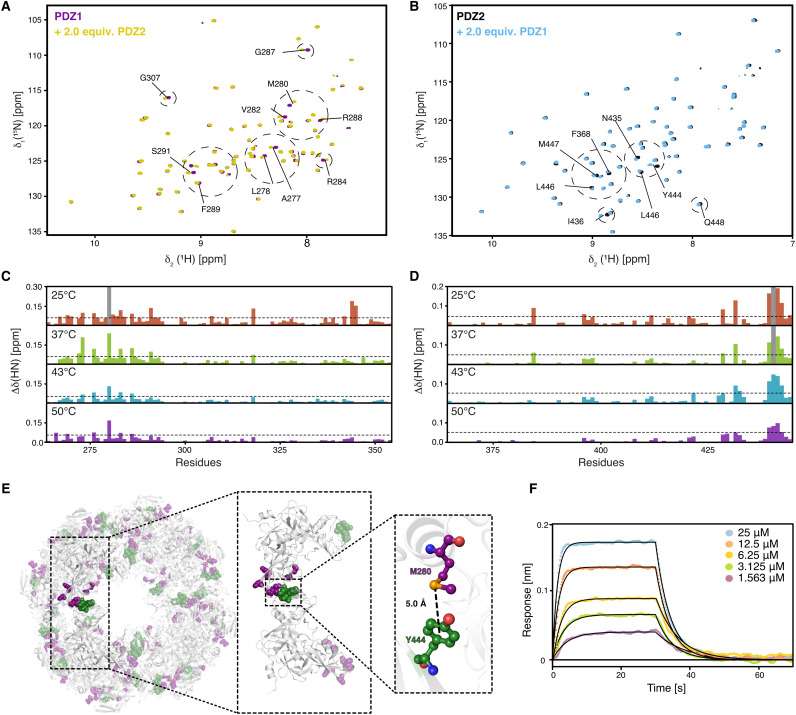
The individual PDZ domains show transient interactions reminiscent of the higher oligomeric DegP-complexes. (**A** and **B**) 2D [^15^N,^1^H]-NMR spectra of [U-^15^N]-PDZ1 [(A) purple)] and [U-^15^N]-PDZ2 [(B) black] before as well as after the addition of two molar equivalents of the respective other domain. Titrations were measured at 50°C. (**C** and **D**) Detected CSPs at four temperatures ranging from 25° to 50°C as indicated by the PDZ1 domain (C) and the PDZ2 domain (D). Severely affected residues experiencing line broadening are indicated as gray bars in the panels. The dotted lines represent a significance level of 0.05 ppm of the CSPs. (**E**) Perturbated resonances mapped on the crystal structure of DegP^S210A^ 24-mer (PDB ID: 3OU0). Mainly affected residues, CSPs twice the S.D. on PDZ1, are highlighted in purple (G266, G269, K278, M280, D283, and R286), whereas residues in PDZ2 are highlighted in green (A433, N435, I443, Y444, and L445). The enlargement focuses on the central M280-Y444 interaction at one of the interdomain interfaces. (**F**) Kinetic analysis by biolayer interferometry (BLI) of the PDZ1-PDZ2 interaction. PDZ1 binding to the biotinylated PDZ2 domain was probed at 25°C. Analyte concentrations are indicated in the figure. Nonlinear least-squares fits to the experimental data are indicated by black lines.

To follow up on the temperature-dependent line-broadening effect observed for the PDZ1-PDZ2 domain construct in solution, we characterized the PDZ1-PDZ2 interaction in detail further. Previous cryo-EM data indicated that the PDZ1-PDZ2 domains are essential for assembling and interlocking the DegP 12- and 24-mer cages and that removal of the PDZ2 domain prevents cage formation, altogether leading to primarily trimeric protein (*17*). Therefore, we prepared individual constructs of the PDZ1 and the PDZ2 domain and confirmed the sequence specific resonance assignments (fig. S4). To map possible interaction areas between these two isolated domains at different temperatures, we titrated the PDZ1 and the PDZ2 domains toward each other at four different temperatures (25° to 50°C). These temperatures represent the hexameric, the ~20% trimeric, and the fully trimeric state within the full-length DegP^S210A^ protein, as indicated by the NMR diffusion data (Fig. 1, D to F). The observed spectral changes indicated that the extreme N-terminal and C-terminal regions of the PDZ1-PDZ2 are most affected and therefore point to the possibility that the domains interact at 25°C. The CSPs decreasing with increasing temperature, with M280 showing the largest effect, are in perfect agreement with this interpretation (Fig. 2 and fig. S5). Completely in line with our previous observations for the larger DegP constructs, the affected resonances did not correspond to any interfaces on the available hexameric crystal structure (Fig. 1A) but were observed to be interfacial within the 12-mer x-ray and the 24-mer cryo-EM structures (Figs. 1B and 2E), indicating the possibility of a stabilizing sulfur-π aromatic or methyl-π aromatic motif (*28*, *29*). Moreover, a distinct set of residues showed larger CSPs along with an enhanced intensity loss at 25°C compared to higher temperatures (Fig. 2, C and D). Specifically, besides M280, Y444 was the residue most affected, implying that these two residues might provide the molecular switch locking the hexameric resting state at lower temperatures, completely in line with our observations by size exclusion chromatography and NMR diffusion for the different full-length protein variants (Fig. 1, D to F). To validate this hypothesis, we constructed the corresponding mutants (PDZ1^M280A^ and PDZ2^Y444A^) and evaluated whether the interaction between the domains was abolished. The titration experiments showed that the interaction was completely abolished if either M280 or Y444 was mutated (fig. S6, A and B), highlighting the involvement of both amino acids in forming the intermolecular lock. The same characteristic effect, although to a slightly lesser extent, was observed for the subset of peaks involved in the interaction for the PDZ^M280I^ variant, indicating that the complex was slightly less stable (fig. S6C).

To quantify the interaction between the individual wild-type PDZ domains, we used biolayer interferometry (BLI) steady-state analysis at 25°C resulting in a dissociation constant (*K*_d_) of 7.8 ± 0.9 μM for the binding of PDZ1 toward biotinylated PDZ2 (Fig. 2F and fig. S4C). We did not test the interaction vice versa with biotinylated PDZ1 in detail, because initial tests yielded a *K*_d_ weakened by a factor of ~10, indicating that the binding interface on PDZ1 is perturbed by the close proximity of the biotin label and the identified interaction site (fig. S4D).

### The M280-Y444 interaction locks the hexameric state

**Fig. 3.**
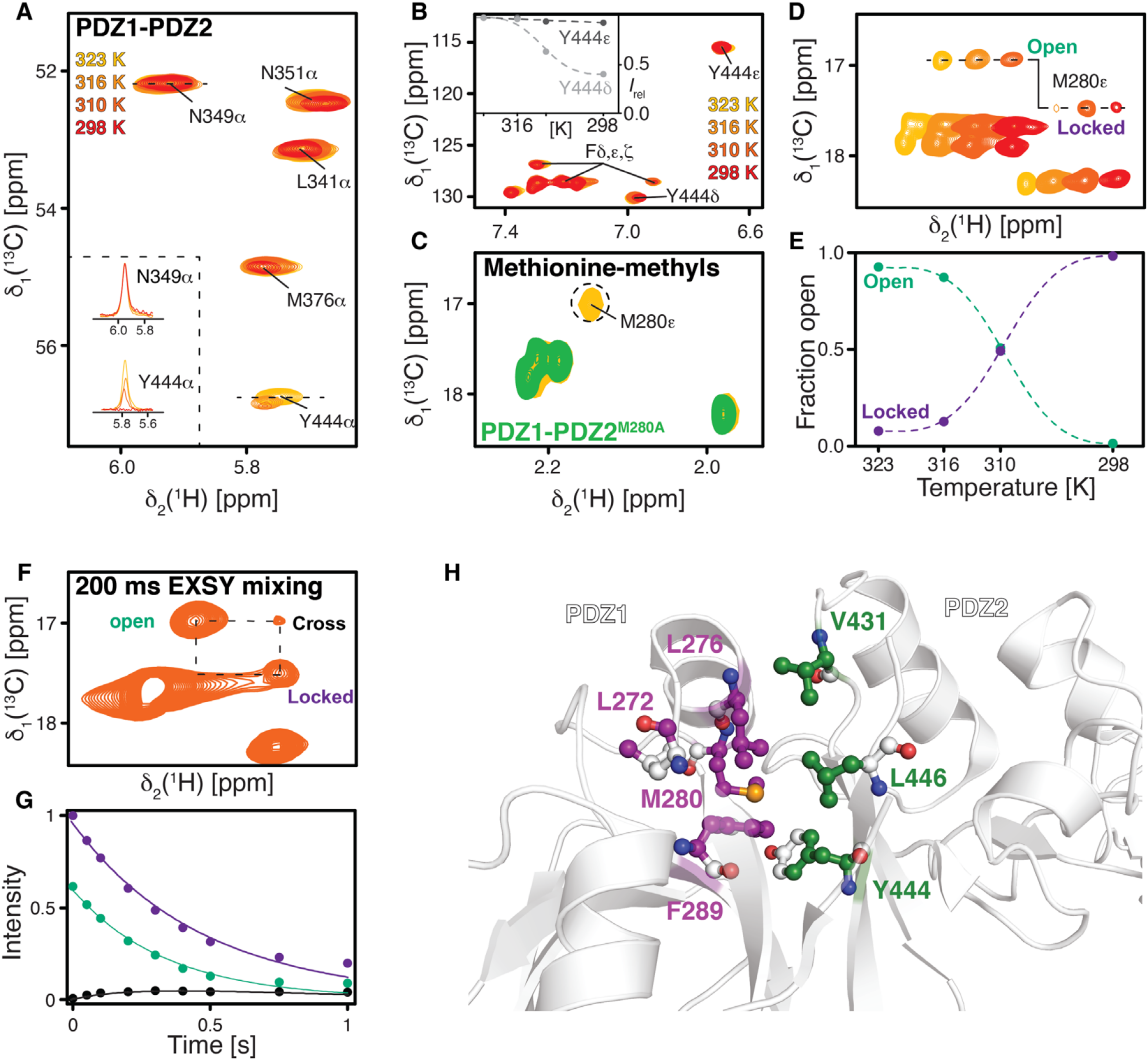
M280 is locking the PDZ1-PDZ2 interaction within DegP via Y444. (**A**) Focus on the Cα resonance region around Y444 of a 2D [^13^C,^1^H]-NMR spectrum of [U-^13^C,^15^N]-PDZ1-PDZ2 at 25° to 50°C as indicated. The inset shows the respective δ_2_[^1^H] 1D cross-sections through the N349α and Y444α resonances. (**B**) Aromatic region of a 2D [^13^C,^1^H]-NMR spectrum of [U-^13^C,^15^N]-PDZ1-PDZ2 at 25° to 50°C as indicated. Inset shows the signal intensities of the aromatic Y444ε and Y444δ resonances in the dependence of the temperature. (**C**) Overlay of [U-^13^C,^15^N]-PDZ1-PDZ2^M280A^ (green) and PDZ1-PDZ2 (yellow) at 50°C used to assign the M280ε resonance. (**D**) Methionine-ε-methyl region of a 2D [^13^C,^1^H]-NMR spectrum of [U-^13^C,^15^N]-PDZ1-PDZ2 at 25° to 50°C as indicated. Spectra were manually shifted along the ^1^H dimension to illustrate the transition between open and locked states. (**E**) Analysis of the open and locked state. Lines are a guide to the eyes only. (**F**) Magnetization exchange spectroscopy showing that the open state (o) and locked state (l) of M280 are in a dynamic equilibrium at 37°C as indicated. (**G**) The exchange rate (*k*_ex_) is determined from the buildup of the exchange peaks. (**H**) Observed NMR line broadening indicates a hydrophobic patch, stabilizing the PDZ1-PDZ2 interaction. Residues involved on the PDZ2 side are highlighted in green, whereas residues on the PDZ1 side are highlighted in purple.

To further analyze the basis of the M280-Y444 interaction, we initially checked the temperature effects on the resonance lines of the side chains within the PDZ1-PDZ2 construct. For Y444, we observed a Cα resonance line broadening to a similar extent than observed initially for the amide moiety (Figs. 2B and 3A). This behavior is furthermore reflected by the Y444δ ring resonances, which lose about 60% of their intensity at 25°C compared to 50°C in contrast to the Y444ε resonances, which show stable intensity over the tested temperature range (Fig. 3B). As the signal intensities were corrected for temperature-dependent changes before this analysis (see Materials and Methods for details), any remaining additional line broadening can readily be attributed to chemical exchange contributions. The observation of these localized effects centered around M280 and Y444 is thus perfectly in line with the temperature-dependent transient formation of a PDZ1-PDZ2 dimer at 25°C.

Subsequently, we investigated the effects on the M280 side, where we focused on its methionine-ε-methyl group, which we assigned by using the mutation variant PDZ1-PDZ2^M280A^ (Fig. 3C). Probing the M280ε resonance at different temperatures revealed the presence of two distinct states, which we termed open (50°C) and locked (25°C) (Fig. 3D). In the transition temperatures of 37° and 43°C, respectively, we could observe two distinct peaks for the open and locked conformations, which are in the slow exchange regime, milliseconds to seconds, on the NMR time scale. Integrating the peak intensities at the different temperatures revealed that, at 37°C, the open and locked conformation coexist in a 1:1 ratio, whereas, at 43°C or higher, the open conformation dominates and, at 25°C, the locked one, respectively (Fig. 3E). To quantify the interconversion between the open and the locked state, we used NMR exchange spectroscopy (Fig. 3, F and G) (*30*, *31*). We estimated the rate for the interconversion between the different states of *k*_ex_ = 0.33 ± 0.06 s^−1^ (SD) from the open to the locked state, although this value has to be considered as a rough estimate as the overlap of the second cross peak impaired a more detailed analysis. The observation of an opening mechanism on this slow exchange time scale is in line with recently reported data on a trimer-hexamer equilibrium for the related human HtrA2 (*21*).

To obtain a more detailed picture of the involved residues in the stabilization of this interdomain lock, we analyzed nuclear Overhauser enhancement spectroscopy (NOESY) cross peaks in a temperature-dependent manner (fig. S7). This analysis revealed that the participating residues—L276, M280, F289 within PDZ1, and V431, Y444, and L446 within PDZ2—are involved in stable nuclear Overhauser effect (NOE) networks in their respective domains over the temperature range 37° to 50°C. Because of the line broadening of the participating atoms, no intermolecular NOEs could be detected at 25°C, but the distinctive signal intensity loss showed the region’s involvement in this temperature lock by forming a hydrophobic patch, in agreement with the interface identified in the structures of the larger DegP assemblies earlier (Fig. 2E) (*22*). We also observed temperature-dependent line broadening for the methyl moieties of L272, which indicates its contribution to the interaction network. However, on the basis of the crystal structure of the DegP_12_ (*23*), this residue is not expected to be located directly within the binding patch, indicating a slight rearrangement of this part of the protein, potentially due to the isolated PDZ1-PDZ2 construct used.

### Backbone dynamics of the PDZ1-PDZ2 construct reveal a fine-tuned temperature adaption

**Fig. 4.**
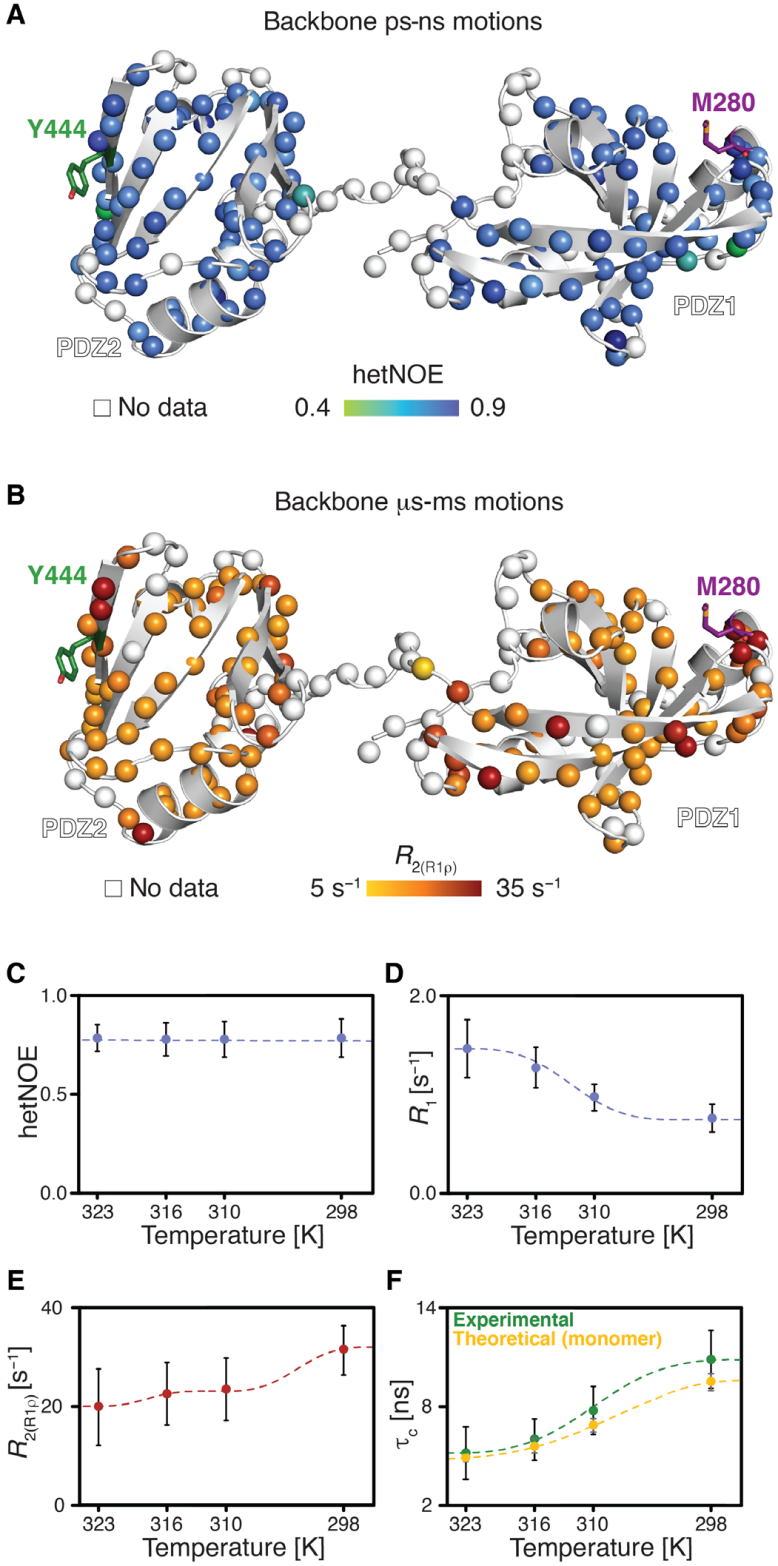
Backbone dynamics of the PDZ1-PDZ2 construct. (**A**) Local backbone dynamics on the pico- to nanosecond time scale probed by hetNOE measurements at 50°C. The amide moieties of the PDZ1-PDZ2 construct are shown as spheres and the hetNOE values are indicated by the green to blue gradient. (**B**) Obtained transversal relaxation [*R*_2(R1ρ)_] rates reporting on microsecond motions at 50°C, are plotted on the amide moieties and are indicated by the yellow to red gradient. (**C** to **E**) Temperature dependence of the average hetNOE (C), *R*_1_ (D), and *R*_2(R1ρ)_ rates (E) over the indicated temperature range. Error bars are the S.D. (**F**) Temperature dependence of the measured rotational correlation time, τ_c_, over the indicated temperature range (green). Theoretical values were calculated for the DegP crystal structure (PDB ID: 3OU0) with HYDRONMR (*70*) for a PDZ1-PDZ2 monomer.

To understand the molecular basis and eventual structural or functional consequences of the M280-mediated temperature switch in more detail, we next evaluated the inherent backbone dynamics of the PDZ1-PDZ2 domain construct over a broad range of time scales using NMR relaxation measurements (figs. S8 and S9) (*31*). To investigate the motions on the fast time scale (pico- to nanoseconds), the steady-state ^15^N{^1^H}-NOE (hetNOE) and the ^15^N longitudinal (*R*_1_) relaxation rates were measured. We observed a planar profile for the folded segments with average hetNOE values of 0.78 and *R*_1_ rates of 1.82 s^−1^, respectively, indicating a stable protein fold (Fig. 4A and fig. S8A). As expected, for the loop regions and the termini, we observed values indicative of increased flexibility, namely, lower hetNOE values and higher *R*_1_ rates. In line with our previous observation of secondary structure elements stabilizing the interdomain linker (fig. S2), we observed hetNOE values and *R*_1_ rates comparable to the folded segments, indicating that the PDZ1-PDZ2 construct tumbles in solution as a single entity.

Subsequently, we analyzed the contributions of motions in the micro- to millisecond regime. For these slow time scale motions, we analyzed the ^15^N transverse relaxation rates. First, we measured the *R*_2_ rates derived from the *R*_1ρ_ rates [*R*_2(R1ρ)_], which report on the motions on the lower microsecond time scale, because under the used spin-lock radio frequency field of 2000 Hz all exchange contributions (*R*_ex_) much slower than 80 μs would be leveled out (Fig. 4B and fig. S8B). In line with the previous analysis of the fast time scale motions, we observed a largely planar profile for the folded segments. In addition, we observed slightly enhanced transversal relaxation rates for the loop regions and for helix α_1_ as well as the C-terminal strand β_10_, containing M280 and Y444, respectively. Furthermore, we measured the transverse relaxation of the slowly relaxing ^15^N[^1^H] doublet component (*R*_2β_), revealing exchange contributions (*R*_ex_) on the higher micro- to millisecond time scale (*32*), which resulted in similar profiles as observed for *R*_2(R1ρ)_. In addition, by plotting the *R*_1_•*R*_2β_ values alongside the *R*_2β_ rates and observing the same trend in both datasets, we could rule out any contributions of large anisotropic diffusion tensor to the observed exchange contributions (*31*). In summary, the transversal relaxation rates indicate the existence of local conformational exchange contributions in the regions involved in the interdomain lock, e.g., helix α_1_ and strand β_10_.

Next, we assessed the obtained relaxation rates globally in a temperature-dependent manner (Fig. 4, C to F). Whereas we observed no differences in the hetNOE values over the tested temperature range, we could observe temperature-dependent modulations for *R*_1_ and *R*_2(R1ρ)_, resulting consequently in changes in the rotational correlation time τ_c_ (Fig. 4F). These observed changes in τ_c_ can be attributed to the following factors: changes in temperature and consequently solvent viscosity via the Stokes Einstein equation, leading to a change by a factor of 1.74, eventual decoupling of the individual domains, and/or transient intermolecular interactions. To delineate whether other contributions besides the temperature-dependent modulation play a role for the observed changes in τ_c_, we calculated the theoretical diffusion coefficients and compared them to our experimental data (Fig. 4F). There was a very good agreement at the higher temperatures, ruling out a meaningful contribution by domain decoupling under these conditions, whereas, at 25°C, the experimental and theoretical values deviate (the experimental values indicate a larger molecule size), pointing toward the formation of a transient interdomain interaction. To confirm our interpretation of a compacted PDZ1-PDZ2 state behaving as a single entity, we analyzed the *R*_1_/*R*_2(R1ρ)_ distributions over the temperature range. In line with our underlying assumption, we observed unimodal distributions at 50° and at 25°C, which is characteristic for a coupled domain movement (fig. S9A). The characterization of the *R*_1_/*R*_2(R1ρ)_ distributions provides an initial basis for the determination of the rotational diffusion tensor of a protein and in the case of multidomain proteins indicates either a globular and compact tumbling as a single entity by a unimodal distribution or alternatively a bimodal distribution in the case of decoupled domain movements (*33*, *34*). Our observations of the modulation of the relaxation rates are therefore in complete agreement with a temperature-dependent oligomeric transition as initially observed within the hexamer-trimer transition for full-length DegP^S210A^ by NMR diffusion measurements (Fig. 1, D to F).

**Fig. 5.**
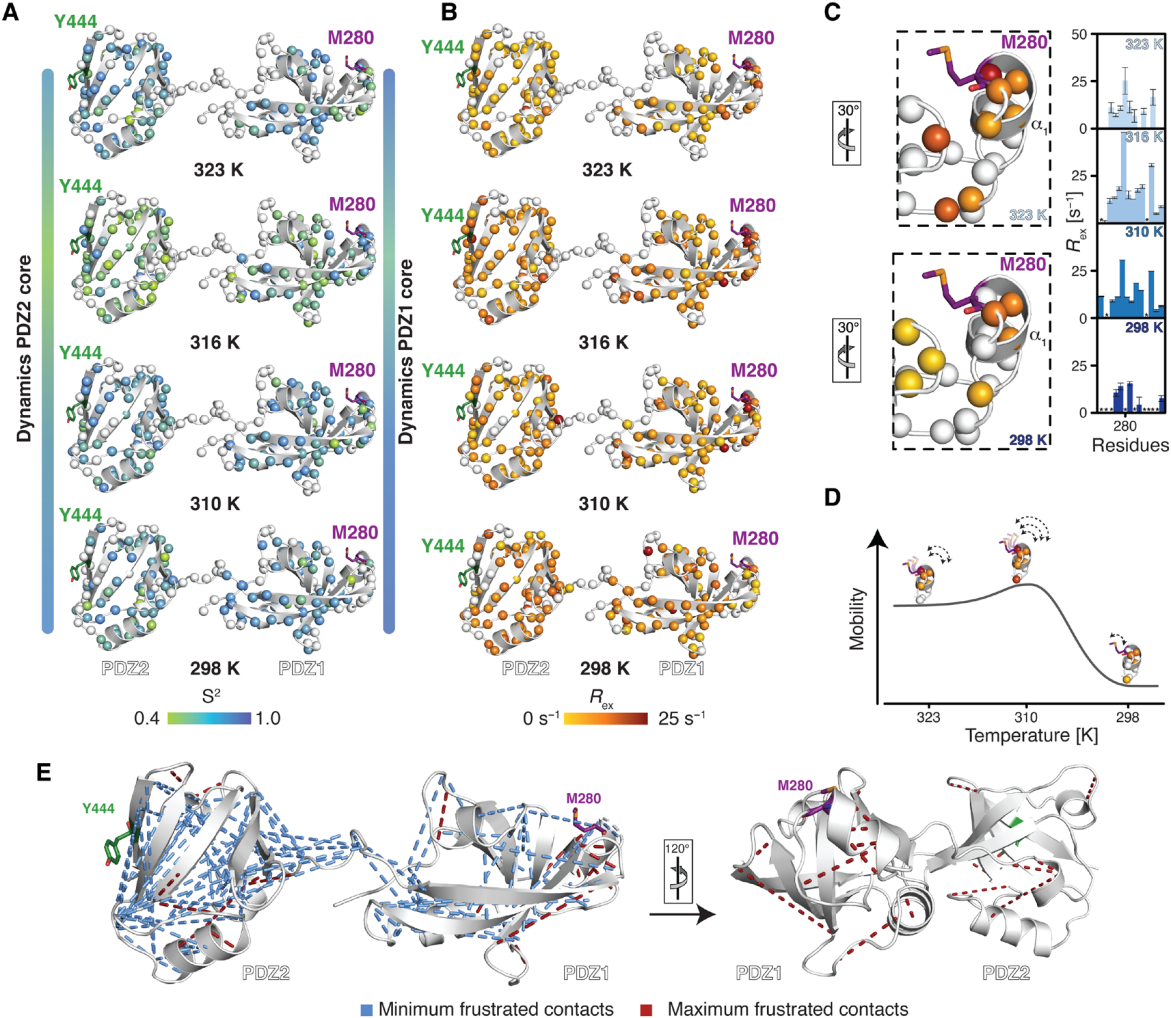
Backbone dynamics show correlated movements of PDZ1 and helix α_1_ harboring M280. (**A** and **B**) Temperature dependence of the subnanosecond order parameter S^2^ (A) and the exchange rate *R*_ex_ (B), reporting on the flexibility on the micro- to millisecond time scale, calculated using the Lipari-Szabo model-free approach (*35*, *36*, *80*). (**C**) Temperature dependence of the *R*_ex_ rates and zoom-in on the region around Met280. The rotation relative to (B) is indicated. The excerpt from the obtained exchange contributions (*R*_ex_) in this region is also shown. Residues for which no data could be obtained are indicated with a *. The full dataset is provided in fig. S9B. (**D**) Cartoon representation of the temperature dependence of the inherent mobility as reflected by the possible conformational exchange contributions on the micro- to millisecond time scale of helix α_1_ containing M280. (**E**) Frustration analysis was calculated for the DegP crystal structure (PDB ID: 3OU0) via the Protein Frustratometer 2 (*39*). Minimally frustrated interactions are indicated as blue lines, whereas highly frustrated interactions are indicated as red lines.

To obtain a more detailed picture of the roles of individual amino acids involved in the PDZ1-PDZ2 interaction, we used the model-free approach for the detailed analysis of the measured relaxation rates (*35*). Using an axially symmetric diffusion tensor (table S2) for the PDZ1-PDZ2 construct over the tested temperature range revealed distinct temperature-dependent modulations for the two domains (Fig. 4A) for the nanosecond time scale order parameters S^2^, reporting on the fast motions of the N─H vector. Whereas the core of PDZ1 (e.g., central β sheet) was showing only slightly temperature modulated S^2^ values of ~0.7, the core of PDZ2 showed lower values, with a minimum achieved at 43°C with ~0.6 for its central β sheet (Fig. 5A and fig. S9B). The exact cause for this increase in mobility at 43°C as evidenced by the drop of the S^2^ values together with enhanced *R*_ex_ contributions is not completely clear. However, the insinuation of a bimodal distribution of the *R*_1_/*R*_2(R1r)_ distributions (fig. S9A) suggests a partial decoupling of the two domains solely at this temperature, consequently suggesting that, at this temperature, the consideration of a PDZ1-PDZ2 single entity might not be completely valid.

Exchange rate contributions, *R*_ex_, which are additionally determined by the model-free approach (*35*, *36*), are indicative for possible effects of conformational exchange on the micro- to millisecond time scale. In agreement with the initially determined transversal relaxation rates, we observed particularly enhanced *R*_ex_ rates for the residues residing in helix α_1_ and parts of the β sheet core of PDZ1 pointing toward dynamical adaptions, enabling client interaction (Fig. 5B and fig. S9A). Assessing the *R*_ex_ rates in a temperature-dependent manner reveals that, at 25°C, possible conformational exchange contributions are minimized, especially around M280, whereas, from 37°C onward, micro- to millisecond motions manifest (Fig. 5, B and C). On the basis of the observed behavior, the picture emerges that the extend of motions of M280 is coupled to the release of the inter domain lock and, in this way, directly modulates the degree of motions within the whole PDZ1 domain (Fig. 5D and fig. S9A). Nevertheless, the observation that domain opening and enhanced backbone dynamics occur on different time scales indicates distinct separate underlying processes, such as domain release and substrate binding.

As the domain release had a direct effect on the apparent dynamics of the PDZ1, we wondered what might be the driving force for locking the domains in the inactive form. As, for example, molecular chaperones were previously shown to recognize locally frustrated regions (*37*, *38*), we hypothesized that local frustration within parts of the PDZ1-PDZ2 domains (*39*) might play a role for the observed intermolecular PDZ1-PDZ2 interaction (Fig. 5E). The most frustrated region as evidenced by the large number of highly frustrated contacts within the PDZ1-PDZ2 construct resides in helix α_1_ harboring M280. As local frustration arises from conflicts between the constrained connectivity of amino acids, like hydrophobic amino acids in close proximity to highly charged residues, resulting in energetically unfavorable local regions, this observation indicates that the M280-Y444 lock could be used to reduce this energetically unfavorable state in cellular ground states. In line with this reasoning, both the subnanosecond order parameter S^2^ and the exchange contributions *R*_ex_ determined via the model-free approach indicate the least extent of motions at the lower temperatures. In contrast under heat-shock conditions at 43°C, the largest degree of inherent motions may be observed, possibly pointing to the preparation of the PDZ1 for substrate interaction by modulating these dynamic properties in a temperature-dependent manner.

### Dynamics of the methyl side chains

To derive the origin of the enhanced local exchange contributions of helix α_1_ that provide the basis for modulating the PDZ1-PDZ2 interaction, we exploited the increased sensitivity of methyl groups to gain insight into side-chain dynamics. We chose specific labeling of isoleucine, leucine, and valine methyl groups as these amino acids are well dispersed among the PDZ domains, providing specific probes. First, we measured [^1^H,^13^C]-NMR spectra at different temperatures between 25° and 50°C (fig. S10, A and D). Comparing the spectra at 50° and 25°C revealed that a subset of the resonances underwent severe line broadening (e.g., L272δ1, L272δ2, L276δ1, L272δ2, I304δ1, I310δ1, I318δ1, I371δ1, I408δ1, V431γ1, and V432γ2) accompanied by CSPs (fig. S10, A to F). Whereas the latter can be attributed to a slight change in compactness of the protein core, which is also evidenced by the decreased extent of global backbone dynamics at the lower temperature (Fig. 5D), the not only the lack of signal intensity for a subset of methyl groups can indicate the presence of an intermolecular interaction but also the altered local dynamics are a potential cause.

**Fig. 6.**
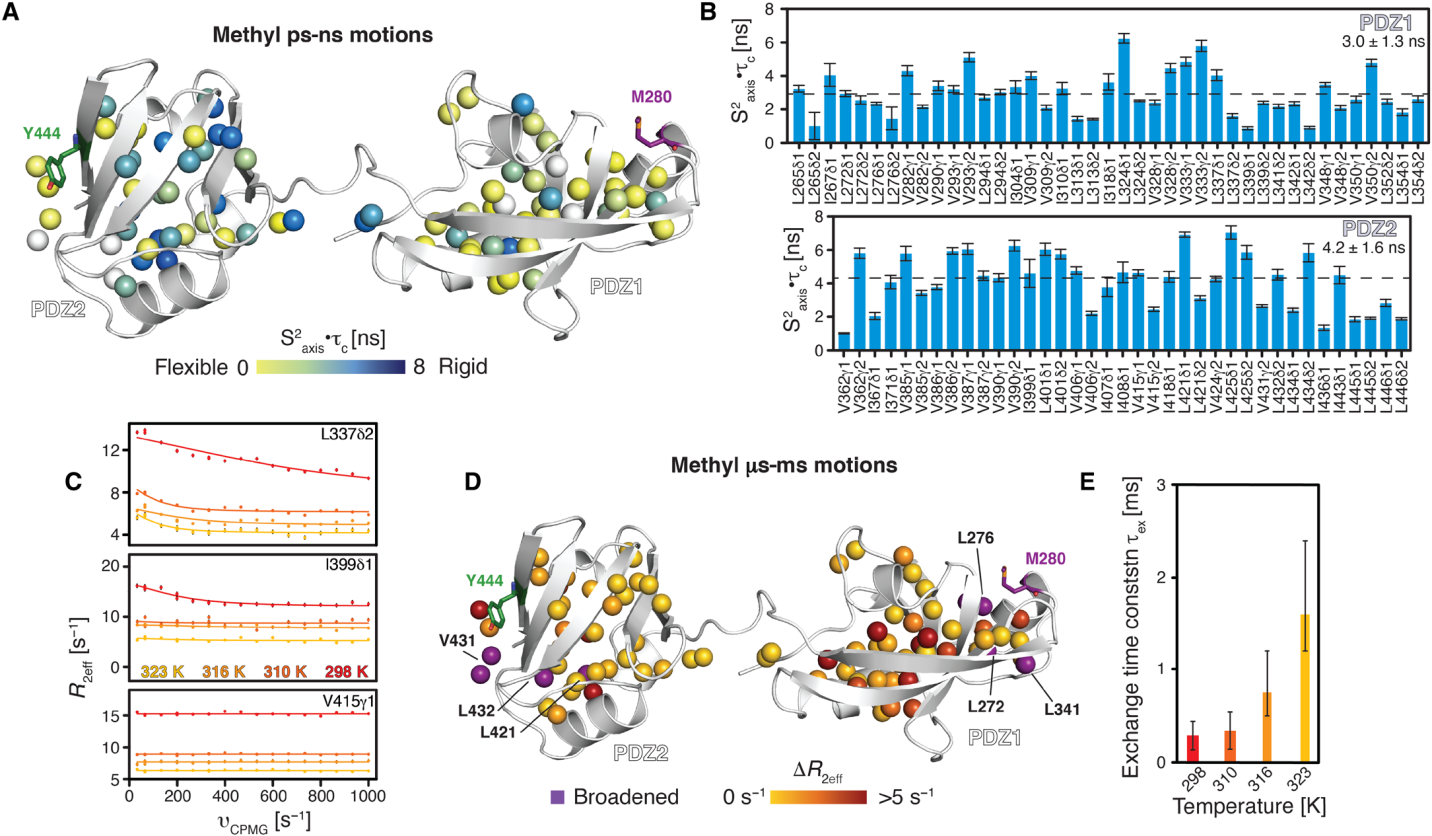
Isoleucine, Leucine, Valine (ILV) methyl groups reveal conformational exchange within the PDZ1 core. (**A** and **B**) Local methyl group dynamics on the pico- to nanosecond time scale probed by methyl single-quantum and triple-quantum relaxation experiments measurements (*71*, *72*) at 37°C showing the product of the local order parameter and the overall tumbling constant, S^2^_axis_·τ_C_. The ILV methyl groups of PDZ1-PDZ2 construct are shown as spheres, and the obtained S^2^_axis_·τ_C_ values are shown by the green to blue gradient (A). S^2^_axis_·τ_C_ values plotted against the sequence (B). (**C**) Exemplary ^13^C methyl MQ-CPMG relaxation dispersion profiles at different temperatures as indicated. Nonflat profiles indicate millisecond dynamics. (**D**) Structural view of the amplitude of the CPMG relaxation dispersion profiles Δ*R*_2eff_ at 25°C. (**E**) Rate constants of the dynamic process (τ_ex_) obtained from a global fit of the CPMG relaxation dispersion data.

To assess the nature of the underlying motions, we first determined the product of the side-chain order parameters and the correlation time of the overall molecular tumbling (S^2^_axis_·τ_C_) for an isoleucine-labeled and a leucine, valine-labeled PDZ1-PDZ2 sample, providing insight into the extent of the amplitude of motions of the methyl axis on the fast NMR time scale (Fig. 6, A and B). The obtained residue-wise profiles at 37°C indicate a large degree of flexibility for the studied methyl groups. Whereas groups residing in the core of the PDZ2 domain show overall a lesser extent of motions on this time scale, reflected by an average S^2^_axis_·τ_C_ = 4.2 ± 1.6 ns, methyl groups in the core of the PDZ1 uniformly show a higher degree of mobility with S^2^_axis_·τ_C_ = 3.0 ± 1.3 ns.

As several methyl resonances appeared to be involved in conformational exchange processes, we next quantified these by using multiple quantum (MQ) Carr-Purcell-Meiboom-Gill (CPMG) relaxation dispersion measurements (*40*), sensing motions on the micro- to millisecond time scale. Measuring CPMG experiments at temperatures ranging from 25° to 50°C revealed that the residues experiencing micro- to millisecond motions reside mainly within the PDZ1 domain, namely, methyl groups V282γ2, V309γ2, I310δ1, L313δ1, I318δ1, L337δ2, L339δ2, L341δ1, L352δ2, and L354δ2, as evidenced by nonflat dispersion profiles (Fig. 6, C to E, and fig. S10G). Quantitative global analysis of the dispersion data showed that this motion occurred on a 0.2-ms (25°C) or 0.4-ms (37°C) time scale under inactive conditions and 1-ms (42°C) or 2-ms (50°C) time scale under activating heat-shock conditions, indicating the priming of the substrate binding interface within the PDZ1 domain (Fig. 6E).

**Fig. 7.**
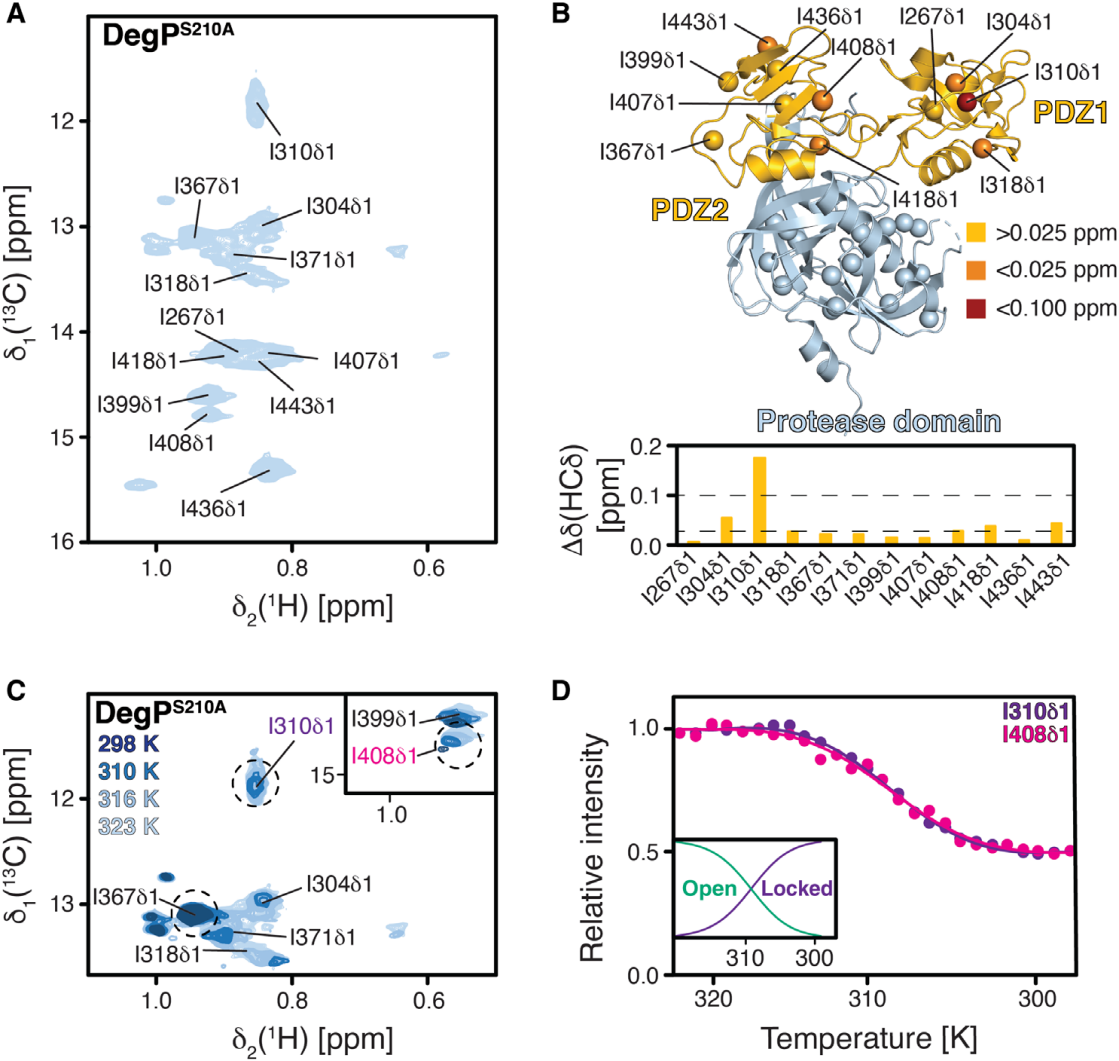
Isoleucine methyl groups within full-length DegP show also temperature-dependent transition. (**A**) 2D [^13^C,^1^H]-NMR spectrum of [U-^2^H, Ile-δ_1_-^13^CH_3_]–DegP^S210A^ at 50°C. The indicated resonance assignments were transferred from the assigned PDZ1-PDZ2 construct. (**B**) Chemical shift changes of the isoleucine methyl groups of DegP compared to the PDZ1-PDZ2 (bottom). The isoleucine methyl groups of one DegP monomer are indicated with spheres (top), and the chemical shift changes compared to the PDZ1-PDZ2 construct are indicated by the yellow to red gradient. (**C**) Excerpt of the isoleucine methyl region of a 2D [^13^C,^1^H]-NMR spectrum of [U-^2^H, Ile-δ_1_-^13^CH_3_]-DegP^S210A^ at different temperatures as indicated. (**D**) The temperature dependences of the selected I310δ1 and I408δ1 signal intensities are shown. Solid lines represent a fit to a two-state model for the extraction of estimates of thermodynamic parameters. The inset shows the respective populations of the open (trimer) and the locked (hexamer) state, respectively, at the different temperatures.

Having established the inherent mobility on the micro- to millisecond time scale of mainly the PDZ1 domain, we next set out to probe these effects on the isoleucine methyl probes within the full-length DegP^S210A^. On the basis of the side-chain assignment of the isolated PDZ1-PDZ2 domain construct (fig. S2E), we were able to unambiguously transfer the assignment of all 11 resonances within the DegP^S210A^ full-length construct (Fig. 7, A and B). To study the transformation from the low-temperature hexameric state to the high-temperature trimeric state within full-length DegP^S210A^, we measured [^1^H,^13^C]-NMR spectra over the complete temperature range from 25° to 50°C (Fig. 7C). At the low temperatures, several residues (e.g., I310δ1 and I408δ1) show reduced intensity comparable to the situation observed for the PDZ1-PDZ2 construct. Using the measured intensities for I310δ1 and I408δ1 from 25° to 50°C together with transition state theory, we extracted thermodynamic values for the transition from the locked (hexamer) to the open (trimer) state assuming the absence of any intermediate or unfolded states contributing to the transition over the analyzed temperature range (see Supplementary Note for details on the underlying assumptions and limitations). Fitting the data to a two-state model yielded an activation enthalpy of Δ*H* = 241.7 ± 88 kJ mol^−1^ and entropy of Δ*S* = 6.56 ± 1.18 kJ mol^−1^ K^−1^ with a transition midpoint at 36.8° ± 1.3°C. The obtained activation enthalpy is perfectly in line with the obtained values for a general methyl-phenylalanine or a comparable methionine-phenylalanine interaction motifs of 50 to 100 kJ mol^−1^ (*41*, *42*), providing further indication that the Met-Tyr motif is stabilizing the intermolecular PDZ1-PDZ2 interaction.

Similar to the initial observations for the [^1^H,^15^N]-NMR spectra (fig. S1), the [^1^H,^13^C]-NMR spectra of the isoleucine labeled DegP^S210A^ also did not yield high-quality spectra for the protease domain of full-length DegP (Fig. 6). This suggests that a high amount of inherent dynamics exists for this domain (backbone and methyl groups), which could already be observed to cause poor quality NMR spectra for some oligomeric proteases earlier (*43*, *44*), and therefore likely reflects the regulatory role of the variety of different loops involved in the regulation of DegP protease function (fig. S1, A and B). In agreement with this reasoning is the observation of [^1^H,^13^C]-NMR spectra of similar quality for isoleucine labeled DegP^S210A,Y444A^ and DegP^S210A,M280I^ over the tested temperature range (fig. S11, A to C). Whereas these spectra differ in terms of quality for the resonances of the two PDZ domains, with DegP^S210A,Y444A^ providing the highest quality spectra, reflecting its obligate trimeric form over the whole temperature range assessed, no distinctive differences for isoleucine residues originating from the protease domain could be observed.

### DegP trimers display increased proteolytic activity

**Fig. 8.**
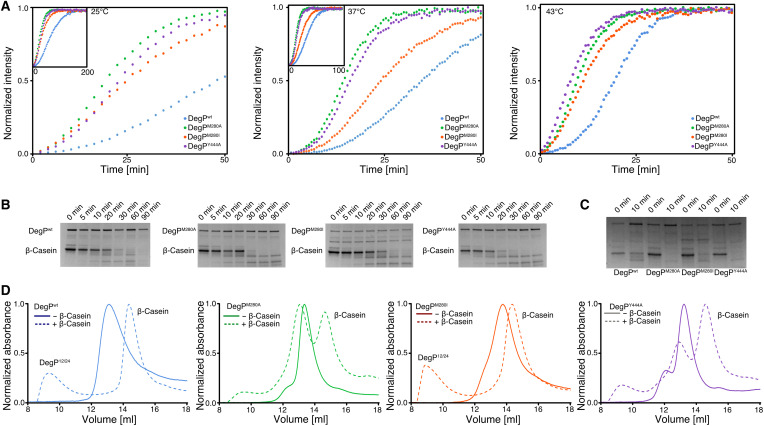
Assessing the effect of the domain lock on DegP proteolytic function. (**A**) DegP (10 μM monomer concentration) proteolysis of β-casein (50 μM) via detection by a fluorescent nonactivating reporter peptide (100 μM) at 25°, 37°, and 43°C. All experiments were scaled to the experiment at 43°C. Insets show the respective full datasets at the lower temperatures. Experiments were performed as triplicates yielding identical results. (**B**) SDS–polyacrylamide gel electrophoresis (PAGE) analysis of β-casein (5 μM) cleavage by DegP variants (1 μM monomer concentration) at the indicated time intervals at 25°C. (**C**) SDS-PAGE analysis of β-casein cleavage at the indicated time intervals at 37°C. Experiments were performed as triplicates, yielding identical results. (**D**) Size exclusion chromatography elution profiles of DegP and β-casein mixtures on an Superdex 200 Increase 10/300 column (GE Healthcare).

To investigate whether DegP trimerization has a direct influence on the proteolytic function, we tested the effect on previously reported DegP model substrates: a fluorescent p23 peptide, a fluorescent nonactivating reporter peptide, and β-casein. In initial test experiments, we were not able to observe notable differences for the DegP variants cleaving the fluorescent p23 peptide (fig. S12), which can likely be attributed to the proposed function of this peptide as a DegP activator (*23*). On the other hand, the used DegP mutants, DegP^Y444A^, DegP^M280A^, and DegP^M280I^ did cleave the fluorescent nonactivating reporter (*14*) faster in a coupled assay when activated by β-casein (Fig. 8A). The observation of sigmoidal curves for all tested DegP variants indicates the necessity of a second activation step, besides breakage of the PDZ1-PDZ2 interaction, for its proteolytic function, likely pointing to a positive cooperative allosteric activation of the LA loop upon β-casein binding to PDZ1 (*12*, *18*, *23*). The DegP^M280I^ variant showed an intermediate behavior in our assays, suggesting a partially functioning interdomain lock, albeit being unexpectedly active at 25°C compared to 37°C.

Analyzed by SDS–polyacrylamide gel electrophoresis (SDS-PAGE), DegP^M280A^ and DegP^Y444A^ variants completed cleavage of a 30× molar excess (considering a DegP hexamer) of β-casein within 30 min, whereas DegP and DegP^M280I^ required up to 1 hour to process β-casein (Fig. 8, B and C). It should be noted that it should be possible for DegP^M280I^ to form 12- and 24-mer cages due to interactions between the long methyl-bearing isoleucine side chains and the aromatic π-system of Y444 (*29*). This feature could be confirmed by size exclusion chromatography, where DegP and DegP^M280I^ formed almost exclusively cage-like structures, reflecting DegP cages in the presence of the β-casein substrate, whereas the capability to assemble these cages was largely diminished in DegP^M280A^ and DegP^Y444A^, indicating the importance of the PDZ1-PDZ2 interdomain stabilizing the large assemblies (Fig. 8D).

Additional NMR titrations provided evidence for our initial reasoning that p23 primarily binds to the PDZ1 domain and thus likely triggers the allosteric changes needed for proteolytic activation via the LA loop in complete agreement with previous crystallographic studies (*23*) (fig. S12, A to D). β-casein, on the other hand, had a more global effect on both domains at half equimolar ratio, reflecting its larger size (24 kDa) and consequently larger binding interface compared to the p23 peptide (fig. S12D). At lower ratios, the largest CSPs were observed for the PDZ1 domain, in agreement with its role as the main substrate recognition region and indicating an auxiliary role for the PDZ2 in substrate recognition (fig. S12, D and E). Last, the nonactivating reporter peptide did not lead to any substantial spectral changes, indicative of binding to the protease domain (*45*) and thus highlighting the need for allosteric activation of DegP through substrate binding to the PDZ1 domain, a characteristic feature for a variety of HtrA proteins (*11*, *14*, *20*, *46*, *47*).

## DISCUSSION

Despite being studied extensively and the existence of several high-resolution crystal structures of DegP in its different states, multiple questions remain about how its structural transitions are achieved. Within the known structures of the hexameric state, the DegP_12_ and DegP_24_ structures, an intermolecular PDZ1-PDZ2 interaction always coordinates the arrangements of the DegP trimer building blocks to stabilize the larger oligomeric assemblies (*12*). Thereby, the structures indicated a different arrangement of the PDZ domains for the hexameric state compared to the larger oligomeric states (Fig. 1A). However, our solution NMR results presented here show that, in solution, DegP is locked in the hexameric state via the same M280-Y444 interaction as observed initially within the larger oligomeric states, indicating that this interaction is crucial for modulating the different oligomeric states within DegPs functional cycle. Although the participation of Y444 was already suggested on the basis of the characterized DegP^S210A,Y444A^ mutant (*14*), our data revealed that the M280 is not only participating in this interaction but also effectively modulating the temperature-dependent opening of this interdomain lock.

Studying backbone in combination with methyl group side-chain dynamics over the transition temperature range revealed inherent dynamics within the core of the PDZ1 domain (V282, V309, I310, L313, I318, L337, L339, L341, L352, and L354), which are potentially allosterically triggered by the domain release. The conformational transition of M280 between its locked and open state is on a different time scale, therefore only indirectly modulating these inherent dynamics occurring on a faster time scale. Our data indicate that this intermolecular interaction is stabilized likely via a sulfur-π interaction of the M280 and Y444 side chains, a motif lately receiving recognition as an important stabilizer of protein interactions (*28*, *41*). Although we cannot completely rule out a methyl-π interaction, the fact that a sulfur-π interaction is believed to be the stronger interaction (*41*, *42*) and our observation that the conservative mutation M280I leads to a destabilization of the interdomain lock suggests a sulfur-π interaction.

**Fig. 9.**
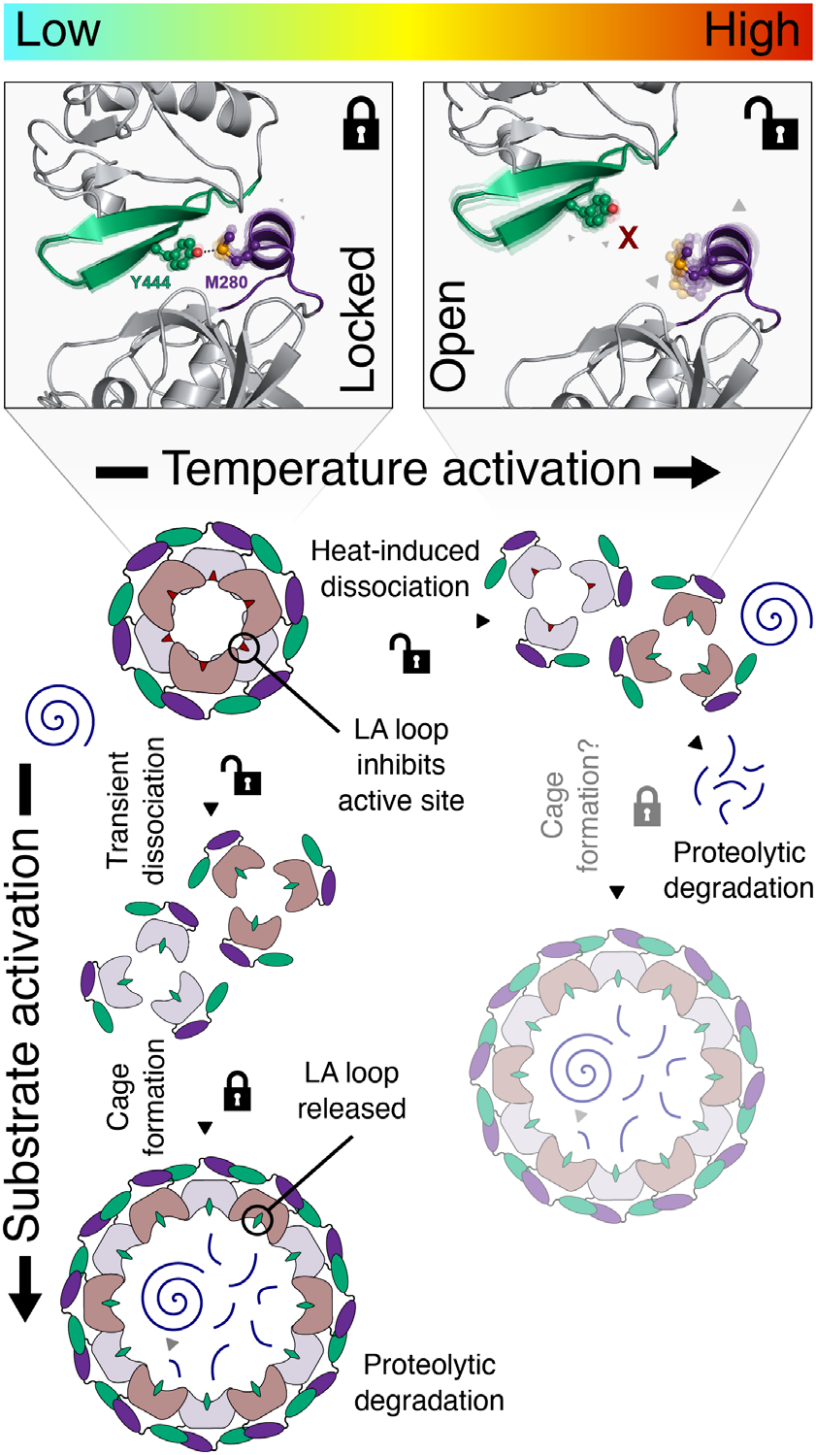
Temperature activation of the DegP protease mediated by the M280-Y444 interdomain lock. In cellular ground states at low temperature, the dynamics of M280 are reduced to stabilize the PDZ1-PDZ2 interaction via the sulfur-π interaction with Y444 (locked state), resulting in an inactive DegP hexamer. Under heat-shock conditions, M280 exhibits a large degree of flexibility, and the interaction with Y444 is broken (open state) as it cannot compensate the overall increased dynamics and the PDZ1-PDZ2, leading to trimerization of DegP. At low temperatures, the protease activity of DegP can be triggered by activating substrates, which mediate the formation of proteolytic (12/24-mer) cages via a transient trimerization through interactions with the PDZ1 domain. Upon temperature activation, the M280-Y444 lock mediates the transformation of DegP from the locked inactive hexameric state to an open trimeric state, primed for the proteolytic degradation of substrates, but still requiring activation through substrate binding. Under heat-shock conditions, the structural basis for cage formation remains unclear, although DegP is still able to form cages under these conditions (*23*).

Together, our backbone and side-chain dynamical data draw a detailed picture of how the temperature-dependent breakage of the interdomain lock relieves the fine-tuned inherent dynamics of PDZ1 and uses these to ensure efficient substrate interaction (Fig. 9). Whereas the lack of substantial backbone motions at 25°C directly explains the consequence of the locked conformation at low temperatures, possibly driven by a reduction of the local frustration in M280 bearing helix α_1_ in cellular ground states, the situation changes with increasing temperatures, where we begin to observe extensive inherent dynamics released upon breakage of the interdomain lock. The amplitude of motions at 37°C (Figs. 5A and 6D) reveals a fine-tuned dynamical compensation, which can easily adapt to slight changes in temperature to either lock the interaction (at a lower temperature) or unlock the interaction and consequently activate also the protease function of DegP (at a higher temperature). Whereas under heat-shock conditions, the extensive inherent dynamics on the micro- to millisecond time scale within PDZ1 indicate a domain optimized for substrate interaction, facilitating subsequent proteolytic cleavage by the protease domain. This finding poses the very interesting question whether and how the proposed large DegP cages can form at elevated temperatures. Therefore, under heat-shock conditions, the formation of the larger DegP states might not be supported by the stabilization of the PDZ1-PDZ2 lock, although cages are supposed to form between 25° and 48°C (*23*). Alternatively, upon cage formation, there could be an allosteric feedback loop from the protease domain back toward the PDZ1 domain, with the resulting compensation of the frustrated area around α_1_ once again forming the main driving force for a stable complex formation via the M280-Y444 domain lock (Fig. 9). The exact nature of the existence and/or the stabilization of the interdomain lock at elevated temperatures needs further research as most studies so far focused on lower temperatures (*12*, *15*, *17*, *23*).

On a broader scope, the involved C-terminal Y444 is found in several HtrA proteins known to exploit a similar locking mechanism (fig. S13, A to E) (*12*, *21*, *48*, *49*). Furthermore, the involved residues in DegP, M280, and Y444 are also highly conserved within different HtrA proteins carrying two PDZ domains, with the tyrosine showing a slightly higher conservation score than the methionine (fig. S13, F and G). Our finding might therefore be indicative of a more widespread mechanism not only restricted to HtrA proteins as it is already known that C-terminal aromatic/hydrophobic residues in a variety of mammalian multi-PDZ domain proteins govern the formation of homo- and heterodimers, suggesting a possible evolutionary conserved mechanism of stabilizing PDZ-PDZ interactions (*50*, *51*).

## MATERIALS AND METHODS

### Cloning of different DegP domain constructs

The pDS4 plasmid contains wild-type *degP* (*E. coli* K12 strain) lacking its N-terminal signaling sequence within a pET28b vector backbone, yielding DegP with an N-terminal His_6_-tag (purchased from GenScript). The individual PDZ domains, the PDZ1-PDZ2 construct, and the SUMO tag were amplified by polymerase chain reaction (PCR) from pDS4 and pET28_SUMO_Hsc70 [a gift of B. Bukau (Heidelberg)] (*52*) plasmids, respectively. The different DegP-PDZ constructs and the SUMO tag were fused by standard PCR methods and cloned into a pET28a(−) vector via the Nco I and Xho I restriction sites by standard cloning techniques yielding PDZ constructs fused to an N-terminal His_6_-SUMO tag. Plasmids and primers used in this study can be found in table S3.

### Protein expression and purification of PDZ1-PDZ2 domains

pDS10 (DegP^261–354^), pDS11 (DegP^359–448^), and pDS12 (DegP^261–448^) plasmids were chemically transformed into *E. coli* BL21 Star (λ DE3) cells. The transformed cells were grown at 37°C in 1 liter of LB medium containing kanamycin (50 μg/ml) until an optical density at 600 nm ≈ 0.5 was reached. Expression was induced by the addition of 0.4 mM isopropyl-β-d-thiogalactopyranoside, and the cells were left to grow overnight at 25°C. Cells were harvested by centrifugation at 8000*g* for 20 min at 4°C and subsequently resuspended in 25 ml of lysis buffer [25 mM Hepes/NaOH, 0.5 M NaCl, and 10 mM imidazole (pH 7.5)]. The resuspended cells were incubated on ice with one cOmplete EDTA-free protease inhibitor (Roche) tablet and with 100 U of deoxyribonuclease (ArcticZymes Technologies) for 30 min and subsequently lysed with a Q700 sonicator (Qsonica) (10 s on, 30 s off, 30% power). Cell debris was removed by centrifugation at 18,000*g* for 45 min at 4°C, and the supernatant was applied to a Ni^2+^ Sepharose 6 Fast Flow (GE Healthcare)–loaded gravity column, followed by stepwise elution with 20 ml of lysis buffer supplemented with 100 and 500 mM imidazole, respectively. Fractions containing the target proteins were dialyzed against phosphate-buffered saline (PBS) (pH 7.4), and the His_6_-SUMO tag was removed by enzymatic cleavage using human Sentrin-specific protease 1 (SENP1) protease at 4°C overnight (Addgene, no. 16356) (*53*). The cleaved proteins were applied again to a Ni^2+^ column, and the flow through was collected. The proteins were concentrated using Amicon centrifugal filters [5k Molecular weight cut-off (MWCO) or 10k MWCO, Millipore] and purified further by size exclusion chromatography (Superdex 75 Increase, GE Healthcare) in NMR buffer [25 mM potassium phosphate, 1 mM EDTA, and 1 mM tris(2-carboxyethyl)phosphine (TCEP) (pH 7.0)].

For His_6_-tagged full-length DegP variants, an additional unfolding/refolding step was introduced. To this end, after cell lysis and centrifugation, the supernatant was loaded to a HisTrap HP 5-ml column (GE Healthcare) equilibrated with lysis buffer supplemented with 6 M guanidinium chloride. For on-column refolding, the bound protein was washed with a 6 to 0 M guanidinium chloride linear gradient [15 column volume (CV), 0.5 ml/min] overnight and subsequently eluted with a step elution of 100 and 500 mM imidazole. In the next step, DegP was purified by size exclusion chromatography (Superdex 200 Increase, GE Healthcare) in NMR buffer.

### Isotope labeling

We obtained [U-^15^N]- and [U-^15^N,^13^C]-labeled PDZ domain constructs by growing the expression cells in M9 minimal medium (*54*), supplemented with (^15^NH_4_)Cl and D-(^13^C)-glucose, respectively. For full-length DegP, the cells were in addition grown in D_2_O-based M9 minimal medium, yielding [U-^2^H,^15^N] and upon the usage of D-(^13^C,^2^H)-glucose [U-^2^H,^15^N,^13^C] proteins, respectively. For specific methyl group labeling, we either supplemented D_2_O-based M9 minimal medium with D-(^12^C,^2^H)-glucose and isoleucine precursor (2-ketobutyric acid-4-^13^C,3,3-d_2_ sodium salt hydrate) 1 hour before induction resulting in [U-^2^H, Ile-δ_1_-^13^CH_3_] or in a similar manner with D-(^12^C,^2^H)-glucose and leucine, valine precursor [2-keto-3-methyl-d_3_-3-d_1_-4-^13^C-butyrate], resulting in nonstereospecific [U-^2^H, Leu/Val-^13^CH_3_/^12^CD_2_]-labeled DegP variants. All isotopes were purchased from Sigma-Aldrich/Merck.

### NMR spectroscopy

NMR measurements were performed on Bruker Avance III HD 700- or 800-MHz spectrometers, running TopSpin 3.5/3.6 and equipped with a cryogenically cooled triple-resonance probe. All experiments were performed in NMR buffer [25 mM potassium phosphate, 1 mM EDTA, and 1 mM TCEP (pH 7.0)].

For the sequence-specific backbone resonance assignment experiments of DegP^S210A^ constructs and the different PDZ domain constructs, the following experiments were recorded: 2D [^15^N,^1^H]-TROSY-HSQC (*55*), and for full-length DegP^S210A,Y444A^, the following TROSY-type 3D experiments: 3D trHNCA, 3D trHNCACB, and 3D trHNCO (*56*), whereas, for the different PDZ domain constructs, standard through-bond 3D HNCA, 3D HNCACB, 3D HNCO, and 3D CBCA(CO)NH (*57*) experiments were used. Aliphatic side-chain resonance assignment for the PDZ1-PDZ2 construct was performed on the basis of 2D [^13^C,^1^H]-HSQC spectra with/without constant time (CT) version, as well as 3D (H)CC(CO)NH, H(CC)(CO)NH, and HCCH-TOCSY experiments (*57*). In addition, the following NOESY-type experiments with the indicated mixing times were performed: 3D H_all_-H_aro_-C_aro_ with 120-ms mixing time, 3D H_all_-H_ali_-C_ali_ with 120-ms mixing time (*57*), and 3D ^13^C_m_-^13^C_m_^1^H_m_ SOFAST NOESY with 50-ms mixing time (*58*). Assignment experiments were performed at 50°C if not indicated otherwise.

For the quantitative analysis of signal intensities, the amplitudes were corrected by differences in the ^1^H-90° pulse length, the number of scans, and the dilution factor (*59*). Furthermore, for comparison of spectra at different temperatures, spectra were scaled to compensate for the changes in transversal relaxation rates, *R*_2_, leading to about 70% signal intensity at 25°C compared to the reference datasets at 50°C. NMR data were processed with a combination of NMRPipe (*60*) and mddNMR2.6 (*61*) and analyzed with CARA (*62*).

For titration experiments, 2D [^15^N,^1^H]-TROSY-HSQC experiments were acquired with four scans and 2048 × 256 complex points in the direct and indirect dimensions, respectively. The chemical shift changes of the amide moiety were calculated as follows

$$∆\delta (\text{HN})=\sqrt{{\left(∆\delta^{1}H\right)}^{2}+{\left(∆\delta^{15}N/5\right)}^{2}}$$ (1)

The chemical shift changes of the methyl groups were calculated as follows

$$∆\delta (\text{HC})=\sqrt{{\left(∆\delta^{1}H\right)}^{2}+{\left(∆\delta^{13}C/4\right)}^{2}}$$ (2)

Secondary chemical shifts were calculated relative to the random coil values using the prediction software POTENCI (*63*). Furthermore, a weighting function with weights 1-2-1 for residues (*i − 1*)–*i*–(*i + 1*) was applied to the raw data (*5*, *37*).

### NMR backbone dynamics

For the analysis of the dynamic properties of the different DegP constructs, the following relaxation experiments were measured: ^15^N{^1^H}-NOE (*64*), *T*_1_(^15^N) (*64*), *T*_1ρ_(^15^N) (*65*), and TROSY for rotational correlation times (TRACT) for determining *T*_2α_(^15^N) and *T*_2β_(^15^N) (*24*) at 700-MHz (16.4 T) proton (^1^H) frequency. Nonlinear least square fits of relaxation data were done with MATLAB (MathWorks) and the Dynamics Center 2.5 (Bruker Biospin). *R*_2(R1ρ)_(^15^N) values were derived from *T*_1ρ_ (*66*) using Eq. 3

$$R_{2}=\frac{R_{1\rho }}{{\text{sin}}^{2}\theta }-\frac{R_{1}}{{\text{tan}}^{2}\theta }$$ (3)

with θ=tan−1(ωΩ), where ω is the spin-lock field strength (2 kHz) and Ω is the offset from the ^15^N carrier frequency.

Error bars for *R*_1_(^15^N), *R*_1ρ_(^15^N), *R*_2α_(^15^N), and *R*_2β_(^15^N) were calculated by a Monte Carlo simulation embedded within Dynamics Center 2.5 (Bruker, Biospin) and for *R*_2(R1ρ)_ (^15^N) by error propagation. Error bars for the ^15^N{^1^H}-NOE were calculated from the spectral noise. Analysis of the obtained relaxation rates was performed with Tensor2 (*67*) using an anisotropic diffusion tensor on the NMRbox web server (*68*). Missing atoms and hydrogens in the Protein Data Bank (PDB) structure (3OU0) were added using PDBFixer (*69*). Temperature-dependent rotation correlation times (τ_c_) were estimated by using HYDRONMR version 7c (*70*) via NMRBox at 700-MHz (16.4 T) magnetic field strength using a standard atomic element radius value of 3.3 Å, a 1.02-Å N─H bond length, a Chemical Shift Anisotropy (CSA) of −160 ppm, with the refined PDZ1-PDZ2 crystal structure (PDB ID: 3OU0). For the temperature-dependent changes in viscosity of the water, the standard values were obtained from the National Institute of Standards and Technology website (https://webbook.nist.gov/chemistry/fluid/).

### NMR side-chain dynamics

Experiments were performed on either a [U-^2^H, Ile-δ_1_-^13^CH_3_]-PDZ1-PDZ2 or a [U-^2^H, Leu-δ_1,2_-Val- γ_1,2_^13^CH_3_]-PDZ1-PDZ2 sample at a temperature of 37°C in 99.9% D_2_O-based NMR buffer. Side-chain methyl order parameters (S^2^_axis_) were determined by cross-correlated relaxation experiments (*71*, *72*). Single-quantum and triple-quantum ^1^H-^13^C experiments were collected at a series of delay times. Ratios of the peak intensities were fitted for eight values ranging between 2 and 24 ms using the following equation, where *T* is the relaxation delay time and δ is a factor to account for coupling due to relaxation with external protons

$$|\frac{I_{a}}{I_{b}}|=\frac{3}{4}\frac{\eta \;\text{tan}\;h(\sqrt{\eta^{2}+\delta^{2}}T)}{\sqrt{\eta^{2}+\delta^{2}-\delta \;\text{tan}\;h(\sqrt{\eta^{2}+\delta^{2}}T)}}$$ (4)

S^2^_axis_ values were determined using Eq. 5, using their fitted η values and the separately determined rotational correlation time adjusted for the change in viscosity in 100% D_2_O (τ_c_) of 14.4 ns at 37°C

$$\eta \approx \frac{9}{10}\;(\frac{\mu_{0}}{4\pi }){\left[P_{2}(\text{cos}\Theta_{\text{axis},\text{HH}})\right]}^{2}\frac{S_{\text{axis}}^{2}\gamma_{H}^{4}\hbar^{2}\tau_{c}}{r_{\text{HH}}^{6}}$$ (5)

where μ_0_ is the vacuum permittivity constant, γ_H_ the gyromagnetic ratio of the proton spin, *r*_HH_ is the distance between pairs of methyl protons (1.813 Å), S^2^_axis_ is the generalized order parameter describing the amplitude of motion of the methyl threefold axis, Θ_axis,HH_ is the angle between the methyl symmetry axis and a vector between a pair of methyl protons (90°), and *P*_2_(*x*) = ½ (3*x*^2^ – 1). Last, the product of the methyl order parameter and the overall correlation time constant, S^2^_axis_·τ_C_, was determined.

MQ methyl relaxation dispersion experiments (*40*) were recorded as a series of 2D datasets using CT relaxation periods (*T*) of 30 ms [800 MHz (18.8 T); 25°, 37°, 43°, and 50°C) and CPMG frequencies ranging from 67 to 1000 Hz. *R*_2,eff_, the effective transverse relaxation rate, was calculated according to the following equation

$$R_{2,\text{eff}}=-(\frac{1}{T})\text{ln}(\frac{I}{I_{0}})$$ (6)

where *I* (or *I*_0_) are the intensities with and without the presence of a constant time relaxation interval of duration *T*, during which a variable number of ^13^C 180° pulses are applied leading to ν_CPMG_ = 1/(2δ), where δ is the time between successive pulses. Dispersion data were fitted numerically to a two-site exchange model using the program ChemEx (available at https://github.com/gbouvignies/chemex/releases).

### NMR magnetization exchange

2D methyl magnetization exchange experiments were recorded to quantify the exchange between the locked (l) and open (o) state of the Met280-^13^Cε^1^Hε methyl group as outlined by Farrow *et al.* (*73*). The ^13^C-EXSY-SOFAST-HMQC experiments (*30*) were recorded with varying delays between 50 and 1000 ms at 37°C. To ensure equilibration of the initial magnetization, a long relaxation delay (1 s) was chosen. The signal intensities of the observed three peaks of M280-^13^Cε^1^Hε—namely, the autopeak of the locked conformation (*I*_ll_), the autopeak of the open conformation (*I*_oo_), and the cross peak originating from the transition from the locked to the open state, (*I*_lo_)—were determined. Because of signal overlap the second cross peak, *I*_ol_, could not be determined from the spectra; therefore, it was assumed in a first-order approximation that it follows a similar buildup than *I*_lo_. All data points were simultaneously fitted to the following theoretical equations

$$I_{\text{ll}}(T)=\frac{I_{l}(0)\{-(\lambda_{2}-x_{l})e^{-\lambda_{1}T}+(\lambda_{1}-x_{l})e^{-\lambda_{2}T}\}}{\lambda_{1}-\lambda_{2}}$$

$$I_{\text{oo}}(T)=\frac{I_{o}(0)\{-(\lambda_{2}-x_{o})e^{-\lambda_{1}T}+(\lambda_{1}-x_{o})e^{-\lambda_{2}T}\}}{\lambda_{1}-\lambda_{2}}$$

$$I_{\text{lo}}(T)=\frac{-I_{l}(0)k_{\text{lo}}(e^{-\lambda_{1}T}-e^{-\lambda_{2}T})}{\lambda_{1}-\lambda_{2}}$$

$$I_{\text{ol}}(T)=\frac{-I_{o}(0)k_{\text{ol}}(e^{-\lambda_{1}T}-e^{-\lambda_{2}T})}{\lambda_{1}-\lambda_{2}}$$ (7)

where λ_1,2_, *x*_l_, and *x*_o_ are defined according to the following relationships

$$\lambda_{1,2}=\frac{(x_{l}+x_{o})\pm \sqrt{{\left(x_{l}-x_{\text{lo}}\right)}^{2}+4k_{\text{lo}}k_{\text{ol}}}}{2}$$

$$x_{l}=R_{1l}+k_{\text{lo}}$$

$$x_{o}=R_{1o}+k_{\text{ol}}$$ (8)

and the rate of transition from the locked to the open conformations (*k*_lo_), the rate of transition vice versa (*k*_ol_), the longitudinal relaxation rate in the locked conformation (*R*_1l_), and the longitudinal relaxation rate of the open conformation (*R*_1o_), were fitted. *I*_l_(0) and *I*_o_(0) denote the initial amounts of longitudinal carbon magnetization associated with the locked and the open conformations at the beginning of the mixing periods. The S.D. of the determined exchange rate (*k*_ex_) was estimated on the basis of the spectral noise using a Monte Carlo approach.

### NMR diffusion

Translational diffusion coefficients were measured by recording a series of 1D ^13^C-edited spectra at different temperatures, using a pulse scheme [^13^C-edited bipolar-gradient-pulse pair longitudinal eddy current delay (BPP-LED)] that is similar to a ^15^N-edited BPP-LED experiment with ^15^N and ^13^C pulses interchanged (*25*). To compensate for the different *J*-coupling of the methyl groups compared to the amide groups, a gradient duration *d* of 3.2 ms was used instead of 4.8 ms in the ^15^N-filtered version. In addition, a diffusion delay *T* of 400 ms and a τ of 0.1 ms were used. The strength of the encoding/decoding was increased stepwise. Transverse gradients instead of *z* gradients were used to minimize convection effects (*74*). The resulting ^1^H signal was integrated over the methyl ^1^H frequency range to obtain intensities as a function of encoding/decoding gradient strength. To adjust for the temperature-dependent changes in viscosity of the buffer, the obtained signal intensity was adjusted on the basis of published temperature-dependent viscosity values for D_2_O-based buffers over the used temperature range (*75*). To construct the calibration curve given in fig. S1J, the following published translational diffusion coefficients, measured at 25°C, were used: immunoglobin-binding domain of streptococcal protein G, GB1 (MW = 6.2 kDa) (*76*), lysozyme (MW = 14.1 kDa) and interleukin-10 (MW = 37.2 kDa) (*77*), bacterial HsIV (MW = 230 kDa), one-half proteasome from *Thermoplasma acidophilum*, α7α7 (MW = 360 kDa), and the α7 single ring variant of the proteasome (MW = 180 kDa) (*78*).

### Biolayer interferometry

BLI experiments were performed on an Octet RED96 system (FortéBio) at 25°C as outlined in detail before (*79*). Briefly, the respective ligands were biotinylated using the biotinylation kit EZ-Link NHS-PEG4-Biotin (Thermo Fisher Scientific). The biotin label was resolved in H_2_O and directly added to the protein solution in a final molar ratio of 1:1 in 25 mM MES (pH 6.0) to ensure preferential labeling of the N-terminal α-amino group, followed by gentle mixing at room temperature for 45 min. Unreacted biotin was removed with Zeba Spin Desalting Columns (7 MWCO, Thermo Fisher Scientific). Biotin-labeled proteins were immobilized on the streptavidin biosensors (FortéBio), and the biosensors were subsequently blocked with EZ-Link Biocytin (Thermo Fisher Scientific). Analytes were diluted and applied in a dose-dependent manner to the biosensors immobilized with the biotinylated ligand. Bovine serum albumin powder (Sigma-Aldrich) and Tween 20 were added to final concentrations of 0.1 and 0.05%, respectively, to avoid nonspecific interactions. Parallel experiments were performed for reference sensors with no analyte bound, and the signals were subsequently subtracted during data analysis. The association period was set to 30 s and dissociation to 40 s. Data measurements and analysis were performed by using the Data Acquisition 10.0 and the Data Analysis HT 10.0 (FortéBio) software, respectively.

### Proteolytic cleavage assay

The fluorescent nonactivating reporter peptide (Abz-KASPVSLGY^NO2^D) and p23 (Abz-GNWVSAAKFETYR^NO3^SKNTQDYGILQI), originally described in (*45*) and (*23*), respectively, were purchased from GenScript and prepared by dissolving the lyophilized peptides directly in dimethyl sulfoxide (p23) or PBS (Abz-KASPVSLGY^NO2^D). Cleavage assays were monitored using a FLUOstar Optima microplate reader (BMG LABTECH) using an excitation wavelength of 320 nm and an emission wavelength of 440 nm, respectively, in 96-well black clear bottom microplates (Corning). All experiments were prepared in PBS and contained either 0.2 μM of the respective DegP enzyme (monomer concentration) and 7.5 μM p23 peptide or 10 μM enzyme with 50 μM β-casein (Sigma-Aldrich) and 100 μM Abz-KASPVSLGY^NO2^D peptide. All assays were performed as triplicates.

β-casein degradation was, in addition, assessed via SDS-PAGE by incubating 1 μM DegP with 5 μM β-casein in PBS at 25° or 37°C at specific time intervals of 0, 5, 10, 20, 30, 60, and 90 min, respectively. Samples were taken out, mixed with sample loading dye, and subsequently heat-denatured at 95°C for 10 min, before loading them onto SDS-PAGE gels. The samples were subsequently analyzed by SDS-PAGE using 4 to 20% Mini-PROTEAN TGX–SDS Gels (Bio-Rad).

## Supplementary Materials

### This PDF file includes:

Figs. S1 to S13 Tables S1 to S3 Supplementary Note References

## Notes

View/request a protocol for this paper from *Bio-protocol*.

## References

[1] F. U. Hartl, A. Bracher, M. Hayer-Hartl, Molecular chaperones in protein folding and proteostasis. Nature 475, 324–332 (2011). 21776078 10.1038/nature10317

[2] D. Balchin, M. Hayer-Hartl, F. U. Hartl, *In vivo* aspects of protein folding and quality control. Science 353, aac4354 (2016). 27365453 10.1126/science.aac4354

[3] J. G. Sklar, T. Wu, D. Kahne, T. J. Silhavy, Defining the roles of the periplasmic chaperones SurA, Skp, and DegP in *Escherichia coli*. Genes Dev. 21, 2473–2484 (2007). 17908933 10.1101/gad.1581007 PMC1993877

[4] J. Thoma, B. M. Burmann, S. Hiller, D. J. Müller, Impact of holdase chaperones Skp and SurA on the folding of β-barrel outer-membrane proteins. Nat. Struct. Mol. Biol. 22, 795–802 (2015). 26344570 10.1038/nsmb.3087

[5] B. M. Burmann, C. Wang, S. Hiller, Conformation and dynamics of the periplasmic membrane-protein–chaperone complexes OmpX–Skp and tOmpA–Skp. Nat. Struct. Mol. Biol. 20, 1265–1272 (2013). 24077225 10.1038/nsmb.2677

[6] O. Subrini, J. M. Betton, Assemblies of DegP underlie its dual chaperone and protease function. FEMS Microbiol. Lett. 296, 143–148 (2009). 19508278 10.1111/j.1574-6968.2009.01658.x

[7] J. Skórko-Glonek, A. Wawrzynów, K. Krzewski, K. Kurpierz, B. Lipińska, Site-directed mutagenesis of the HtrA (DegP) serine-protease, whose proteolytic activity is indispensable for *Escherichia coli* survival at elevated-temperatures. Gene 163, 47–52 (1995). 7557477 10.1016/0378-1119(95)00406-v

[8] R. Misra, M. CastilloKeller, M. Deng, Overexpression of protease-deficient DegP(S210A) rescues the lethal phenotype of *Escherichia coli* OmpF assembly mutants in a *degP* background. J. Bacteriol. 182, 4882–4888 (2000). 10940032 10.1128/jb.182.17.4882-4888.2000 PMC111368

[9] M. CastilloKeller, R. Misra, Protease-deficient DegP suppresses lethal effects of a mutant OmpC protein by its capture. J. Bacteriol. 185, 148–154 (2003). 12486051 10.1128/JB.185.1.148-154.2003 PMC141919

[10] M. B. Kennedy, Origin of PDZ (DHR, GLGF) domains. Trends Biochem. Sci. 20, 350 (1995). 7482701 10.1016/s0968-0004(00)89074-x

[11] T. Clausen, M. Kaiser, R. Huber, M. Ehrmann, HTRA proteases: Regulated proteolysis in protein quality control. Nat. Rev. Mol. Cell Biol. 12, 152–162 (2011). 21326199 10.1038/nrm3065

[12] T. Krojer, J. Sawa, E. Schäfer, H. R. Saibil, M. Ehrmann, T. Clausen, Structural basis for the regulated protease and chaperone function of DegP. Nature 453, 885–890 (2008). 18496527 10.1038/nature07004

[13] T. Krojer, M. Garrido-Franco, R. Huber, M. Ehrmann, T. Clausen, Crystal structure of DegP (HtrA) reveals a new protease-chaperone machine. Nature 416, 455–459 (2002). 11919638 10.1038/416455a

[14] S. Kim, R. A. Grant, R. T. Sauer, Covalent linkage of distinct substrate degrons controls assembly and disassembly of DegP proteolytic cages. Cell 145, 67–78 (2011). 21458668 10.1016/j.cell.2011.02.024 PMC3075617

[15] N. J. Thompson, M. Merdanovic, M. Ehrmann, E. Van Duijn, A. J. R. Heck, Substrate occupancy at the onset of oligomeric transitions of DegP. Structure 22, 281–290 (2014). 24373769 10.1016/j.str.2013.11.010

[16] S. Li, R. Wang, D. Li, J. Ma, H. Li, X. He, Z. Chang, Y. Weng, Thermal-triggerd proteinquake leads to disassembly of DegP hexamer as an imperative activation step. Sci. Rep. 4, 4834 (2015). 10.1038/srep04834 PMC4003476 24776652

[17] J. Jiang, X. Zhang, Y. Chen, Y. Wu, Z. H. Zhou, Z. Chang, S.-F. Sui, Activation of DegP chaperone-protease *via* formation of large cage-like oligomers upon binding to substrate proteins. Proc. Natl. Acad. Sci. U.S.A. 105, 11939–11944 (2008). 18697939 10.1073/pnas.0805464105 PMC2575304

[18] D. Figaj, A. Gieldon, A. Polit, A. Sobiecka-Szkatula, T. Koper, M. Denkiewicz, B. Banecki, A. Lesner, J. Ciarkowski, B. Lipinska, J. Skorko-Glonek, The LA loop as an important regulatory element of the HtrA (DegP) protease from *Escherichia coli* structural and functional studies. J. Biol. Chem. 289, 15880–15893 (2014). 24737328 10.1074/jbc.M113.532895 PMC4140941

[19] T. Krojer, J. Sawa, R. Huber, T. Clausen, HtrA proteases have a conserved activation mechanism that can be triggered by distinct molecular cues. Nat. Struct. Mol. Biol. 17, 844–852 (2010). 20581825 10.1038/nsmb.1840

[20] S. Kim, I. Song, G. Eom, S. Kim, A small periplasmic protein with a hydrophobic C-terminal residue enhances DegP proteolysis as a suicide activator. J. Bacteriol. 200, e00519-17 (2018). 10.1128/JB.00519-17 PMC5763042 28947671

[21] Y. Toyama, R. W. Harkness, T. Y. T. Lee, J. T. Maynes, L. E. Kay, Oligomeric assembly regulating mitochondrial HtrA2 function as examined by methyl-TROSY NMR. Proc. Natl. Acad. Sci. 118, e2025022118 (2021). 33692127 10.1073/pnas.2025022118 PMC7980377

[22] H. Malet, F. Canellas, J. Sawa, J. Yan, K. Thalassinos, M. Ehrmann, T. Clausen, H. R. Saibil, Newly folded substrates inside the molecular cage of the HtrA chaperone DegQ. Nat. Struct. Mol. Biol. 19, 152–157 (2012). 22245966 10.1038/nsmb.2210 PMC3272482

[23] S. Kim, R. T. Sauer, Cage assembly of DegP protease is not required for substrate-dependent regulation of proteolytic activity or high-temperature cell survival. Proc. Natl. Acad. Sci. U.S.A. 109, 7263–7268 (2012). 22529381 10.1073/pnas.1204791109 PMC3358883

[24] D. Lee, C. Hilty, G. Wider, K. Wüthrich, Effective rotational correlation times of proteins from NMR relaxation interference. J. Magn. Reson. 178, 72–76 (2006). 16188473 10.1016/j.jmr.2005.08.014

[25] J. J. Chou, J. L. Baber, A. Bax, Characterization of phospholipid mixed micelles by translational diffusion. J. Biomol. NMR 29, 299–308 (2004). 15213428 10.1023/B:JNMR.0000032560.43738.6a

[26] K. Wüthrich, NMR assignments as a basis for structural characterization of denatured states of globular proteins. Curr. Opin. Struct. Biol. 4, 93–99 (1994).

[27] H. M. McConnell, Reaction rates by nuclear magnetic resonance. J. Chem. Phys. 28, 430–431 (1958).

[28] C. C. Valley, A. Cembran, J. D. Perlmutter, A. K. Lewis, N. P. Labello, J. Gao, J. N. Sachs, The methionine-aromatic motif plays a unique role in stabilizing protein structure. J. Biol. Chem. 287, 34979–34991 (2012). 22859300 10.1074/jbc.M112.374504 PMC3471747

[29] M. J. Plevin, D. L. Bryce, J. Boisbouvier, Direct detection of CH/π interactions in proteins. Nat. Chem. 2, 466–471 (2010). 20489715 10.1038/nchem.650

[30] G. Mas, J.-Y. Guan, E. Crublet, E. C. Debled, C. Moriscot, P. Gans, G. Schoehn, P. Macek, P. Schanda, J. Boisbouvier, Structural investigation of a chaperonin in action reveals how nucleotide binding regulates the functional cycle. Sci. Adv. 4, eaau4196 (2018). 30255156 10.1126/sciadv.aau4196 PMC6154984

[31] A. G. Palmer III, C. D. Kroenke, J. P. Loria, Nuclear magnetic resonance methods for quantifying microsecond-to-millisecond motions in biological macromolecules. Methods Enzymol. 339, 204–238 (2001). 11462813 10.1016/s0076-6879(01)39315-1

[32] N.-A. Lakomek, J. Ying, A. Bax, Measurement of ^15^N relaxation rates in perdeuterated proteins by TROSY-based methods. J. Biomol. NMR 53, 209–221 (2012). 22689066 10.1007/s10858-012-9626-5 PMC3412688

[33] B. M. Burmann, U. Scheckenhofer, K. Schweimer, P. Rösch, Domain interactions of the transcription-translation coupling factor *Escherichia coli* NusG are intermolecular and transient. Biochem. J. 435, 783–789 (2011). 21345171 10.1042/BJ20101679

[34] B. M. Burmann, S. H. Knauer, A. Sevostyanova, K. Schweimer, R. A. Mooney, R. Landick, I. Artsimovitch, P. Rösch, An α helix to β barrel domain switch transforms the transcription factor RfaH into a translation factor. Cell 150, 291–303 (2012). 22817892 10.1016/j.cell.2012.05.042 PMC3430373

[35] G. Lipari, A. Szabo, Model-Free approach to the interpretation of nuclear magnetic resonance relaxation in macromolecules. 2. Analysis of experimental results. J. Am. Chem. Soc. 104, 4559–4570 (1982).

[36] G. M. Clore, A. Szabo, A. Bax, L. E. Kay, P. C. Driscoll, A. M. Gronenborn, Deviations from the simple two-parameter model-free approach to the interpretation of nitrogen-15 nuclear magnetic relaxation of proteins. J. Am. Chem. Soc. 112, 4989–4991 (1990).

[37] L. Morgado, B. M. Burmann, T. Sharpe, A. Mazur, S. Hiller, The dynamic dimer structure of the chaperone Trigger Factor. Nat. Commun. 8, 1992 (2017). 29222465 10.1038/s41467-017-02196-7 PMC5722895

[38] L. He, T. Sharpe, A. Mazur, S. Hiller, A molecular mechanism of chaperone-client recognition. Sci. Adv. 2, e1601625 (2016). 28138538 10.1126/sciadv.1601625 PMC5262456

[39] R. G. Parra, N. P. Schafer, L. G. Radusky, M. Y. Tsai, A. B. Guzovsky, P. G. Wolynes, D. U. Ferreiro, Protein Frustratometer 2: A tool to localize energetic frustration in protein molecules, now with electrostatics. Nucleic Acids Res. 44, W356–W360 (2016). 27131359 10.1093/nar/gkw304 PMC4987889

[40] D. M. Korzhnev, K. Kloiber, V. Kanelis, V. Tugarinov, L. E. Kay, Probing slow dynamics in high molecular weight proteins by methyl-TROSY NMR spectroscopy: Application to a 723-residue enzyme. J. Am. Chem. Soc. 126, 3964–3973 (2004). 15038751 10.1021/ja039587i

[41] A. K. Lewis, K. M. Dunleavy, T. L. Senkow, C. Her, B. T. Horn, M. A. Jersett, R. Mahling, M. R. McCarthy, G. T. Perell, C. C. Valley, C. B. Karim, J. Gao, W. C. K. Pomerantz, D. D. Thomas, A. Cembran, A. Hinderliter, J. N. Sachs, Oxidation increases the strength of the methionine-aromatic interaction. Nat. Chem. Biol. 12, 860–866 (2016). 27547920 10.1038/nchembio.2159 PMC5060120

[42] A. Fujii, H. Hayashi, J. W. Park, T. Kazama, N. Mikami, S. Tsuzuki, Experimental and theoretical determination of the accurate CH/π interaction energies in benzene-alkane clusters: Correlation between interaction energy and polarizability. Phys. Chem. Chem. Phys. 13, 14131–14141 (2011). 21566845 10.1039/c1cp20203k

[43] R. Sprangers, A. Gribun, P. M. Hwang, W. A. Houry, L. E. Kay, Quantitative NMR spectroscopy of supramolecular complexes: Dynamic side pores in ClpP are important for product release. Proc. Natl. Acad. Sci. U.S.A. 102, 16678–16683 (2005). 16263929 10.1073/pnas.0507370102 PMC1283831

[44] S. Vahidi, Z. A. Ripstein, J. B. Juravsky, E. Rennella, A. L. Goldberg, A. K. Mittermaier, J. L. Rubinstein, L. E. Kay, An allosteric switch regulates *Mycobacterium tuberculosis ClpP1P2* protease function as established by cryo-EM and methyl-TROSY NMR. Proc. Natl. Acad. Sci. U.S.A. 117, 5895–5906 (2020). 32123115 10.1073/pnas.1921630117 PMC7084164

[45] M. E. Lee, T. A. Baker, R. T. Sauer, Control of substrate gating and translocation into ClpP by channel residues and ClpX binding. J. Mol. Biol. 399, 707–718 (2010). 20416323 10.1016/j.jmb.2010.04.027 PMC2885556

[46] L. Truebestein, A. Tennstaedt, T. Mönig, T. Krojer, F. Canellas, M. Kaiser, T. Clausen, M. Ehrmann, Substrate-induced remodeling of the active site regulates human HTRA1 activity. Nat. Struct. Mol. Biol. 18, 386–388 (2011). 21297635 10.1038/nsmb.2013

[47] R. V. Mauldin, R. T. Sauer, Allosteric regulation of DegS protease subunits through a shared energy landscape. Nat. Chem. Biol. 9, 90–96 (2013). 23201899 10.1038/nchembio.1135 PMC3551985

[48] C. Wilken, K. Kitzing, R. Kurzbauer, M. Ehrmann, T. Clausen, Crystal structure of the DegS stress sensor. Cell 117, 483–494 (2004). 15137941 10.1016/s0092-8674(04)00454-4

[49] J. Sawa, H. Malet, T. Krojer, F. Canellas, M. Ehrmann, T. Clausen, Molecular adaptation of the DegQ protease to exert protein quality control in the bacterial cell envelope. J. Biol. Chem. 286, 30680–30690 (2011). 21685389 10.1074/jbc.M111.243832 PMC3162429

[50] B. J. Hillier, K. S. Christopherson, K. E. Prehoda, D. S. Bredt, W. A. Lim, Unexpected modes of PDZ domain scaffolding revealed by structure of nNOS-syntrophin complex. Science 284, 812–815 (1999). 10221915

[51] S. Srivastava, P. Osten, F. S. Vilim, L. Khatri, G. Inman, B. States, C. Daly, S. DeSouza, R. Abagyan, J. G. Valtschanoff, R. J. Weinberg, E. B. Ziff, Novel anchorage of GluR2/3 to the postsynaptic density by the AMPA receptor-binding protein ABP. Neuron 21, 581–591 (1998). 9768844 10.1016/s0896-6273(00)80568-1

[52] R. Schlecht, S. R. Scholz, H. Dahmen, A. Wegener, C. Sirrenberg, D. Musil, J. Bomke, H. M. Eggenweiler, M. P. Mayer, B. Bukau, Functional analysis of Hsp70 inhibitors. PLOS ONE 8, e78443 (2013). 24265689 10.1371/journal.pone.0078443 PMC3827032

[53] J. Mikolajczyk, M. Drag, M. Békés, J. T. Cao, Z. Ronai, G. S. Salvesen, Small Ubiquitin-related Modifier (SUMO)-specific proteases: Profiling the specificities and activities of human SENPs. J. Biol. Chem. 282, 26217–26224 (2007). 17591783 10.1074/jbc.M702444200

[54] J. Sambrook, E. F. Fritsch, T. Maniatis, *Molecular Cloning: A Laboratory Manual* (Cold Spring Harbor Laboratory, 1989), vol. 2.

[55] K. Pervushin, R. Riek, G. Wider, K. Wüthrich, Attenuated T_2_ relaxation by mutual cancellation of dipole-dipole coupling and chemical shift anisotropy indicates an avenue to NMR structures of very large biological macromolecules in solution. Proc. Natl. Acad. Sci. U.S.A. 94, 12366–12371 (1997). 9356455 10.1073/pnas.94.23.12366 PMC24947

[56] M. Salzmann, K. Pervushin, G. Wider, H. Senn, K. Wüthrich, TROSY in triple-resonance experiments: New perspectives for sequential NMR assignment of large proteins. Proc. Natl. Acad. Sci. U.S.A. 95, 13585–13590 (1998). 9811843 10.1073/pnas.95.23.13585 PMC24862

[57] M. Sattler, J. Schleucher, C. Griesinger, Heteronuclear multidimensional NMR experiments for the structure determination of proteins in solution employing pulsed field gradients. Prog. Nucl. Magn. Reson. Spectrosc. 34, 93–158 (1999).

[58] P. Rossi, Y. Xia, N. Khanra, G. Veglia, C. G. Kalodimos, ^15^N and ^13^C- SOFAST-HMQC editing enhances 3D-NOESY sensitivity in highly deuterated, selectively [^1^H,^13^C]-labeled proteins. J. Biomol. NMR 66, 259–271 (2016). 27878649 10.1007/s10858-016-0074-5 PMC5218894

[59] G. Wider, L. Dreier, Measuring protein concentrations by NMR spectroscopy. J. Am. Chem. Soc. 128, 2571–2576 (2006). 16492040 10.1021/ja055336t

[60] F. Delaglio, S. Grzesiek, G. W. Vuister, G. Zhu, J. Pfeifer, A. Bax, NMRPipe: A multidimensional spectral processing system based on UNIX pipes. J. Biomol. NMR 6, 277–293 (1995). 8520220 10.1007/BF00197809

[61] V. Jaravine, I. Ibraghimov, V. Y. Orekhov, Removal of a time barrier for high-resolution multidimensional NMR spectroscopy. Nat. Methods 3, 605–607 (2006). 16862134 10.1038/nmeth900

[62] R. L. J. Keller, *The Computer Aided Resonance Assignment Tutorial* (Cantina Verlag, Goldau, 2004).

[63] J. T. Nielsen, F. A. A. Mulder, Potenci: Prediction of temperature, neighbor and pH-corrected chemical shifts for intrinsically disordered proteins. J. Biomol. NMR 70, 141–165 (2018). 29399725 10.1007/s10858-018-0166-5

[64] G. Zhu, Y. Xia, L. K. Nicholson, K. H. Sze, Protein dynamics measurements by TROSY-based NMR experiments. J. Magn. Reson. 143, 423–426 (2000). 10729271 10.1006/jmre.2000.2022

[65] T. Szyperski, P. Lunginbühl, G. Otting, P. Güntert, K. Wüthrich, Protein dynamics studied by rotating frame ^15^N spin relaxation-times. J. Biomol. NMR 3, 151–164 (1993). 7682879 10.1007/BF00178259

[66] T. A. Walton, C. M. Sandoval, C. A. Fowler, A. Pardi, M. C. Sousa, The cavity-chaperone Skp protects its substrate from aggregation but allows independent folding of substrate domains. Proc. Natl. Acad. Sci. U.S.A. 106, 1772–1777 (2009). 19181847 10.1073/pnas.0809275106 PMC2644113

[67] P. Dosset, J. C. Hus, M. Blackledge, D. Marion, Efficient analysis of macromolecular rotational diffusion from heteronuclear relaxation data. J. Biomol. NMR 16, 23–28 (2000). 10718609 10.1023/a:1008305808620

[68] M. W. Maciejewski, A. D. Schuyler, M. R. Gryk, I. I. Moraru, P. R. Romero, E. L. Ulrich, H. R. Eghbalnia, M. Livny, F. Delaglio, J. C. Hoch, NMRbox: A Resource for biomolecular NMR computation. Biophys. J. 112, 1529–1534 (2017). 28445744 10.1016/j.bpj.2017.03.011 PMC5406371

[69] P. Eastman, M. S. Friedrichs, J. D. Chodera, R. J. Radmer, C. M. Bruns, J. P. Ku, K. A. Beauchamp, T. J. Lane, L. P. Wang, D. Shukla, T. Tye, M. Houston, T. Stich, C. Klein, M. R. Shirts, V. S. Pande, OpenMM 4: A reusable, extensible, hardware independent library for high performance molecular simulation. J. Chem. Theory Comput. 9, 461–469 (2013). 23316124 10.1021/ct300857j PMC3539733

[70] J. García De La Torre, M. L. Huertas, B. Carrasco, Calculation of hydrodynamic properties of globular proteins from their atomic-level structure. Biophys. J. 78, 719–730 (2000). 10653785 10.1016/S0006-3495(00)76630-6 PMC1300675

[71] H. Sun, L. E. Kay, V. Tugarinov, An optimized relaxation-based coherence transfer NMR experiment for the measurement of side-chain order in methyl-protonated, highly deuterated proteins. J. Phys. Chem. B 115, 14878–14884 (2011). 22040035 10.1021/jp209049k

[72] K. Weinhäupl, C. Lindau, A. Hessel, Y. Wang, C. Schütze, T. Jores, L. Melchionda, B. Schönfisch, H. Kalbacher, B. Bersch, D. Rapaport, M. Brennich, K. Lindorff-Larsen, N. Wiedemann, P. Schanda, Structural basis of membrane protein chaperoning through the mitochondrial intermembrane space. Cell 175, 1365–1379.e25 (2018). 30445040 10.1016/j.cell.2018.10.039 PMC6242696

[73] N. A. Farrow, O. Zhang, J. D. Forman-Kay, L. E. Kay, A heteronuclear correlation experiment for simultaneous determination of ^15^N longitudinal decay and chemical exchange rates of systems in slow equilibrium. J. Biomol. NMR 4, 727–734 (1994). 7919956 10.1007/BF00404280

[74] P. Kiraly, I. Swan, M. Nilsson, G. A. Morris, Improving accuracy in DOSY and diffusion measurements using triaxial field gradients. J. Magn. Reson. 270, 24–30 (2016). 27389639 10.1016/j.jmr.2016.06.011

[75] C. H. Cho, J. Urquidi, S. Singh, G. Wilse Robinson, Thermal offset viscosities of liquid H_2_O, D_2_O and T_2_O. J. Phys. Chem. B 103, 1991–1994 (1999).

[76] C. Arquint, A.-M. Gabryjonczyk, S. Imseng, R. Böhm, E. Sauer, S. Hiller, E. A. Nigg, T. Maier, STIL binding to Polo-box 3 of PLK4 regulates centriole duplication. eLife 4, e07888 (2015). 26188084 10.7554/eLife.07888 PMC4530586

[77] A. S. Altieri, R. A. Byrd, D. P. Hinton, Association of biomolecular systems *via* pulsed field gradient NMR self-diffusion measurements. J. Am. Chem. Soc. 117, 7566–7567 (1995).

[78] L. Shi, L. E. Kay, Tracing an allosteric pathway regulating the activity of the HslV protease. Proc. Natl. Acad. Sci. U.S.A. 111, 2140–2145 (2014). 24469799 10.1073/pnas.1318476111 PMC3926032

[79] B. M. Burmann, J. A. Gerez, I. Matečko-Burmann, S. Campioni, P. Kumari, D. Ghosh, A. Mazur, E. E. Aspholm, D. Šulskis, M. Wawrzyniuk, T. Bock, A. Schmidt, S. G. D. Rüdiger, R. Riek, S. Hiller, Regulation of α-synuclein by chaperones in mammalian cells. Nature 577, 127–132 (2020). 31802003 10.1038/s41586-019-1808-9 PMC6930850

[80] G. Lipari, A. Szabo, Model-Free Approach to the interpretation of nuclear magnetic resonance relaxation in macromolecules. 1. Theory and range of validity. J. Am. Chem. Soc. 104, 4546–4559 (1982).

[81] M. Landau, I. Mayrose, Y. Rosenberg, F. Glaser, E. Martz, T. Pupko, N. Ben-Tal, ConSurf 2005: The projection of evolutionary conservation scores of residues on protein structures. Nucleic Acids Res. 33, W299–W302 (2005). 15980475 10.1093/nar/gki370 PMC1160131

[82] H. Ashkenazy, E. Erez, E. Martz, T. Pupko, N. Ben-Tal, ConSurf 2010: Calculating evolutionary conservation in sequence and structure of proteins and nucleic acids. Nucleic Acids Res. 38, W529–W533 (2010). 20478830 10.1093/nar/gkq399 PMC2896094

[83] K. E. Neet, D. E. Timm, Conformational stability of dimeric proteins: Quantitative studies by equilibrium denaturation. Protein Sci. 3, 2167–2174 (1994). 7756976 10.1002/pro.5560031202 PMC2142765

[84] B. M. Burmann, X. Luo, P. Rösch, M. C. Wahl, M. E. Gottesman, Fine tuning of the *E. coli* NusB:NusE complex affinity to BoxA RNA is required for processive antitermination. Nucleic Acids Res. 38, 314–326 (2009). 19854945 10.1093/nar/gkp736 PMC2800207

[85] M. M. Santoro, D. W. Bolen, Unfolding free energy changes determined by the linear extrapolation method. 1. Unfolding of phenylmethanesulfonyl α-chymotrypsin using different denaturants. Biochemistry 27, 8063–8068 (1988). 3233195 10.1021/bi00421a014

[86] L. Swint, A. D. Robertson, Thermodynamics of unfolding for turkey ovomucoid third domain: Thermal and chemical denaturation. Protein Sci. 2, 2037–2049 (1993). 8298454 10.1002/pro.5560021205 PMC2142319

[87] L. M. Mayr, O. Landt, U. Hahn, F. X. Schmid, Stability and folding kinetics of ribonuclease T1 are strongly altered by the replacement of Cis-proline 39 with alanine. J. Mol. Biol. 231, 897–912 (1993). 8515459 10.1006/jmbi.1993.1336

