# Supplementary material for: Structural basis of DegP protease temperature-dependent activation
Source: Sci Adv. 2021 Dec 8;7(50):eabj1816. doi: 10.1126/sciadv.abj1816 (PMC8654288; doi:10.1126/sciadv.abj1816)
Supplement: Supplementary file 1 — Figs. S1 to S13 Tables S1 to S3 Supplementary Note References [file sciadv.abj1816_sm.pdf]

Science Advances
AAAS

Supplementary Materials for

Structural basis of DegP protease temperature-dependent activation

Darius Šulskis, Johannes Thoma, Björn M. Burmann

*Corresponding author. Email: bjorn.marcus.burmann@gu.se

Published 8 December 2021, *Sci. Adv.* **7**, eabj1816 (2021)
DOI: 10.1126/sciadv.abj1816

This PDF file includes:

Figs. S1 to S13
Tables S1 to S3
Supplementary Note
References

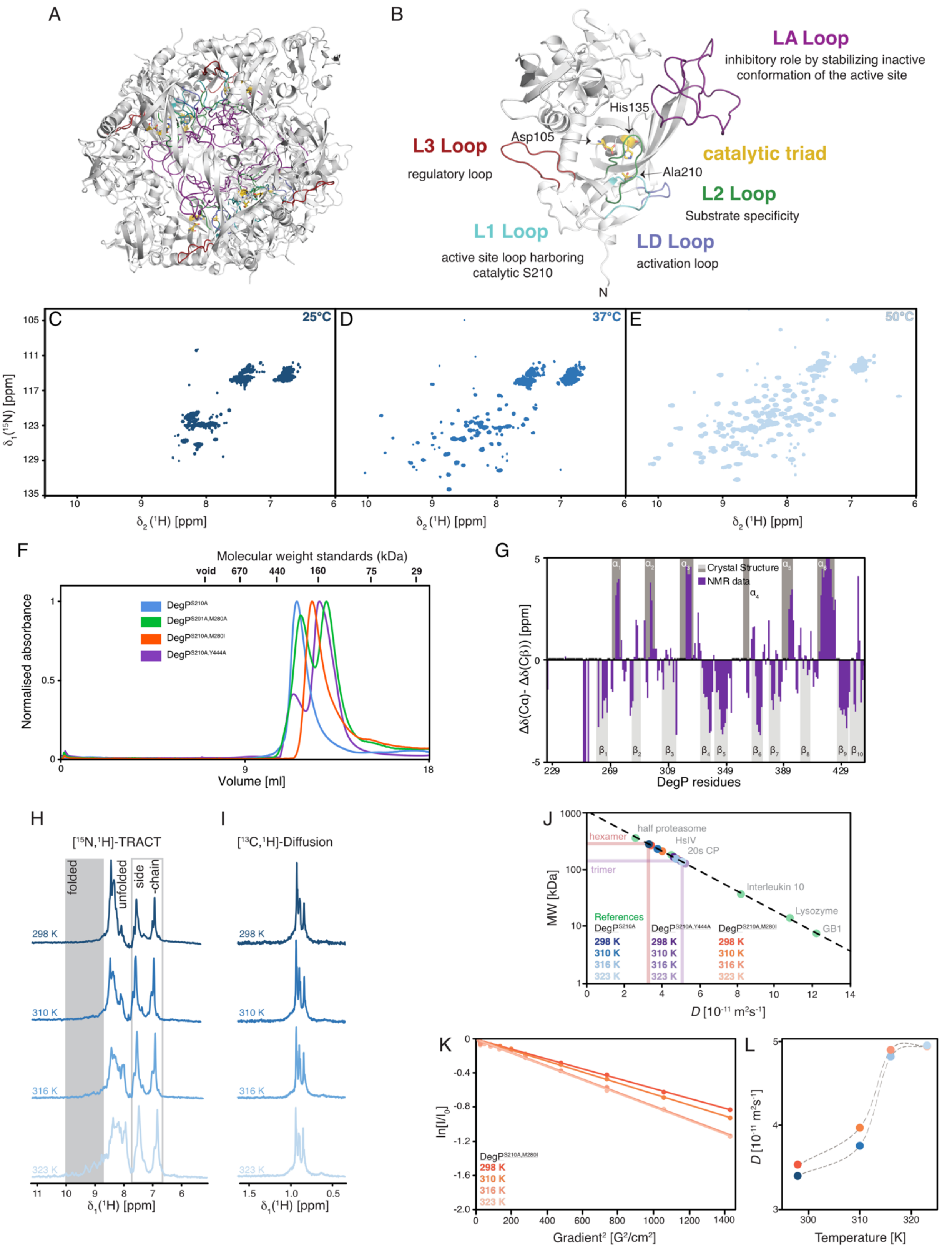

A
B
LA Loop
inhibitory role by stabilizing inactive conformation of the active site
His135
Asp105
L3 Loop
regulatory loop
catalytic triad
Ala210
L2 Loop
Substrate specificity
L1 Loop
active site loop harboring catalytic S210
LD Loop
activation loop
N
C
D
E
25°C
37°C
50°C
δ1(15N) [ppm]
δ2 (1H) [ppm]
F
Molecular weight standards (kDa)
void
670
440
160
75
29
Normalised absorbance
DegP S210A
DegP S201A,M280A
DegP S210A,M280I
DegP S210A,Y444A
Volume [ml]
G
Crystal Structure
NMR data
Δδ(Cα)- Δδ(Cβ)) [ppm]
DegP residues
H
[15N,1H]-TRACT
folded
unfolded
side -chain
298 K
310 K
316 K
323 K
δ1(1H) [ppm]
I
[13C,1H]-Diffusion
J
half proteasome
hexamer
HslV
20s CP
trimer
Interleukin 10
Lysozyme
GB1
References
MW [kDa]
D [10-11 m2s-1]
K
ln[I/I0]
Gradient2 [G2/cm2]
L
Temperature [K]

Fig. S1. Solution studies of full-length DegP.

(**A**) Structural model of the DegP^{S210A} in its inactive hexameric form highlighting the different regulatory loops involved in its proteolytic function. Based on the structural model developed by Figaj *et al.* (*18*). (**B**) Zoom on a DegP^{S210A} monomer with the highlighted regulatory loops together with the catalytic triad and their respective function. (**C–E**) 2D [^{15}N,^{1}H]-NMR spectrum of [*U*-^{2}H,^{15}N]-DegP^{S210A} measured at the indicated temperatures. (**F**) Gel elution profiles of different full-length DegP^{S210A} variants as indicated. Recorded at 8°C in NMR-buffer on a Superdex S200increase 10/300 column (GE Healthcare). The column void volume and the molecular weights of a standard calibration curve are indicated. (**G**) Secondary backbone ^{13}C chemical shifts in solution of the full-length DegP^{S210A,Y444A} protein variant compared to the hexameric X-ray structure (PDB-ID: 1KY9). Positive values indicate α-helix whereas negative values β-strand secondary structure elements. (**H**) δ_1-[^{15}N]-1D cross-sections of 2D [^{15}N, ^{1}H]-TRACT spectra of 500 µM [*U*-^{2}H,^{15}N]-DegP^{S210A} at the indicated temperatures. The grey box indicates the signals for the parts of DegP^{S210A} in stable secondary structures, almost exclusively visible solely at 310 K. Regions for signals of the unfolded segments and amino-acid side chains are indicated. (**I**) Reference 1D-traces of ^{13}C-edited BPP-LED NMR diffusion spectra of 200 µM [*U*-^{2}H, Ile-δ_1-$^{13}CH_3$]–DegP^{S210A} at the indicated temperatures. In contrast to panel **H** this type of experiment provided analysable signals over the complete temperature range chosen. (**J**) Calibration curve for estimating the molecular weight based on the determined molecular diffusion constants. Linear correlation of the molecular weight (MW: logarithmic scale) plotted against the molecular diffusion constants. Published diffusion constants of proteins with known molecular weight are indicated in green (see Methods for details). Temperature-dependent data for DegP^{S210A}, DegP^{S210A,Y444A}, and DegP^{S210A,M280I} are shown as blue, purple or orange gradients, respectively, as indicated. (**K**) Measurement of the molecular diffusion constants with a ^{13}C-methyl-filtered diffusion experiment for DegP^{S210A,M280I}. The logarithm of the signal intensity is plotted against the squared gradient strength of the applied pulsed-field gradients. [*U*-^{2}H, Ile-δ_1-$^{13}CH_3$]–DegP^{S210A,M280I} was measured at different temperatures as indicated. Solid lines are a linear fit to the data. (**L**) Obtained molecular diffusion constants for DegP^{S210A} (blue; data also shown in Fig. 1F) and DegP^{S210A,M280I} (orange) plotted against the temperature. The broken lines serve as a guide to the eyes only.

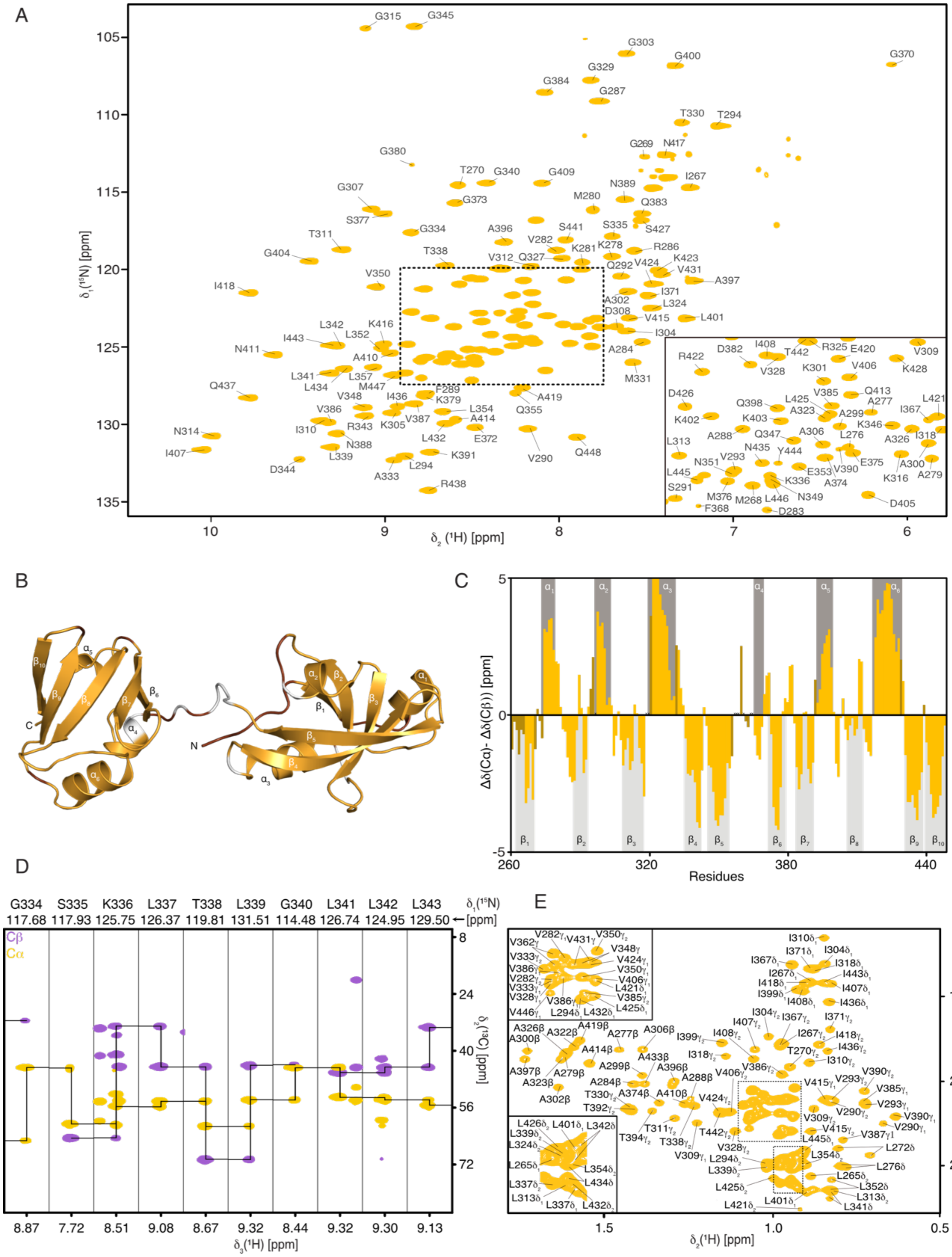
A
δ1(15N) [ppm]
δ2 (1H) [ppm]
B
C
N
C
Δδ(Cα)- Δδ(Cβ)) [ppm]
Residues
D
G334 S335 K336 L337 T338 L339 G340 L341 L342 L343
117.68 117.93 125.75 126.37 119.81 131.51 114.48 126.74 124.95 129.50
δ1(15N) [ppm]
Cβ
Cα
δ2(13C) [ppm]
δ3(1H) [ppm]
8.87 7.72 8.51 9.08 8.67 9.32 8.44 9.32 9.30 9.13
E
δ2(1H) [ppm]
δ1(13C) [ppm]

Fig. S2. The isolated PDZ1–PDZ2 domain construct in solution.

(**A**) 2D [^{15}N,^{1}H]-NMR-spectrum of [*U*-^{15}N,^{13}C]-PDZ1–PDZ2. The sequence-specific resonance assignment obtained from 3D triple-resonance experiments is indicated. (**B**) Cartoon representation of the crystal structure of the PDZ1–PDZ2 domains (PDB-ID: 3OU0) with the assigned resonances highlighted in orange and unassigned residues in white. (**C**) Secondary backbone ^{13}C chemical shift analysis plotted against the PDZ1–PDZ2 residues. Dark shaded bars present residues experiencing line-broadening in [^{15}N,^{1}H]-NMR-spectra that could be assigned unambiguously *via* 2D [^{13}C,^{1}H]-NMR-spectra based on TOCSY-type 3D experiments. The secondary structure elements derived from the crystal structure (PDB-ID: 3OU0) are highlighted in grey. (**D**) Representative strips for residues G74–L83 from a 3D HNCACB spectrum of the PDZ1–PDZ2-domains. (**E**) Methyl-region of a [^{13}C,^{1}H]-NMR-spectrum of [*U*-^{15}N,^{13}C]-PDZ1–PDZ2 with the assignment of the methyl side-chain resonances indicated.

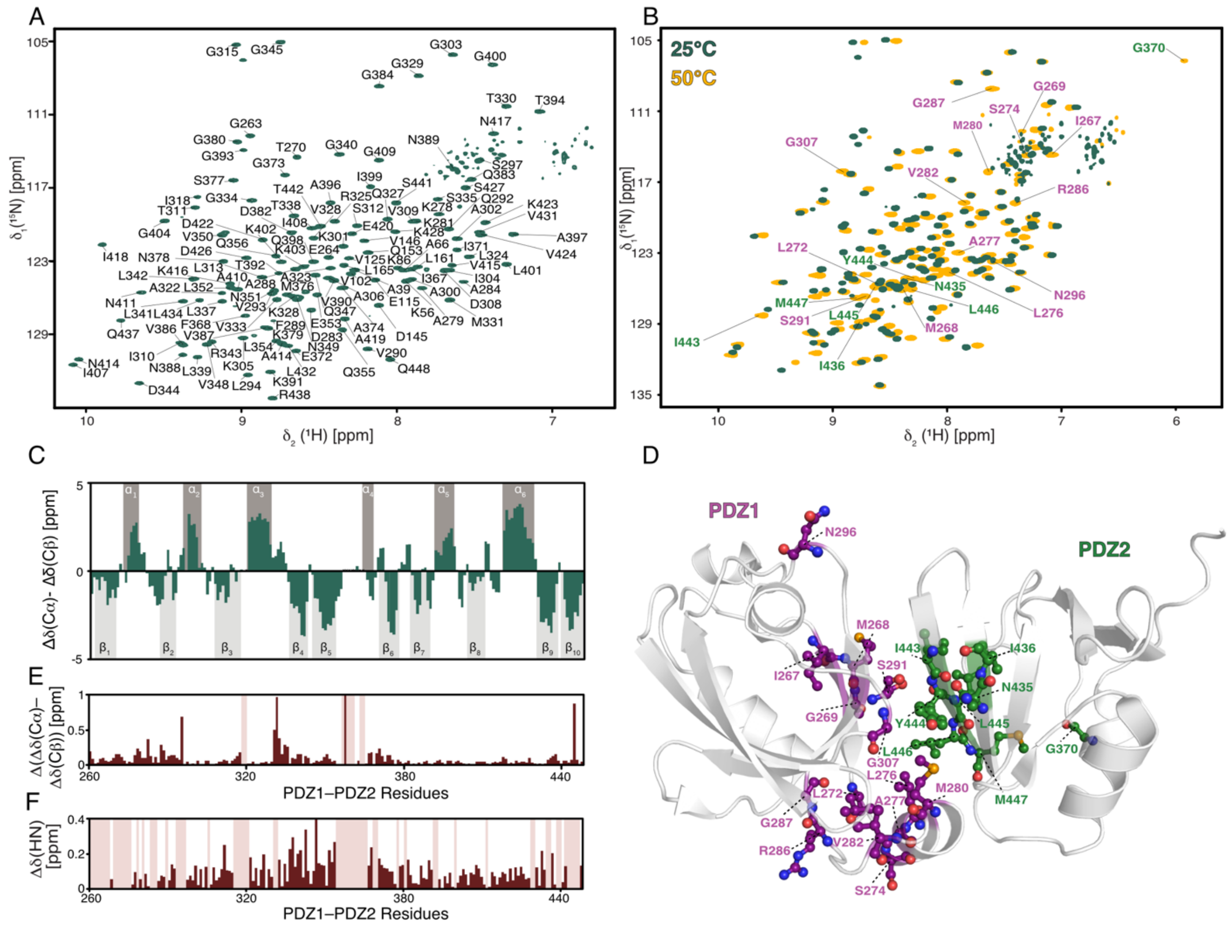

Fig. S3. The isolated PDZ1–PDZ2 domain construct in solution at low temperature.

(**A**) 2D [^{15}N,^{1}H]-NMR-spectrum of [*U*-^{15}N,^{13}C]-PDZ1–PDZ2 at 25°C. The sequence-specific resonance assignment obtained from 3D triple-resonance experiments is indicated. **(B)** 2D [^{15}N,^{1}H]-NMR spectra of [*U*-^{15}N, ^{13}C]-PDZ1–PDZ2 measured at 25°C (green) and at 50°C (orange). Characteristic peaks disappearing at the lower temperature are indicated. (**C**) Secondary backbone ^{13}C chemical shift analysis plotted against the PDZ1–PDZ2 residues. The secondary structure elements derived from the crystal structure (PDB-ID: 3OU0) are highlighted in grey. **(D)** Visualization of the observed line-broadened residues upon domain interaction at low temperature are indicated in purple (PDZ1) and green (PDZ2). (**E**) The difference of the secondary backbone ^{13}C chemical shifts at the different temperatures ($\Delta(\Delta\delta(^{13}C\alpha)$-$\Delta\delta(^{13}C\beta))$ are shown in dark red. (**F**) Combined chemical shift differences of the amide moieties plotted versus the PDZ1–PDZ2 amino acid residue number. In panels, **E** and **F** broadened residues are indicated by the red shading.

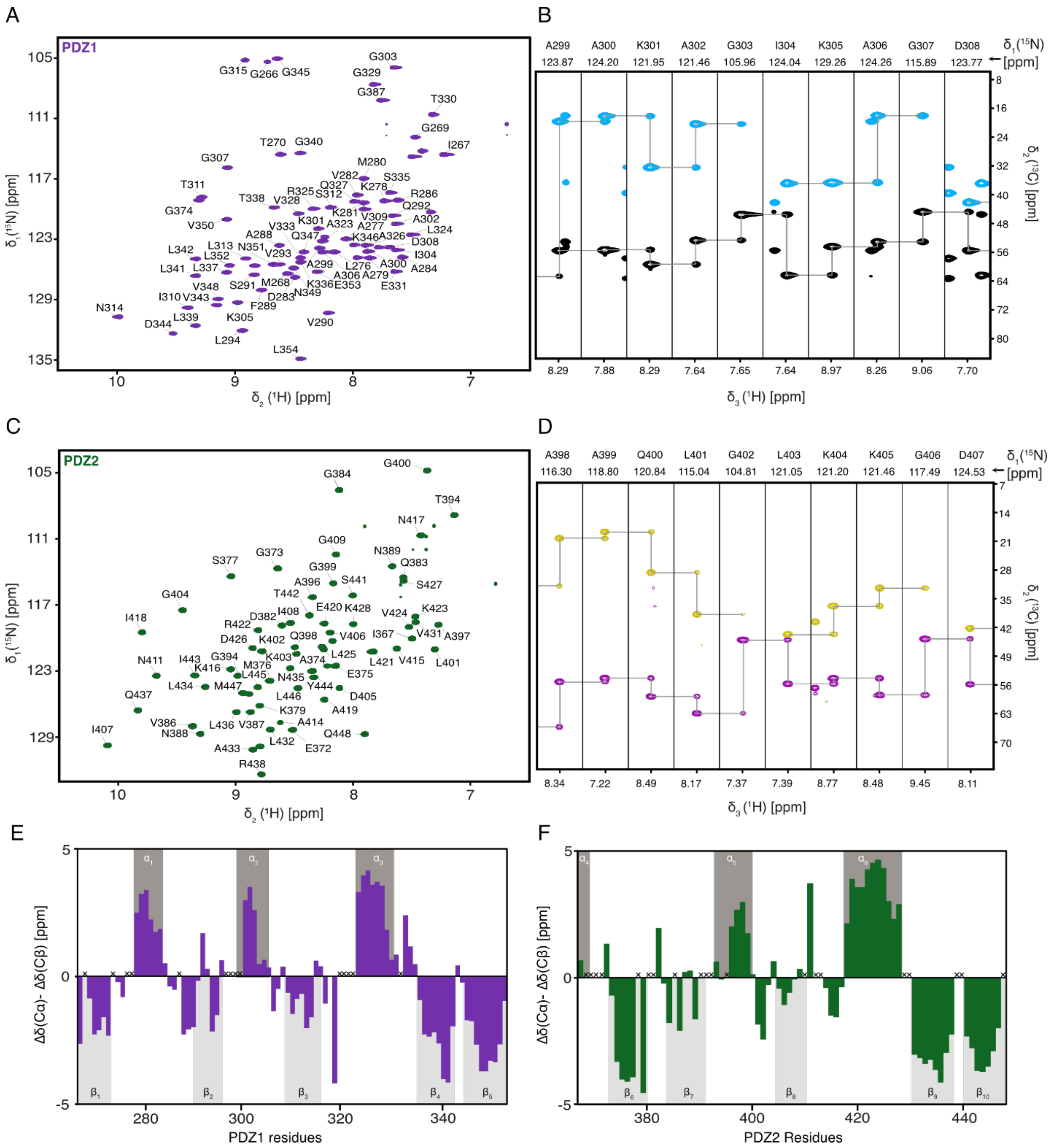

Fig. S4. The isolated PDZ1 and PDZ2 domains in solution.

(**A**) 2D [^{15}N,^{1}H]-NMR-spectrum of [U-^{15}N,^{13}C]-PDZ1. The sequence-specific resonance assignment obtained from 3D triple-resonance experiments is indicated. (**B**) Representative strips for residues A299–D308 from a 3D HNCACB spectrum of the PDZ1-domain. (**C**) 2D [^{15}N,^{1}H]-NMR-spectrum of [U-^{15}N,^{13}C]-PDZ1. The sequence-specific resonance assignment obtained from 3D triple-resonance experiments is indicated. (**D**) Representative strips for residues A398–D407

from a 3D HNCACB spectrum of the PDZ2-domain. (**E, F**) Secondary backbone ^{13}C chemical shift analysis plotted against the PDZ1 (**E**) and PDZ2 (**F**) residues. The secondary structure elements derived from the crystal structure are highlighted in grey and missing assignments are indicated by an “x”.

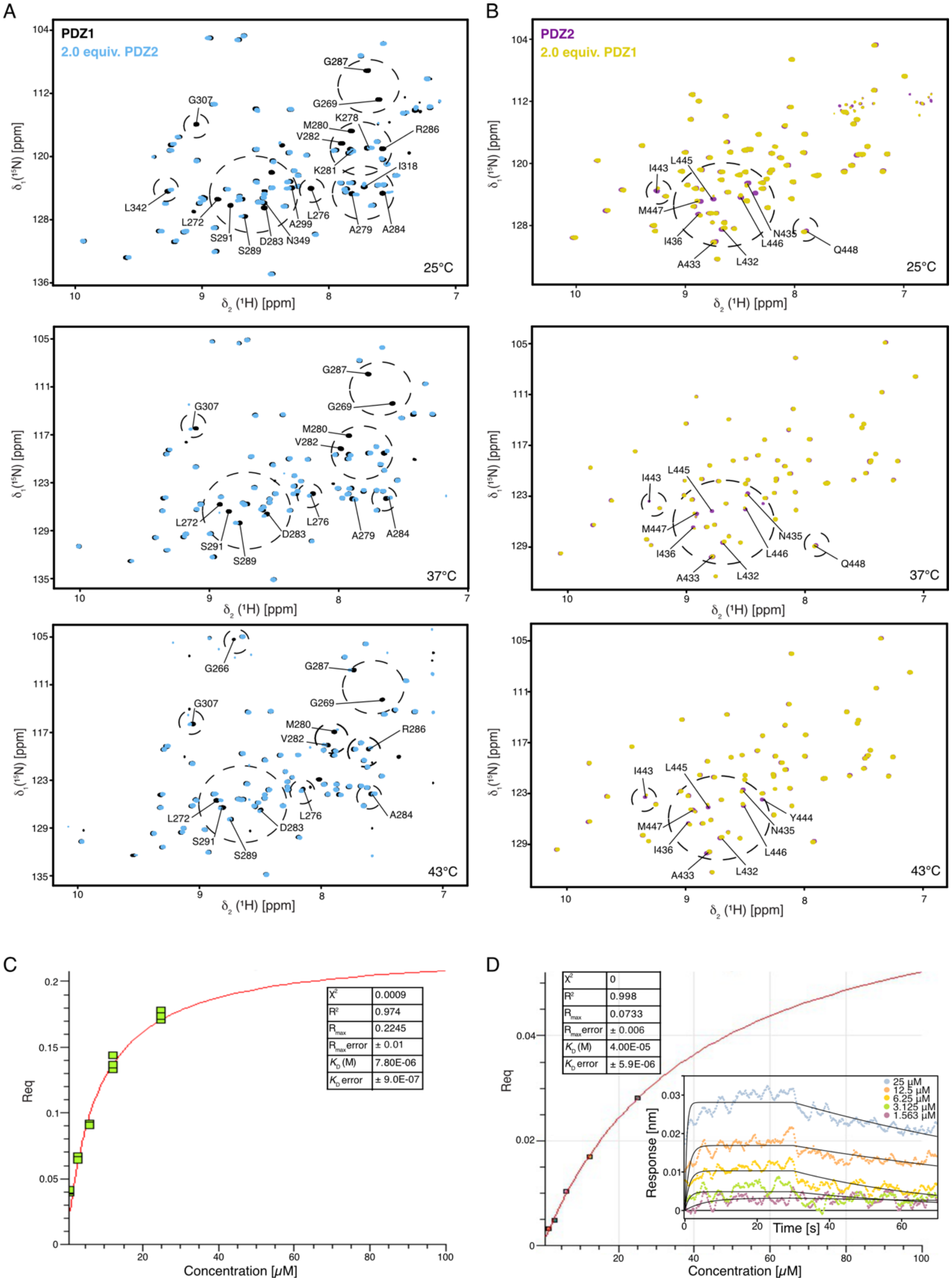

Fig. S5. Temperature dependence of the PDZ1:PDZ2 interaction.

(**A, B**) 2D [^{15}N,^{1}H]-NMR spectra of [*U*-^{15}N]-PDZ1 (**A**, purple) and [*U*-^{15}N]-PDZ2 (**B**, black) as well as after the addition of two molar equivalents of the respective other domain. Titrations were

measured at 25°C, 37°C, and 43°C, as indicated. (**C**) Steady-state analysis of the BLI-curves is shown in Fig. 2F. Curves were measured and analyzed in triplicates (green data points). The red curve is the least-squares fit to the data. (**D**) Steady-state analysis of BLI-curves shown in the inset. The red curve is the least-squares fit to the data. Inset shows the kinetic analysis by biolayer interferometry (BLI) of the PDZ1–PDZ2 interaction. PDZ2 binding to the biotinylated-PDZ1 domain was probed at 25°C. Analyte concentrations are indicated in the figure. Non-linear least-squares fits to the experimental data are indicated by black lines. The resulting K_Ds and indicators for the fitting quality (R^2, χ^2) are indicated for panels **C** and **D**.

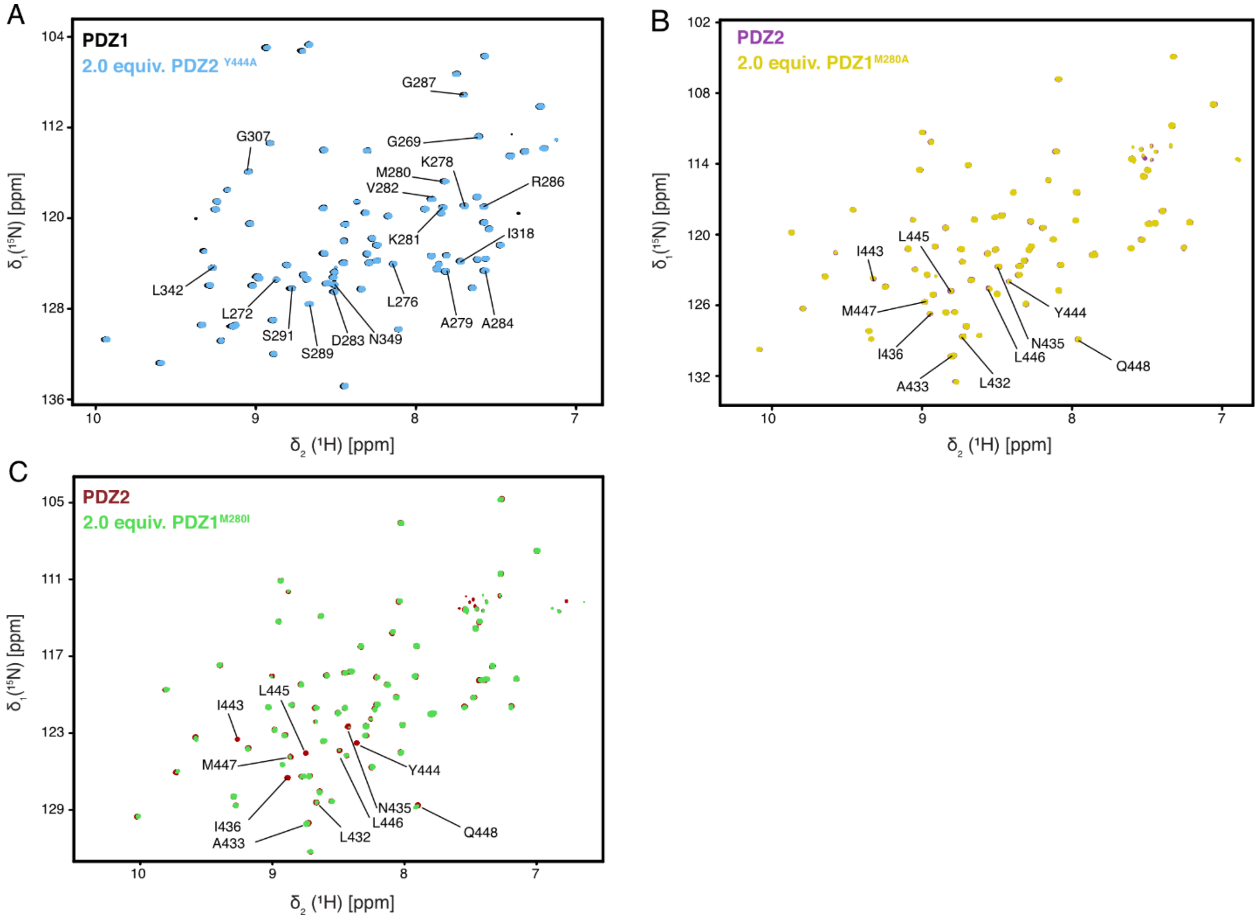

Fig. S6. Interactions of the PDZ1^{M280A}, PDZ1^{M280I}, and PDZ2^{Y444A} mutants.

(**A, B, C**), 2D [^{15}N,^{1}H]-NMR spectra of [*U*-^{15}N]-PDZ1 (**A**, black), [*U*-^{15}N]-PDZ2 (**B**, **C**, purple) as well as after addition of two molar equivalents of the respective other domain carrying the indicated single-point mutation. Titrations were measured at 25°C.

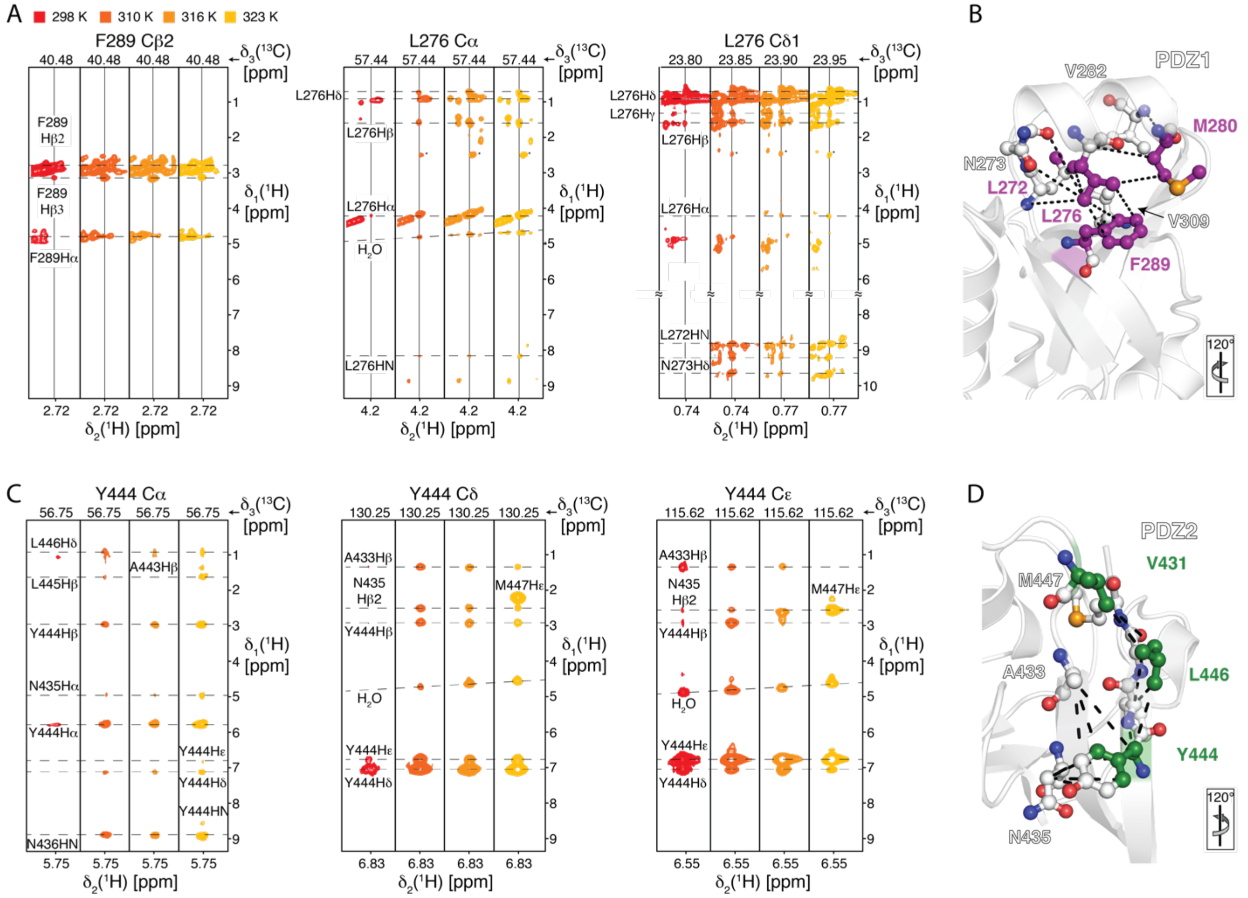

Fig. S7. Temperature-dependent analysis of the NOE-network stabilizing the PDZ1–PDZ2 interface

(**A, B**) Representative NOE strips from NOESY experiments at the indicated temperatures. Intramolecular NOE cross-peaks showing the NOE-network on PDZ1 (**A**) and PDZ2 (**B**) stabilizing the respective interaction regions are indicated. (**C, D**) Visualization of the detected intramolecular NOEs (dashed lines) on the structure of the individual domains (PDZ1 (**C**) and PDZ2 (**D**)). Broadened residues upon domain interaction at low temperature are indicated in purple (PDZ1) and green (PDZ2) as shown in Fig. 3H.

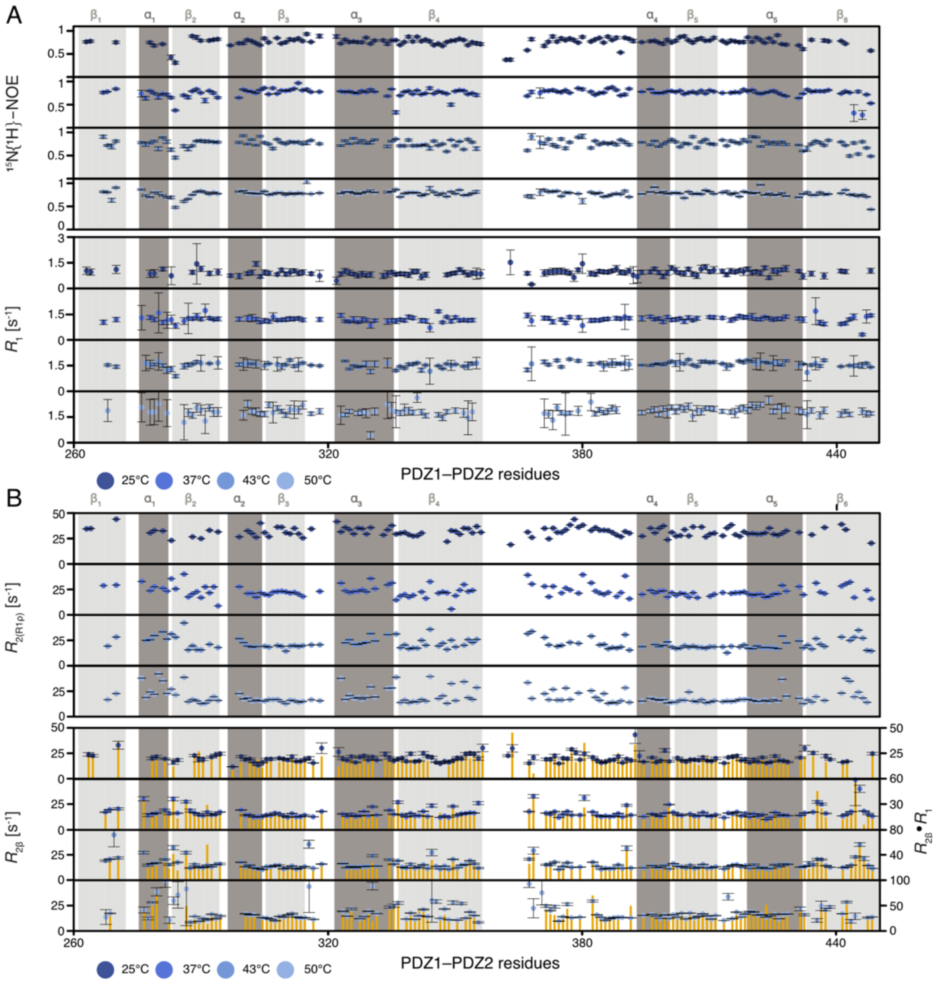

Fig. S8. Temperature dependence of the PDZ1-PDZ2 backbone dynamics.

(**A**) Local backbone dynamics on the pico– to nanosecond timescale probed by hetNOE and R_1 measurements. (**B**) Obtained transversal relaxation rates ($R_{2\beta}$ and $R_{2(R1\rho)}$) reporting on micro- to millisecond motions. The $R_{2\beta}$ rates are plotted along with the $R_1 \bullet R_{2\beta}$ values (yellow bars). All data are plotted against the PDZ1–PDZ2 residues at the indicated temperatures ranging from 25–50°C.

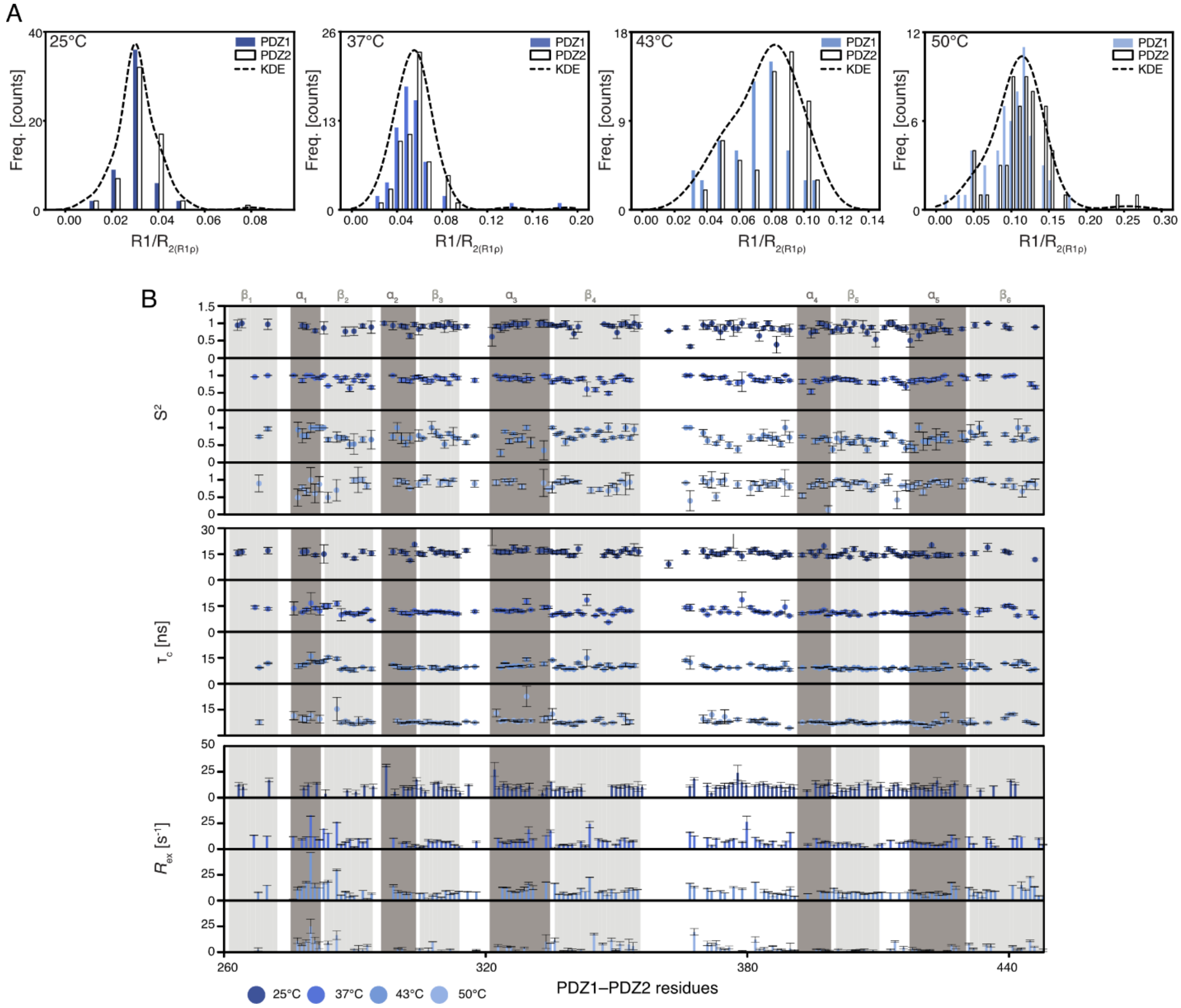

Fig. S9. Analysis of the temperature-dependent PDZ1-PDZ2 backbone dynamics.

(**A**) Histogram of $R_1/R_{2(\mathrm{R1\rho})}$ values of the PDZ1 (solid bars) and PDZ2 (open bars) over the indicated temperature range, dashed lines correspond to kernel density estimation (KDE) of both domain values using a bandwidth of *h=0.5*. A unimodal distribution indicates coupled motions, whereas a more bimodal distribution indicates partially decoupled motion of the individual domains in the PDZ1–PDZ2 construct. (**B**) Temperature dependence of the sub-nanosecond order parameter S^2 and the exchange rate R_{ex}, reporting on possible conformational exchange contributions on the micro- to millisecond timescale, calculated using the Lipari-Szabo model-free approach. Rotational correlation time (τ_c) is determined from the ratio of R_1 and $R_{2(\mathrm{R1\rho})}$. All data are plotted against the PDZ1–PDZ2 residues at the indicated temperatures ranging from 25–50°C.

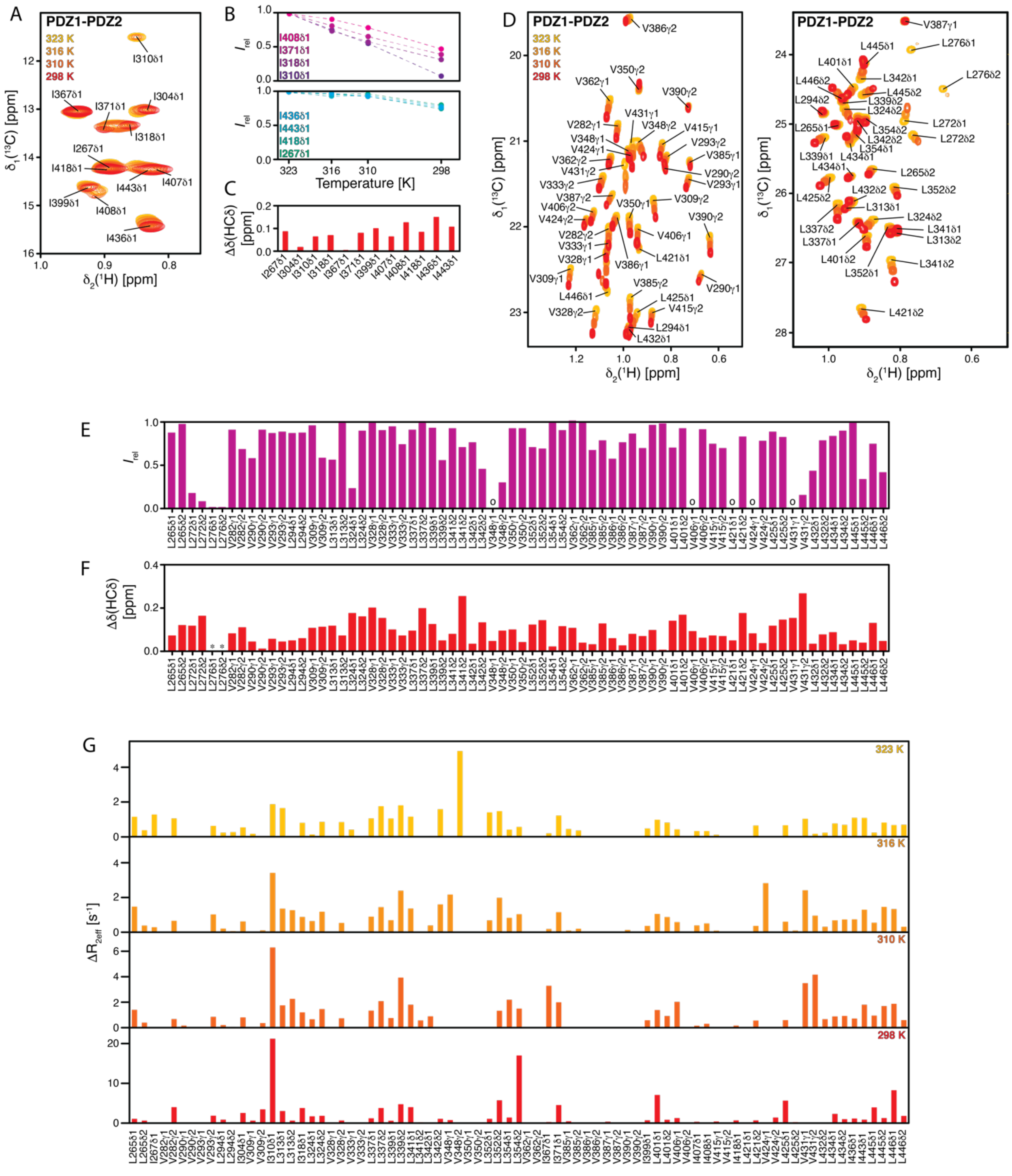

Fig. S10. Methyl group dynamics.

(**A**) Isoleucine methyl region of a 2D [^{13}C,^{1}H]-NMR spectrum of [*U*-^{13}C,^{15}N]-PDZ1–PDZ2 at different temperatures as indicated. (**B, C**) The temperature dependence of the isoleucine signal intensities (**B**) and chemical shift changes (**C**) for the PDZ1–PDZ2 construct. The broken lines in panel **B** serve as a guide to the eyes only. (**D**) Leucine and valine methyl region of a 2D [^{13}C,^{1}H]-

NMR spectrum of [U-^{2}H, Leu-$\delta_{1,2}$-Val-$\gamma_{1,2}$ $^{13}CH_3$]-PDZ1–PDZ2 at different temperatures as indicated. (**E, F**) The temperature dependence of the leucine/valine signal intensities (**E**) and chemical shift changes (**F**) for the PDZ1–PDZ2 construct. o in panel **E** indicates overlapping peaks, whereas * in panel **F** indicates severe line-broadening beyond detection. (**G**) ΔR_{2eff} values for isoleucine, leucine, and valine methyl groups, obtained from the difference of R_{2eff} at the lowest and highest CPMG frequency υ_{CPMG}, at the indicated temperatures.

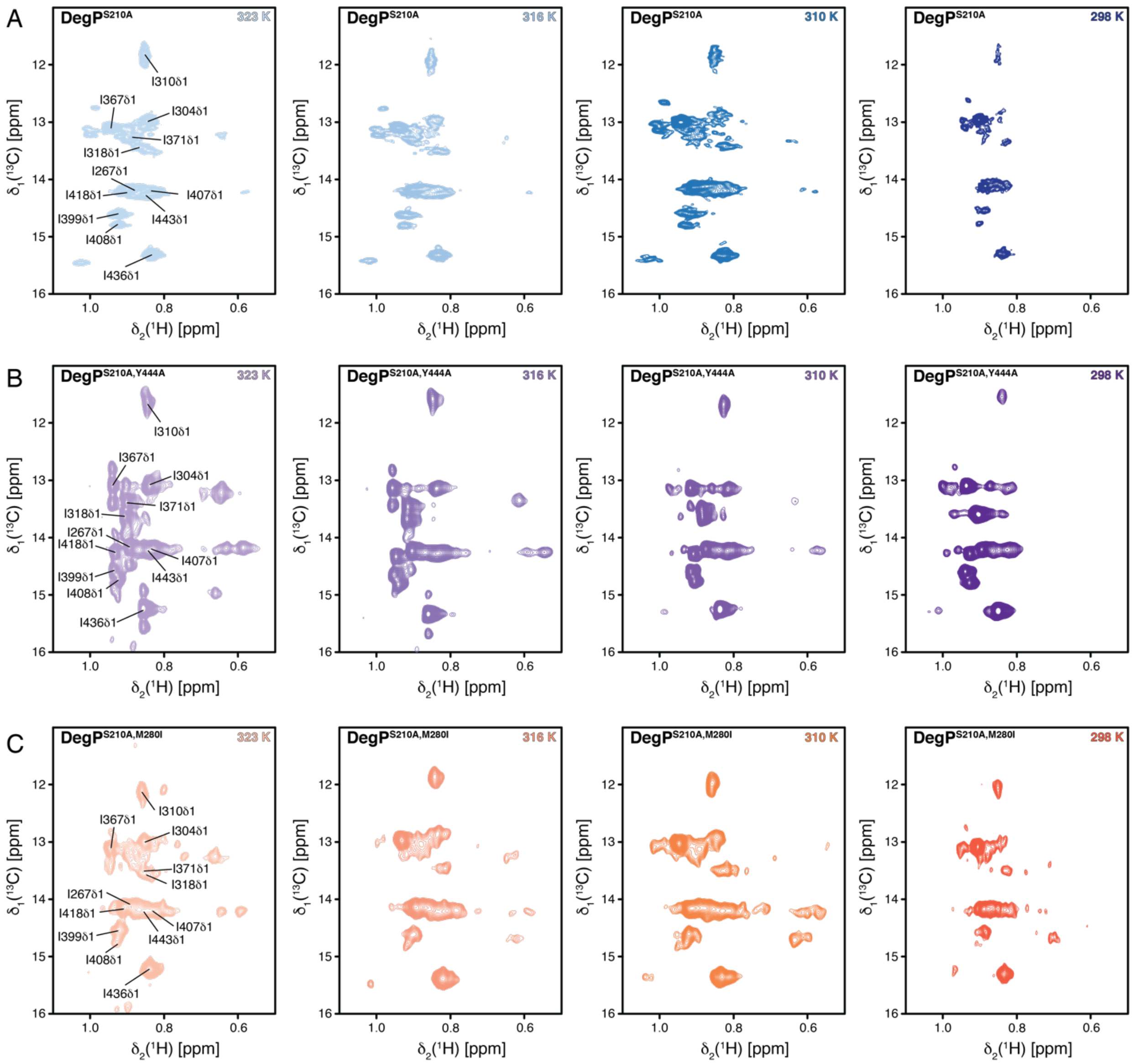

Fig. S11. Isoleucine spectra of full-length DegP^{S210A}-variants

(**A–C**) 2D [^{13}C,^{1}H]-NMR spectra of [U-^{2}H, Ile-δ_1-$^{13}CH_3$]–DegP^{S210A} (**A**), [U-^{2}H, Ile-δ_1-$^{13}CH_3$]–DegP^{S210A,Y444A} (**B**), and [U-^{2}H, Ile-δ_1-$^{13}CH_3$]–DegP^{S210A,M280I} (**C**) measured at the indicated temperatures. The sequence-specific resonance assignments of selected isoleucine resonances derived from the isolated PDZ1–PDZ2 domain are indicated.

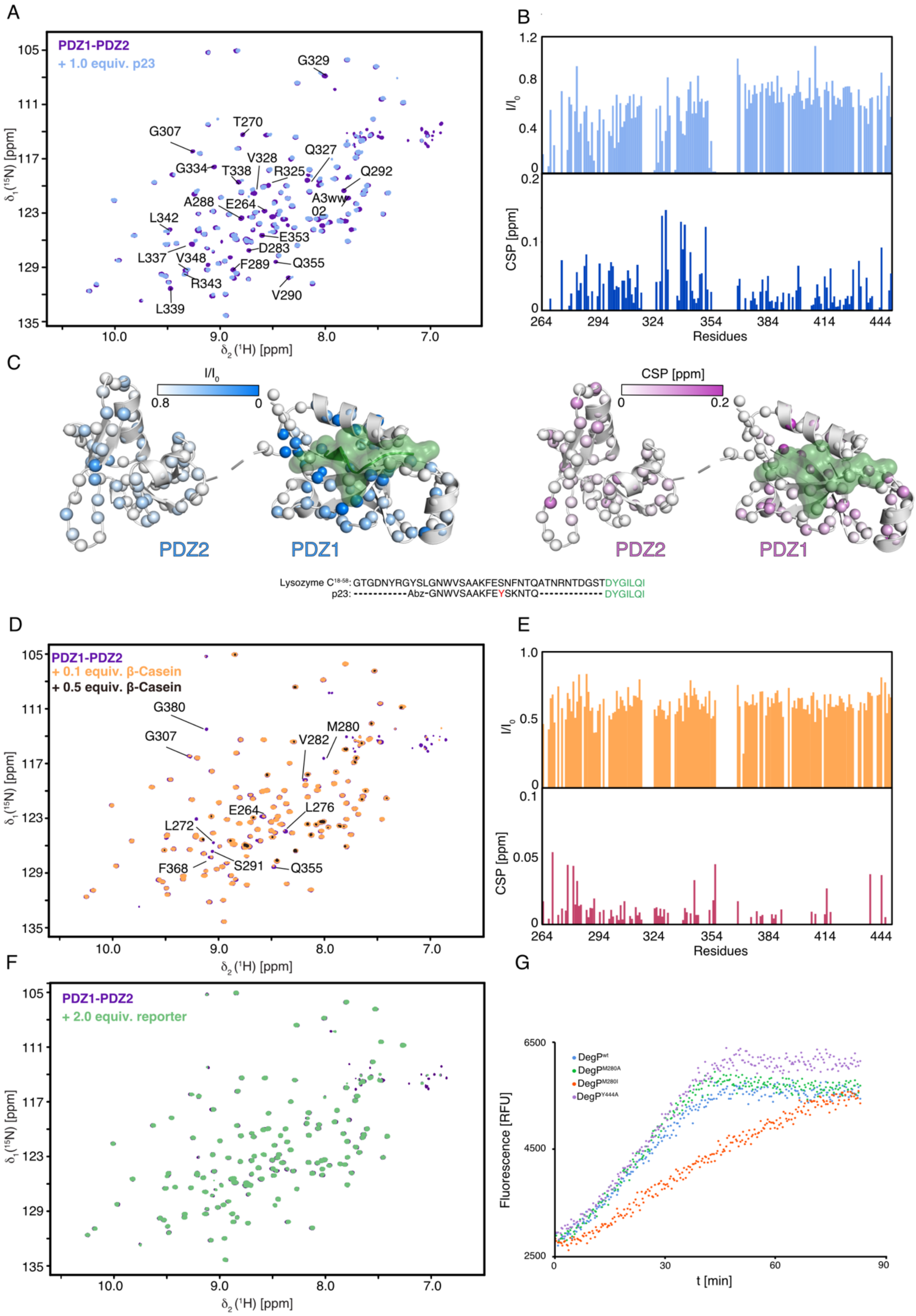

A
PDZ1-PDZ2
+ 1.0 equiv. p23
G329
T270
G307
G334
T338
V328
Q327
R325
Q292
A288
E264
A3ww
02
L342
E353
D283
L337
V348
F289
Q355
R343
V290
L339
δ1 (15N) [ppm]
δ2 (1H) [ppm]
B
I/I0
CSP [ppm]
Residues
C
I/I0
CSP [ppm]
PDZ2
PDZ1
Lysozyme C18-58: GTGDNYRGYSLGNWVSAAKFESNFNTQATNRNTDGSTDYGILQI
p23: ----------Abz-GNWVSAAKFEYSKNTQ-------------DYGILQI
D
PDZ1-PDZ2
+ 0.1 equiv. β-Casein
+ 0.5 equiv. β-Casein
G380
M280
G307
V282
E264
L276
L272
F368
S291
Q355
E
I/I0
CSP [ppm]
Residues
F
PDZ1-PDZ2
+ 2.0 equiv. reporter
G
DegPwt
DegPM280A
DegPM280I
DegPY444A
Fluorescence [RFU]
t [min]

Fig. S12. PDZ1-PDZ2 construct interactions with different substrates.

(**A**) 2D [^{15}N,^{1}H]-NMR spectra of [*U*-^{15}N]-PDZ1–PDZ2 (purple) and after the addition of 1 molar equivalent of p23 (blue). All NMR titrations were measured at 37°C. (**B**) Residue-resolved backbone amide NMR signal attenuation (I/I_0) and detected signal chemical shift perturbations (CSP) of the p23 titration. (**C**) Observed p23 titration effects plotted on the previously reported crystal structure of the DegP:Lysozyme $C^{15\text{-}58}$ (green) complex (3OTP) (*14*). The amide moieties of the PDZ1–PDZ2 construct are shown as spheres. Signal attenuation values are indicated by the white to blue and CSP values by the white to the purple gradient. Inset: Sequence alignment of Lysozyme $C^{15–58}$ and p23 for the visible residues highlighted in green. (**D**) 2D [^{15}N,^{1}H]-NMR spectra of [*U*-^{15}N]-PDZ1–PDZ2 (purple) and after the addition 0.1 (orange) and 0.5 (black) molar equivalents of β-Casein. (**E**) Residue-resolved backbone amide NMR signal attenuation (I/I_0) and detected signal chemical shift perturbations (CSP) of the β-Casein titration (0.1 equivalents). (**F**) 2D [^{15}N,^{1}H]-NMR spectra of [*U*-^{15}N]-PDZ1–PDZ2 (purple) and after the addition 2 molar equivalents of the non-activating reporter peptide. (**G**) Proteolysis of p23 (7.5 μM) by DegP and its variants (0.2 μM of monomer concentration) at 25°C.

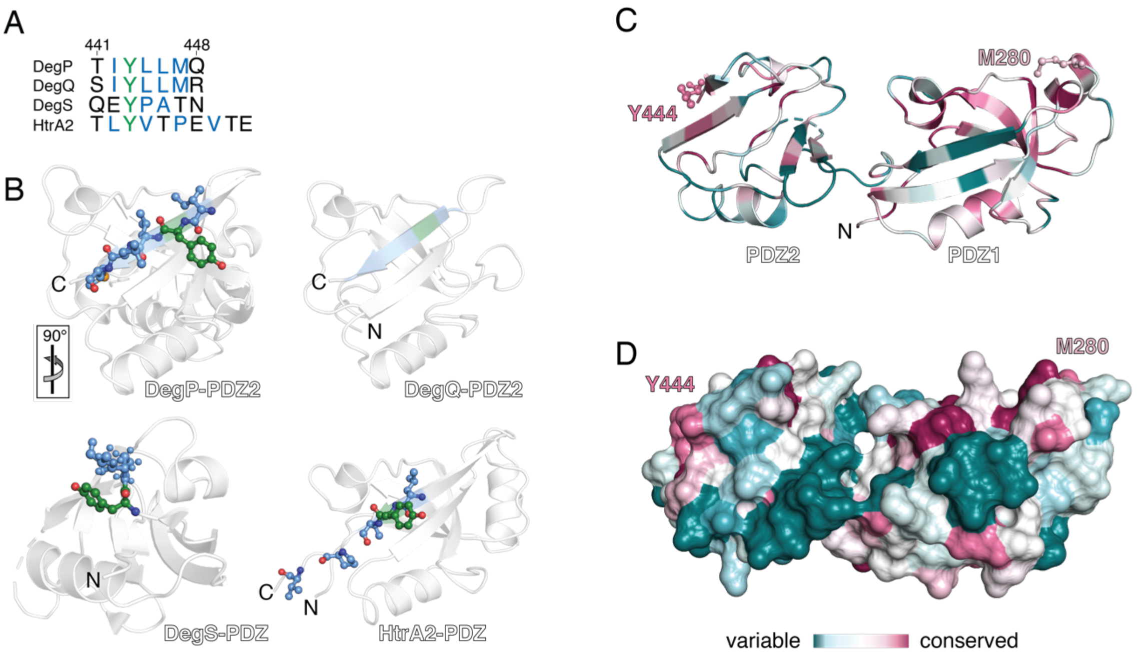

Fig. S13. Sequence conservation analysis of the interdomain lock.

(**A**) Sequence comparison of the β10-strand containing Y444 in the PDZ of DegP with corresponding domains of related bacterial periplasmic DegQ and DegS as well as human mitochondrial HtrA2. (**B**) Cartoon representation of the DegP-PDZ2 (PDB-ID: 3OU0) with focus on Y444 and adjacent hydrophobic residues forming a hydrophobic patch facilitating the inter-domain lock (*23*). For the DegQ-PDZ2 only a medium resolution structure at 7.5Å (PDB-ID: 4A8C) is available without any side-chain information (*22*). In contrast to DegP and DegQ, DegS has only a single PDZ-domain (PDB-ID: 4RQY). Similarly, human mitochondrial HtrA2 bears also a single PDZ-domain (PDB-ID: 1LCY), for which recently the involvement of the high-lighted tyrosine in an oligomeric resting state stabilization could be shown (*21*). (**C, D**) Cartoon **(C)** and surface **(D)** representation of the DegP PDZ1-PDZ2 domains displaying the degree of conservation with the indicated gradient ranging from 0 (variable) to 9 (conserved). Positions of M280, 7/9 conservation score, and Y444, 8/9 conservation score are indicated. Analysis was performed *via* the ConSurf (Tel Aviv University, Israel) server (*81, 82*).

Table S1. Summary of the obtained diffusion coefficients.

Temperature	$DegP^{S210A}$	$DegP^{S210A,Y444A}$	$DegP^{S210A,M280I}$
**25°C**	$3.41 \pm 0.14 \cdot 10^{-7}$ cm^2s^{-1}	$4.64 \pm 0.09 \cdot 10^{-7}$ cm^2s^{-1}	$3.53 \pm 0.14 \cdot 10^{-7}$ cm^2s^{-1}
**37°C**	$3.81 \pm 0.08 \cdot 10^{-7}$ cm^2s^{-1}	$4.81 \pm 0.10 \cdot 10^{-7}$ cm^2s^{-1}	$3.97 \pm 0.08 \cdot 10^{-7}$ cm^2s^{-1}
**43°C**	$4.83 \pm 0.19 \cdot 10^{-7}$ cm^2s^{-1}	$4.96 \pm 0.10 \cdot 10^{-7}$ cm^2s^{-1}	$4.89 \pm 0.20 \cdot 10^{-7}$ cm^2s^{-1}
**50°C**	$4.94 \pm 0.10 \cdot 10^{-7}$ cm^2s^{-1}	$4.99 \pm 0.10 \cdot 10^{-7}$ cm^2s^{-1}	$4.94 \pm 0.10 \cdot 10^{-7}$ cm^2s^{-1}

Table S2. Summary of the calculated diffusion tensor values.

Temperature	D_x	D_y	D_z	$D_{\|}/D_{\perp}$ [a]	α°	β°	γ°
**25°C**	1.196 ± 0.039	1.818 ± 0.041	1.956 ± 0.414	1.30	23.10 ± 34.25	19.96 ± 22.02	54.48 ± 33.97
**37°C**	1.859 ± 0.038	2.238 ± 0.041	2.572 ± 0.043	1.26	-27.07 ±13.61	22.47 ± 5.93	55.79 ± 13.94
**43°C**	2.249 ± 0.043	2.768 ± 0.039	3.165 ± 0.049	1.26	-15.87 ± 5.21	41.77 ± 5.21	72.91 ± 4.09
**50°C**	2.431 ± 0.104	3.989 ± 0.117	4.325 ± 0.112	1.35	18.37 ± 15.01	-37.93 ± 25.15	34.38 ± 15.35

Principle values of the diffusion tensor (10^{-8} s^{-1}) as determined for axially symmetric diffusion tensors with Tensor2 (*65*). Relaxation data determined at a proton Larmor frequency of 700 MHz (16.4 T) was used with standard values for the length of the N–H bond, $r_{N\text{-}H}$ of 1.02 Å, and the chemical shift anisotropy (CSA) of -172 ppm. In addition, the refined high resolution DegP structure was used (PDB: 3OU0). $D_{\|}/D_{\perp}$ was calculated as follows: $D_{\|} = (D\text{x} + \text{Dy})/2$ and $D_{\perp} = D_z$

Table S3. Plasmids and respective primers used in this study.

Plasmid	Primer	Sequence
**pDS5 (6xHis-DegP^{S210A,Y444A})**<br>**pDS37 (6xHis-SUMO-PDZ2^{Y444A})**<br>**pDS45 (6xHis-DegP^{Y444A})**	DegP_Y444A_frw<br>DegP_Y444A_rev	5'-GATTACTGCATTAACAGGGCGATGGTGCTGTCGCCGCG-3'<br>5'-CGCGGCGACAGCACCATCGCCCTGTTAATGCAGTAATC-3'
**pDS41 (6xHis-DegP^{S210A,M280A})**<br>**pDS44 (6xHis-DegP^{M280A})**<br>**pDS40 (6xHis-SUMO-PDZ1^{M280A})**<br>**pDS39(6xHis-SUMO-PDZ1–PDZ2^{M280A})**	DegP_M280A_frw<br>DegP_M280A_rev	5'-CTTTCGCCGCTTTCGCCAGTTCGGAGTTCAG-3'<br>5'-CTTTCGCCGCTTTCGCCAGTTCGGAGTTCAG-3'
**pDS52 (6xHis-DegP^{M280I})**<br>**pDS46 (6xHis-DegP^{S210A,M280I})**	DegP_M280I_frw<br>DegP_M280I_rev	5'-CTTTAATCGCTTTCGCCAGTTCGGAGTTCAG-3'<br>5'-GCGATTAAAGTTGACGCCCAGCGCGGTGC-3'
**pDS35(6xHis-DegP)**	DegP_A210S_frw<br>DegP_A210S_rev	5’-CGTGGTAACTCCGGTGGTGCGCTGG-3’<br>5’-CCACCGGAGTTACCACGGTTGATCGC-3’
**pDS10(6xHis-SUMO-PDZ1)**	pET15_5206_fwd<br>SUMO_PDZ1PDZ2_fwd<br>SUMO_PDZ1PDZ2_rev<br>PDZ1_XhoI_rev	5'-ATCGAGATCTCGATCCCGCG-3'<br>5'-GATTGGCGGTAAACGCGGTGAGCTGGG-3'<br>5'-CACCGCGTTTACCGCCAATCTGTTCCAG-3'<br>5'-GTGCTCGAGTTACAGTTCCAGGTTCACG-3'
**pDS11(6xHis-SUMO-PDZ2)**	pET15_5206_fwd<br>SUMO_PDZ2_fwd<br>SUMO_PDZ2_rev<br>PDZ1PDZ2_XhoI_rev	5'-ATCGAGATCTCGATCCCGCG-3'<br>5'-GATTGGCGGTCAGAATCAGGTTGATTCCAG-3'<br>5'-CTGATTCTGACCGCCAATCTGTTCCAG-3'<br>5'-GTGCTCGAGTTACTGCATTAACAGGTAGATG-3'
**pDS12(6xHis-SUMO-PDZ1–PDZ2)**	pET15_5206_fwd<br>PDZ1PDZ2_XhoI_rev<br>SUMO_PDZ1PDZ2_fwd<br>PDZ1PDZ2_XhoI_rev	5'-ATCGAGATCTCGATCCCGCG-3'<br>5'-GTGCTCGAGTTACTGCATTAACAGGTAGATG-3'<br>5'-GATTGGCGGTAAACGCGGTGAGCTGGG-3'<br>5'-GTGCTCGAGTTACTGCATTAACAGGTAGATG-3'

Supplementary Note:

Thermodynamic characterization of the trimer-hexamer–equilibrium by a two-state model

We have used 2D [^{13}C,^{1}H]-NMR experiments over the temperature range from 298 K to 323 K to measure the trimer-hexamer equilibrium in a temperature-dependent manner based on a [U-^{2}H, Ile-δ_1-$^{13}CH_3$]–DegP^{S210A} sample. The different temperature-dependent equilibria contributing to the complete thermodynamic equilibrium of DegP are:

$$\boldsymbol{P_6 \rightleftarrows 2P_3 \rightleftarrows 6D_1} \quad (1)$$

where *P* represents the folded DegP-monomer and *D* the unfolded DegP-monomer. As the employed temperature range did not lead to the denaturation of the DegP-monomer the right part of this thermal equilibrium leading to the unfolded state can be neglected (*83*) enabling the focus on the underlying hexamer-to-trimer equilibrium. Additionally, as the larger oligomeric assemblies of DegP can only form in the presence of substrate proteins (*12–14*), these $DegP_{12}$ and $DegP_{24}$ forms can be excluded from the analysis of the temperature-dependent equilibrium of substrate-free DegP. Therefore, considering the trimer-hexamer equilibrium the total protein concentration $\boldsymbol{C_{total}}$ (in monomers) can be expressed as follows:

$$\boldsymbol{C_{total} = 6[P_6] + 3[P_3]} \quad (2)$$

From this the fractional populations of monomers residing within the trimeric (F_{trimer}) and hexameric (F_{hexamer}) state are given:

$$\boldsymbol{F_{trimer} = \frac{3[P_3]}{C_{total}}} \quad (3)$$

$$\boldsymbol{F_{hexamer} = \frac{6[P_6]}{C_{total}}} \quad (4)$$

The fitting of the thermodynamic transition was based on the two well-separated isoleucine δ_1-resonances (I310 and I408). Furthermore, the eventually different relaxation properties of the hexamer and the trimer were neglected as solely the intensity of the peaks in a temperature-dependent manner could be analyzed, thus providing a limited initial estimate into the

thermodynamics of hexamer dissociation. Furthermore, an all-or-nothing model was assumed, which only accounts for trimers and hexamers enabling us to base our evaluations on a two-state model.

This assumption is in agreement with the proposed model of a trimeric building block, which is also in line with the experimental data presented here as well as in previous studies (*15, 16*).

Taking all these considerations into account and making the approximation of the formation of 100% hexamer at 25°C and 100% at 50°C, the observed signal intensity ($\boldsymbol{I_{obs}}$) at each temperature can be expressed as follows:

$$\boldsymbol{I_{obs} = F_{trimer} - F_{hexamer}} \tag{5}$$

By fitting the baselines and transition region of the curve simultaneously the ensuing transition curve can be described (*84*) by:

$$\boldsymbol{I_{obs}} = \frac{(y_{trimer} + m_{trimer} * T_m)}{\left(1 + e^{(\Delta H/R * (1/T_m - 1/T))}\right)} + \frac{(y_{hexamer} + m_{hexamer} * T) * e^{(\Delta H/R * (1/T_m - 1/T))}}{(1 + e^{(\Delta H/R * (1/T_m - 1/T))})} \tag{6}$$

where $\boldsymbol{y_{hexamer}}$ and $\boldsymbol{y_{trimer}}$ are the y-intercepts of the baseline corresponding to the hexameric and trimeric species, respectively; $\boldsymbol{m_{hexamer}}$ and $\boldsymbol{m_{trimer}}$ are the slopes of the baseline. $\boldsymbol{\Delta H}$ is the enthalpy at the transition point temperature ($\boldsymbol{T_m}$) (*85, 86*).

The temperature dependence of the free energy of hexamer dissociation ($\boldsymbol{\Delta G}$) can be predicted at each temperature from the modified Gibbs-Helmholtz equation:

$$\boldsymbol{\Delta G(T) = \Delta H(1 - T/T_m) - \Delta C_p * \left((T_m - T) + T * \ln{(T/T_m)}\right)} \tag{7}$$

As the transition range represents only a narrow temperature range any effects of $\boldsymbol{\Delta C_p}$ (change in heat capacity) are negligible. Thus, the equilibrium constant of hexamer dissociation, *K*, is defined by

$$\boldsymbol{-R * Tln(K) = \Delta H - T * \Delta S = \Delta G} \tag{8}$$

and thus

$$\boldsymbol{K} = \boldsymbol{e}^{\left[\left(\frac{-\Delta H}{RT}\right)+\left(\frac{\Delta S}{R}\right)\right]} \tag{9}$$

Where T is the temperature in Kelvin, R is the gas constant and $\boldsymbol{\Delta S}$ is the entropy of hexamer dissociation at the transition midpoint. Therefore, when observing a two-state process by an experimental observable, $\boldsymbol{I_{obs}}$, the equilibrium constant for the reaction is:

$$\boldsymbol{K} = \frac{(\boldsymbol{I_{obs}} - (\boldsymbol{y_{trimer}} + \boldsymbol{m_{trimer}} * \boldsymbol{T}))}{((\boldsymbol{y_{hexamer}} + \boldsymbol{m_{hexamer}} * \boldsymbol{T}) - \boldsymbol{I_{obs}})} \tag{10}$$

By combining equations 8 and 10, $\boldsymbol{\Delta G}$ can be extracted (*86, 87*). The values for $\boldsymbol{\Delta H}$, $\boldsymbol{\Delta S}$, $\boldsymbol{\Delta G}$, and $\boldsymbol{T_m}$ and their respective errors were obtained by least squares minimization.

REFERENCES AND NOTES

1. F. U. Hartl, A. Bracher, M. Hayer-Hartl, Molecular chaperones in protein folding and proteostasis. *Nature* **475**, 324–332 (2011).

2. D. Balchin, M. Hayer-Hartl, F. U. Hartl, *In vivo* aspects of protein folding and quality control. *Science* **353**, aac4354 (2016).

3. J. G. Sklar, T. Wu, D. Kahne, T. J. Silhavy, Defining the roles of the periplasmic chaperones SurA, Skp, and DegP in *Escherichia coli*. *Genes Dev.* **21**, 2473–2484 (2007).

4. J. Thoma, B. M. Burmann, S. Hiller, D. J. Müller, Impact of holdase chaperones Skp and SurA on the folding of β-barrel outer-membrane proteins. *Nat. Struct. Mol. Biol.* **22**, 795–802 (2015).

5. B. M. Burmann, C. Wang, S. Hiller, Conformation and dynamics of the periplasmic membrane-protein–chaperone complexes OmpX–Skp and tOmpA–Skp. *Nat. Struct. Mol. Biol.* **20**, 1265–1272 (2013).

6. O. Subrini, J. M. Betton, Assemblies of DegP underlie its dual chaperone and protease function. *FEMS Microbiol. Lett.* **296**, 143–148 (2009).

7. J. Skórko-Glonek, A. Wawrzynów, K. Krzewski, K. Kurpierz, B. Lipińska, Site-directed mutagenesis of the HtrA (DegP) serine-protease, whose proteolytic activity is indispensable for *Escherichia coli* survival at elevated-temperatures. *Gene* **163**, 47–52 (1995).

8. R. Misra, M. CastilloKeller, M. Deng, Overexpression of protease-deficient DegP(S210A) rescues the lethal phenotype of *Escherichia coli* OmpF assembly mutants in a *degP* background. *J. Bacteriol.* **182**, 4882–4888 (2000).

9. M. CastilloKeller, R. Misra, Protease-deficient DegP suppresses lethal effects of a mutant OmpC protein by its capture. *J. Bacteriol.* **185**, 148–154 (2003).

10. M. B. Kennedy, Origin of PDZ (DHR, GLGF) domains. *Trends Biochem. Sci.* **20**, 350 (1995).

11. T. Clausen, M. Kaiser, R. Huber, M. Ehrmann, HTRA proteases: Regulated proteolysis in protein quality control. *Nat. Rev. Mol. Cell Biol.* **12**, 152–162 (2011).

12. T. Krojer, J. Sawa, E. Schäfer, H. R. Saibil, M. Ehrmann, T. Clausen, Structural basis for the regulated protease and chaperone function of DegP. *Nature* **453**, 885–890 (2008).

13. T. Krojer, M. Garrido-Franco, R. Huber, M. Ehrmann, T. Clausen, Crystal structure of DegP (HtrA) reveals a new protease-chaperone machine. *Nature* **416**, 455–459 (2002).

14. S. Kim, R. A. Grant, R. T. Sauer, Covalent linkage of distinct substrate degrons controls assembly and disassembly of DegP proteolytic cages. *Cell* **145**, 67–78 (2011).

15. N. J. Thompson, M. Merdanovic, M. Ehrmann, E. Van Duijn, A. J. R. Heck, Substrate occupancy at the onset of oligomeric transitions of DegP. *Structure* **22**, 281–290 (2014).

16. S. Li, R. Wang, D. Li, J. Ma, H. Li, X. He, Z. Chang, Y. Weng, Thermal-triggerd proteinquake leads to disassembly of DegP hexamer as an imperative activation step. *Sci. Rep.* **4**, 4834 (2015).

17. J. Jiang, X. Zhang, Y. Chen, Y. Wu, Z. H. Zhou, Z. Chang, S.-F. Sui, Activation of DegP chaperone-protease *via* formation of large cage-like oligomers upon binding to substrate proteins. *Proc. Natl. Acad. Sci. U.S.A.* **105**, 11939–11944 (2008).

18. D. Figaj, A. Gieldon, A. Polit, A. Sobiecka-Szkatula, T. Koper, M. Denkiewicz, B. Banecki, A. Lesner, J. Ciarkowski, B. Lipinska, J. Skorko-Glonek, The LA loop as an important regulatory element of the HtrA (DegP) protease from *Escherichia coli* structural and functional studies. *J. Biol. Chem.* **289**, 15880–15893 (2014).

19. T. Krojer, J. Sawa, R. Huber, T. Clausen, HtrA proteases have a conserved activation mechanism that can be triggered by distinct molecular cues. *Nat. Struct. Mol. Biol.* **17**, 844–852 (2010).

20. S. Kim, I. Song, G. Eom, S. Kim, A small periplasmic protein with a hydrophobic C-terminal residue enhances DegP proteolysis as a suicide activator. *J. Bacteriol.* **200**, e00519-17 (2018).

21. Y. Toyama, R. W. Harkness, T. Y. T. Lee, J. T. Maynes, L. E. Kay, Oligomeric assembly regulating mitochondrial HtrA2 function as examined by methyl-TROSY NMR. *Proc. Natl. Acad. Sci.* **118**, e2025022118 (2021).

22. H. Malet, F. Canellas, J. Sawa, J. Yan, K. Thalassinos, M. Ehrmann, T. Clausen, H. R. Saibil, Newly folded substrates inside the molecular cage of the HtrA chaperone DegQ. *Nat. Struct. Mol. Biol.* **19**, 152–157 (2012).

23. S. Kim, R. T. Sauer, Cage assembly of DegP protease is not required for substrate-dependent regulation of proteolytic activity or high-temperature cell survival. *Proc. Natl. Acad. Sci. U.S.A.* **109**, 7263–7268 (2012).

24. D. Lee, C. Hilty, G. Wider, K. Wüthrich, Effective rotational correlation times of proteins from NMR relaxation interference. *J. Magn. Reson.* **178**, 72–76 (2006).

25. J. J. Chou, J. L. Baber, A. Bax, Characterization of phospholipid mixed micelles by translational diffusion. *J. Biomol. NMR* **29**, 299–308 (2004).

26. K. Wüthrich, NMR assignments as a basis for structural characterization of denatured states of globular proteins. *Curr. Opin. Struct. Biol.* **4**, 93–99 (1994).

27. H. M. McConnell, Reaction rates by nuclear magnetic resonance. *J. Chem. Phys.* **28**, 430–431 (1958).

28. C. C. Valley, A. Cembran, J. D. Perlmutter, A. K. Lewis, N. P. Labello, J. Gao, J. N. Sachs, The methionine-aromatic motif plays a unique role in stabilizing protein structure. *J. Biol. Chem.* **287**, 34979–34991 (2012).

29. M. J. Plevin, D. L. Bryce, J. Boisbouvier, Direct detection of CH/π interactions in proteins. *Nat. Chem.* **2**, 466–471 (2010).

30. G. Mas, J.-Y. Guan, E. Crublet, E. C. Debled, C. Moriscot, P. Gans, G. Schoehn, P. Macek, P. Schanda, J. Boisbouvier, Structural investigation of a chaperonin in action reveals how nucleotide binding regulates the functional cycle. *Sci. Adv.* **4**, eaau4196 (2018).

31. A. G. Palmer III, C. D. Kroenke, J. P. Loria, Nuclear magnetic resonance methods for quantifying microsecond-to-millisecond motions in biological macromolecules. *Methods Enzymol.* **339**, 204–238 (2001).

32. N.-A. Lakomek, J. Ying, A. Bax, Measurement of ^{15}N relaxation rates in perdeuterated proteins by TROSY-based methods. *J. Biomol. NMR* **53**, 209–221 (2012).

33. B. M. Burmann, U. Scheckenhofer, K. Schweimer, P. Rösch, Domain interactions of the transcription-translation coupling factor *Escherichia coli* NusG are intermolecular and transient. *Biochem. J.* **435**, 783–789 (2011).

34. B. M. Burmann, S. H. Knauer, A. Sevostyanova, K. Schweimer, R. A. Mooney, R. Landick, I. Artsimovitch, P. Rösch, An α helix to β barrel domain switch transforms the transcription factor RfaH into a translation factor. *Cell* **150**, 291–303 (2012).

35. G. Lipari, A. Szabo, Model-Free approach to the interpretation of nuclear magnetic resonance relaxation in macromolecules. 2. Analysis of experimental results. *J. Am. Chem. Soc.* **104**, 4559–4570 (1982).

36. G. M. Clore, A. Szabo, A. Bax, L. E. Kay, P. C. Driscoll, A. M. Gronenborn, Deviations from the simple two-parameter model-free approach to the interpretation of nitrogen-15 nuclear magnetic relaxation of proteins. *J. Am. Chem. Soc.* **112**, 4989–4991 (1990).

37. L. Morgado, B. M. Burmann, T. Sharpe, A. Mazur, S. Hiller, The dynamic dimer structure of the chaperone Trigger Factor. *Nat. Commun.* **8**, 1992 (2017).

38. L. He, T. Sharpe, A. Mazur, S. Hiller, A molecular mechanism of chaperone-client recognition. *Sci. Adv.* **2**, e1601625 (2016).

39. R. G. Parra, N. P. Schafer, L. G. Radusky, M. Y. Tsai, A. B. Guzovsky, P. G. Wolynes, D. U. Ferreiro, Protein Frustratometer 2: A tool to localize energetic frustration in protein molecules, now with electrostatics. *Nucleic Acids Res.* **44**, W356–W360 (2016).

40. D. M. Korzhnev, K. Kloiber, V. Kanelis, V. Tugarinov, L. E. Kay, Probing slow dynamics in high molecular weight proteins by methyl-TROSY NMR spectroscopy: Application to a 723-residue enzyme. *J. Am. Chem. Soc.* **126**, 3964–3973 (2004).

41. A. K. Lewis, K. M. Dunleavy, T. L. Senkow, C. Her, B. T. Horn, M. A. Jersett, R. Mahling, M. R. McCarthy, G. T. Perell, C. C. Valley, C. B. Karim, J. Gao, W. C. K. Pomerantz, D. D. Thomas, A. Cembran, A. Hinderliter, J. N. Sachs, Oxidation increases the strength of the methionine-aromatic interaction. *Nat. Chem. Biol.* **12**, 860–866 (2016).

42. A. Fujii, H. Hayashi, J. W. Park, T. Kazama, N. Mikami, S. Tsuzuki, Experimental and theoretical determination of the accurate CH/π interaction energies in benzene-alkane clusters: Correlation between interaction energy and polarizability. *Phys. Chem. Chem. Phys.* **13**, 14131–14141 (2011).

43. R. Sprangers, A. Gribun, P. M. Hwang, W. A. Houry, L. E. Kay, Quantitative NMR spectroscopy of supramolecular complexes: Dynamic side pores in ClpP are important for product release. *Proc. Natl. Acad. Sci. U.S.A.* **102**, 16678–16683 (2005).

44. S. Vahidi, Z. A. Ripstein, J. B. Juravsky, E. Rennella, A. L. Goldberg, A. K. Mittermaier, J. L. Rubinstein, L. E. Kay, An allosteric switch regulates *Mycobacterium tuberculosis ClpP1P2* protease function as established by cryo-EM and methyl-TROSY NMR. *Proc. Natl. Acad. Sci. U.S.A.* **117**, 5895–5906 (2020).

45. M. E. Lee, T. A. Baker, R. T. Sauer, Control of substrate gating and translocation into ClpP by channel residues and ClpX binding. *J. Mol. Biol.* **399**, 707–718 (2010).

46. L. Truebestein, A. Tennstaedt, T. Mönig, T. Krojer, F. Canellas, M. Kaiser, T. Clausen, M. Ehrmann, Substrate-induced remodeling of the active site regulates human HTRA1 activity. *Nat. Struct. Mol. Biol.* **18**, 386–388 (2011).

47. R. V. Mauldin, R. T. Sauer, Allosteric regulation of DegS protease subunits through a shared energy landscape. *Nat. Chem. Biol.* **9**, 90–96 (2013).

48. C. Wilken, K. Kitzing, R. Kurzbauer, M. Ehrmann, T. Clausen, Crystal structure of the DegS stress sensor. *Cell* **117**, 483–494 (2004).

49. J. Sawa, H. Malet, T. Krojer, F. Canellas, M. Ehrmann, T. Clausen, Molecular adaptation of the DegQ protease to exert protein quality control in the bacterial cell envelope. *J. Biol. Chem.* **286**, 30680–30690 (2011).

50. B. J. Hillier, K. S. Christopherson, K. E. Prehoda, D. S. Bredt, W. A. Lim, Unexpected modes of PDZ domain scaffolding revealed by structure of nNOS-syntrophin complex. *Science* **284**, 812–815 (1999).

51. S. Srivastava, P. Osten, F. S. Vilim, L. Khatri, G. Inman, B. States, C. Daly, S. DeSouza, R. Abagyan, J. G. Valtschanoff, R. J. Weinberg, E. B. Ziff, Novel anchorage of GluR2/3 to the postsynaptic density by the AMPA receptor-binding protein ABP. *Neuron* **21**, 581–591 (1998).

52. R. Schlecht, S. R. Scholz, H. Dahmen, A. Wegener, C. Sirrenberg, D. Musil, J. Bomke, H. M. Eggenweiler, M. P. Mayer, B. Bukau, Functional analysis of Hsp70 inhibitors. *PLOS ONE* **8**, e78443 (2013).

53. J. Mikolajczyk, M. Drag, M. Békés, J. T. Cao, Z. Ronai, G. S. Salvesen, Small Ubiquitin-related Modifier (SUMO)-specific proteases: Profiling the specificities and activities of human SENPs. *J. Biol. Chem.* **282**, 26217–26224 (2007).

54. J. Sambrook, E. F. Fritsch, T. Maniatis, *Molecular Cloning: A Laboratory Manual* (Cold Spring Harbor Laboratory, 1989), vol. 2.

55. K. Pervushin, R. Riek, G. Wider, K. Wüthrich, Attenuated T_2 relaxation by mutual cancellation of dipole-dipole coupling and chemical shift anisotropy indicates an avenue to NMR structures of very large biological macromolecules in solution. *Proc. Natl. Acad. Sci. U.S.A.* **94**, 12366–12371 (1997).

56. M. Salzmann, K. Pervushin, G. Wider, H. Senn, K. Wüthrich, TROSY in triple-resonance experiments: New perspectives for sequential NMR assignment of large proteins. *Proc. Natl. Acad. Sci. U.S.A.* **95**, 13585–13590 (1998).

57. M. Sattler, J. Schleucher, C. Griesinger, Heteronuclear multidimensional NMR experiments for the structure determination of proteins in solution employing pulsed field gradients. *Prog. Nucl. Magn. Reson. Spectrosc.* **34**, 93–158 (1999).

58. P. Rossi, Y. Xia, N. Khanra, G. Veglia, C. G. Kalodimos, ^{15}N and ^{13}C- SOFAST-HMQC editing enhances 3D-NOESY sensitivity in highly deuterated, selectively [^{1}H,^{13}C]-labeled proteins. *J. Biomol. NMR* **66**, 259–271 (2016).

59. G. Wider, L. Dreier, Measuring protein concentrations by NMR spectroscopy. *J. Am. Chem. Soc.* **128**, 2571–2576 (2006).

60. F. Delaglio, S. Grzesiek, G. W. Vuister, G. Zhu, J. Pfeifer, A. Bax, NMRPipe: A multidimensional spectral processing system based on UNIX pipes. *J. Biomol. NMR* **6**, 277–293 (1995).

61. V. Jaravine, I. Ibraghimov, V. Y. Orekhov, Removal of a time barrier for high-resolution multidimensional NMR spectroscopy. *Nat. Methods* **3**, 605–607 (2006).

62. R. L. J. Keller, *The Computer Aided Resonance Assignment Tutorial* (Cantina Verlag, Goldau, 2004).

63. J. T. Nielsen, F. A. A. Mulder, Potenci: Prediction of temperature, neighbor and pH-corrected chemical shifts for intrinsically disordered proteins. *J. Biomol. NMR* **70**, 141–165 (2018).

64. G. Zhu, Y. Xia, L. K. Nicholson, K. H. Sze, Protein dynamics measurements by TROSY-based NMR experiments. *J. Magn. Reson.* **143**, 423–426 (2000).

65. T. Szyperski, P. Lunginbühl, G. Otting, P. Güntert K. Wüthrich, Protein dynamics studied by rotating frame ^{15}N spin relaxation-times. *J. Biomol. NMR* **3**, 151–164 (1993).

66. T. A. Walton, C. M. Sandoval, C. A. Fowler, A. Pardi, M. C. Sousa, The cavity-chaperone Skp protects its substrate from aggregation but allows independent folding of substrate domains. *Proc. Natl. Acad. Sci. U.S.A.* **106**, 1772–1777 (2009).

67. P. Dosset, J. C. Hus, M. Blackledge, D. Marion, Efficient analysis of macromolecular rotational diffusion from heteronuclear relaxation data. *J. Biomol. NMR* **16**, 23–28 (2000).

68. M. W. Maciejewski, A. D. Schuyler, M. R. Gryk, I. I. Moraru, P. R. Romero, E. L. Ulrich, H. R. Eghbalnia, M. Livny, F. Delaglio, J. C. Hoch, NMRbox: A Resource for biomolecular NMR computation. *Biophys. J.* **112**, 1529–1534 (2017).

69. P. Eastman, M. S. Friedrichs, J. D. Chodera, R. J. Radmer, C. M. Bruns, J. P. Ku, K. A. Beauchamp, T. J. Lane, L. P. Wang, D. Shukla, T. Tye, M. Houston, T. Stich, C. Klein, M. R. Shirts, V. S. Pande, OpenMM 4: A reusable, extensible, hardware independent library for high performance molecular simulation. *J. Chem. Theory Comput.* **9**, 461–469 (2013).

70. J. García De La Torre, M. L. Huertas, B. Carrasco, Calculation of hydrodynamic properties of globular proteins from their atomic-level structure. *Biophys. J.* **78**, 719–730 (2000).

71. H. Sun, L. E. Kay, V. Tugarinov, An optimized relaxation-based coherence transfer NMR experiment for the measurement of side-chain order in methyl-protonated, highly deuterated proteins. *J. Phys. Chem. B* **115**, 14878–14884 (2011).

72. K. Weinhäupl, C. Lindau, A. Hessel, Y. Wang, C. Schütze, T. Jores, L. Melchionda, B. Schönfisch, H. Kalbacher, B. Bersch, D. Rapaport, M. Brennich, K. Lindorff-Larsen, N. Wiedemann, P. Schanda, Structural basis of membrane protein chaperoning through the mitochondrial intermembrane space. *Cell* **175**, 1365–1379.e25 (2018).

73. N. A. Farrow, O. Zhang, J. D. Forman-Kay, L. E. Kay, A heteronuclear correlation experiment for simultaneous determination of ^{15}N longitudinal decay and chemical exchange rates of systems in slow equilibrium. *J. Biomol. NMR* **4**, 727–734 (1994).

74. P. Kiraly, I. Swan, M. Nilsson, G. A. Morris, Improving accuracy in DOSY and diffusion measurements using triaxial field gradients. *J. Magn. Reson.* **270**, 24–30 (2016).

75. C. H. Cho, J. Urquidi, S. Singh, G. Wilse Robinson, Thermal offset viscosities of liquid H_2O, D_2O and T_2O. *J. Phys. Chem. B*, **103**, 1991–1994 (1999)

76. C. Arquint, A.-M. Gabryjonczyk, S. Imseng, R. Böhm, E. Sauer, S. Hiller, E. A. Nigg, T. Maier, STIL binding to Polo-box 3 of PLK4 regulates centriole duplication. *eLife* **4**, e07888 (2015).

77. A. S. Altieri, R. A. Byrd, D. P. Hinton, Association of biomolecular systems *via* pulsed field gradient NMR self-diffusion measurements. *J. Am. Chem. Soc.* **117**, 7566–7567 (1995).

78. L. Shi, L. E. Kay, Tracing an allosteric pathway regulating the activity of the HslV protease. *Proc. Natl. Acad. Sci. U.S.A.* **111**, 2140–2145 (2014).

79. B. M. Burmann, J. A. Gerez, I. Matečko-Burmann, S. Campioni, P. Kumari, D. Ghosh, A. Mazur, E. E. Aspholm, D. Šulskis, M. Wawrzyniuk, T. Bock, A. Schmidt, S. G. D. Rüdiger, R. Riek, S. Hiller, Regulation of α-synuclein by chaperones in mammalian cells. *Nature* **577**, 127–132 (2020).

80. G. Lipari, A. Szabo, Model-Free Approach to the interpretation of nuclear magnetic resonance relaxation in macromolecules. 1. Theory and range of validity. *J. Am. Chem. Soc.* **104**, 4546–4559 (1982).

81. M. Landau, I. Mayrose, Y. Rosenberg, F. Glaser, E. Martz, T. Pupko, N. Ben-Tal, ConSurf 2005: The projection of evolutionary conservation scores of residues on protein structures. *Nucleic Acids Res.* **33**, W299–W302 (2005).

82. H. Ashkenazy, E. Erez, E. Martz, T. Pupko, N. Ben-Tal, ConSurf 2010: Calculating evolutionary conservation in sequence and structure of proteins and nucleic acids. *Nucleic Acids Res.* **38**, W529–W533 (2010).

83. K. E. Neet, D. E. Timm, Conformational stability of dimeric proteins: Quantitative studies by equilibrium denaturation. *Protein Sci.* **3**, 2167–2174 (1994).

84. B. M. Burmann, X. Luo, P. Rösch, M. C. Wahl, M. E. Gottesman, Fine tuning of the *E. coli* NusB:NusE complex affinity to BoxA RNA is required for processive antitermination. *Nucleic Acids Res.* **38**, 314–326 (2009).

85. M. M. Santoro, D. W. Bolen, Unfolding free energy changes determined by the linear extrapolation method. 1. Unfolding of phenylmethanesulfonyl α-chymotrypsin using different denaturants. *Biochemistry* **27**, 8063–8068 (1988).

86. L. Swint, A. D. Robertson, Thermodynamics of unfolding for turkey ovomucoid third domain: Thermal and chemical denaturation. *Protein* Sci. **2**, 2037–2049 (1993).

87. L. M. Mayr, O. Landt, U. Hahn, F. X. Schmid, Stability and folding kinetics of ribonuclease T1 are strongly altered by the replacement of Cis-proline 39 with alanine. *J. Mol. Biol.* **231**, 897–912 (1993).
